# Systematic Inference of Copy-Number Genotypes from Personal Genome Sequencing Data Reveals Extensive Olfactory Receptor Gene Content Diversity

**DOI:** 10.1371/journal.pcbi.1000988

**Published:** 2010-11-11

**Authors:** Sebastian M. Waszak, Yehudit Hasin, Thomas Zichner, Tsviya Olender, Ifat Keydar, Miriam Khen, Adrian M. Stütz, Andreas Schlattl, Doron Lancet, Jan O. Korbel

**Affiliations:** 1 Department of Molecular Genetics, Crown Human Genome Center, Weizmann Institute of Science, Rehovot, Israel; 2 Department of Biotechnology and Bioinformatics, Weihenstephan-Triesdorf University of Applied Sciences, Freising, Germany; 3 Genome Biology Research Unit, European Molecular Biology Laboratory (EMBL), Heidelberg, Germany; 4 European Bioinformatics Institute, EMBL-EBI, Hinxton, United Kingdom; University of British Columbia, Canada

## Abstract

Copy-number variations (CNVs) are widespread in the human genome, but comprehensive assignments of integer locus copy-numbers (*i.e.*, copy-number genotypes) that, for example, enable discrimination of homozygous from heterozygous CNVs, have remained challenging. Here we present CopySeq, a novel computational approach with an underlying statistical framework that analyzes the depth-of-coverage of high-throughput DNA sequencing reads, and can incorporate paired-end and breakpoint junction analysis based CNV-analysis approaches, to infer locus copy-number genotypes. We benchmarked CopySeq by genotyping 500 chromosome 1 CNV regions in 150 personal genomes sequenced at low-coverage. The assessed copy-number genotypes were highly concordant with our performed qPCR experiments (Pearson correlation coefficient 0.94), and with the published results of two microarray platforms (95–99% concordance). We further demonstrated the utility of CopySeq for analyzing gene regions enriched for segmental duplications by comprehensively inferring copy-number genotypes in the CNV-enriched >800 olfactory receptor (OR) human gene and pseudogene loci. CopySeq revealed that OR loci display an extensive range of locus copy-numbers across individuals, with zero to two copies in some OR loci, and two to nine copies in others. Among genetic variants affecting OR loci we identified deleterious variants including CNVs and SNPs affecting ∼15% and ∼20% of the human OR gene repertoire, respectively, implying that genetic variants with a possible impact on smell perception are widespread. Finally, we found that for several OR loci the reference genome appears to represent a minor-frequency variant, implying a necessary revision of the OR repertoire for future functional studies. CopySeq can ascertain genomic structural variation in specific gene families as well as at a genome-wide scale, where it may enable the quantitative evaluation of CNVs in genome-wide association studies involving high-throughput sequencing.

## Author Summary

Human individual genome sequencing has recently become affordable, enabling highly detailed genetic sequence comparisons. While the identification and genotyping of single-nucleotide polymorphisms has already been successfully established for different sequencing platforms, the detection, quantification and genotyping of large-scale copy-number variants (CNVs), *i.e.*, losses or gains of long genomic segments, has remained challenging. We present a computational approach that enables detecting CNVs in sequencing data and accurately identifies the actual copy-number at which DNA segments of interest occur in an individual genome. This approach enabled us to obtain novel insights into the largest human gene family – the olfactory receptors (ORs) – involved in smell perception. While previous studies reported an abundance of CNVs in ORs, our approach enabled us to globally identify absolute differences in OR gene counts that exist between humans. While several OR genes have very high gene counts, other ORs are found only once or are missing entirely in some individuals. The latter have a particularly high probability of influencing individual differences in the perception of smell, a question that future experimental efforts can now address. Furthermore, we observed differences in OR gene counts between populations, pointing at ORs that might contribute to population-specific differences in smell.

## Introduction

Structural variants in the human genome, such as CNVs or balanced inversions, represent a major form of variation with widespread functional consequences [1]. Numerous surveys mapping CNVs at varying levels of resolution [2], [3], [4], [5], [6] have created a comprehensive CNV inventory, with the latest survey reporting 1,098 CNVs on average between two individuals spanning nearly 0.8% of the genome [6]. Collectively, the list of reported CNVs presently involves 8,410 loci (Database of Genomic Variants [3], DGV) when applying the frequently used operational definition for CNVs, *i.e.*, gains and losses of segments 1 kb or larger in size [7].

Recent studies have associated CNVs with various phenotypes, including benign and disease-related phenotypes such as cancer, HIV-1/AIDS susceptibility, autoimmunity, and complex disorders ([1] and references therein). Yet, while different conceptual approaches for CNV-discovery have been developed [8], [9], [10], [11], [12], [13] most CNV analysis approaches presently do not distinguish CNVs based on the copy-number of the underlying DNA segment, *i.e.*, its *copy-number genotype*, a distinction that is crucial for leveraging CNV assignments for studies focusing on genome evolution and genotype-phenotype associations [14]. For example, copy-number genotypes enable distinguishing *bi-allelic* loci (*i.e.*, loci at which in addition to the reference allele either a single duplication or a single deletion allele is observed) from *multi-allelic* loci (*i.e.*, loci with more than one variant, such as deletion and duplication, or multiple duplications). Furthermore, in bi-allelic loci copy-number genotypes allow discriminating *heterozygous* from *homozygous* CNVs. Such information is crucial in association studies, where the failure to assign locus copy-numbers or to discriminate heterozygotes from homozygotes limits the statistical power. Recently, improvements in microarray technology have led to advances in CNV analysis by facilitating the ascertainment of copy-number genotypes in genomic regions amenable to hybridization by high-resolution comparative genome hybridization (array-CGH) or state-of-the-art SNP/CNV hybrid array platforms [6], [14]. While microarrays have advantages in enabling CNV ascertainment at high-throughput and low-cost, their resolution can be limited in CNV-rich regions involving segmental duplications [15] (SDs). This might be because of probe cross-hybridization issues, which may reduce the number of effective oligonucleotide probes that can be designed for these regions [16]. Indeed, commercial microarray-based approaches for copy-number genotyping are restricted to genomic loci for which probes are available at sufficient densities [6], [14], while custom array designs may compensate for probe densities with the remaining limitation of relying on regions for which effective probes can be designed.

Recent breakthroughs in ‘Next Generation Sequencing’ (NGS) technologies have stimulated the development of computational approaches that enable the discovery of CNVs with excellent quantitative and spatial resolution [8], [9], [10], [11], [13]. In this regard, several studies have demonstrated that the sequencing depth-of-coverage of NGS reads can be employed for CNV-discovery [8], [9], [17], [18], [19]. For example, Xie *et al.* [18] and Chiang *et al.* [8] described CNV-discovery approaches conceptually related to array-CGH analysis, whereby the read-depth in genomic intervals is compared between pairs of samples to detect CNVs as relational changes in studies involving case/reference setups (*e.g.*, cancer tissue *vs.* healthy tissue). Furthermore, Alkan *et al.* recently reported an elegant read-count based approach for mapping locus copy-number differences in large (≥20 kb) SDs using high-coverage (6 to 20-fold coverage) NGS data, by equating averaged and rounded read-counts in individual samples with integer locus copy-numbers [19]. However, with recent advances enabling sequencing hundreds of genomes in studies focused on population genetics or genotype-phenotype correlations, a statistical framework for copy-number genotyping will soon become a prerequisite to enable the probabilistic ascertainment of CNV sets in NGS-based association studies. To be useful for genome-wide association studies, a NGS-based copy-number genotyping approach needs to provide absolute locus copy-number estimates in a sample-specific manner and needs to be able to determine confidence values for each copy-number genotype (to maximize statistical power). Furthermore, it should enable accurate ascertainment of a wide range of CNVs, including rare and common ones, and including those at the 1–20 kb size-range, a highly abundant CNV size-class [4]. Lastly, the ability to utilize low-coverage (*i.e.*, ≤4× coverage) NGS datasets, *i.e.*, datasets such as the ones generated by the ‘1000 Genomes Project’ (1000GP; see http://1000genomes.org), will be a crucial asset for such a copy-number genotyping approach, given that sample number and sequencing coverage will be at a constant tradeoff in future association studies.

Here, we present CopySeq, a statistical framework for copy-number genotype inference from low-coverage genomes, which is available at http://embl.de/~korbel/copyseq/. As a benchmark we used CopySeq to genotype a set of CNVs previously analyzed with microarrays and obtained excellent genotyping concordances for CNVs across a wide size-range. In addition, as a proof-of-principle we used the approach to infer copy-number genotypes in the largest human gene family, with many genes and pseudogenes embedded within SDs: *i.e.*, we analyzed the >800 olfactory receptor (OR) genes and pseudogenes in the human genome. OR genes form one of the most genetically variable and rapidly evolving protein-coding gene families and display a strong enrichment for CNVs [4], [20], [21], [22] compared to most other gene families. Thus, the OR gene family represents an appealing model for assessing copy-number genotype ascertainment using low-coverage sequencing and for studying the effect of CNVs on protein coding loci. Owing to the comparative nature of earlier studies, CNVs in ORs were thus far mostly reported as *gains* and *losses* relative to an arbitrarily chosen reference sample, and for most ORs no absolute locus copy-number assignments have been reported so far. Thus, the full nature and extent of copy-number variation in ORs remained unknown. Notably, it is presently unclear to what degree single deletions or duplications (*i.e.*, bi-allelic) or multiple recurrent CNV-formation events (*i.e.*, multi-allelic) affect particular OR loci, an information that is crucial for functional analyses as multiple alleles can reduce signals in association studies. Our analysis of ORs using CopySeq revealed a widespread diversity in integer locus copy-numbers in human OR loci in the 150 individuals assessed. We report a segregation of copy-number variable OR loci into bi-allelic, multi-allelic, and non-variable CNVs, with notable population differences in some OR loci. In addition, our analysis enabled us to address and further dissect genomic biases that may influence the extent of CNVs affecting ORs, including functional (genes *vs.* pseudogenes), DNA sequence context (non-repetitive *vs.* repetitive DNA), and evolutionary (‘*young*’ *vs.* ‘*ancient*’ ORs) biases.

## Results

### A statistical framework for copy-number genotyping in NGS data

**Figure 1:**
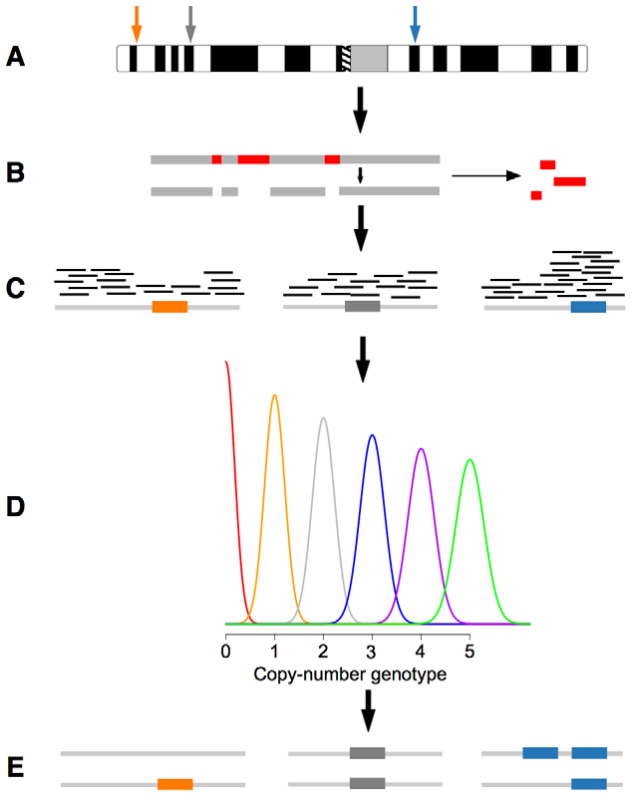
Schematic illustration of CopySeq. **A**. ‘*Locus selection*’, *i.e.*, definition and selection of loci of interest for copy-number genotyping. **B**. ‘*Mappability assessment*’, *i.e.*, construction of *k-mer* mappability locus maps. Sequence sub-stretches not uniquely mappable by *k-mers* are identified in each locus (represented by red blocks) and masked (*i.e.*, excluded from further analysis). **C**. ‘*Read-mapping*’, by default carried out with MAQ [24] (other read-mappers, such as BWA [23] can optionally be applied). **D**. ‘*Copy-number genotyping*’: The locus-specific read-depth is determined, and the locus-specific ‘read-depth ratio’ computed and corrected both for the locus-specific *k-mer* mappability as well as for G+C-content bias (see Materials and Methods). A Gaussian classifier infers locus copy numbers by comparing locus-specific read-depth ratios with read-depth ratio distributions which are expected for different copy-number genotypes (distributions for the copy-number genotypes 0, 1, 2, 3, 4, and 5 are indicated with different colors). **E**. Copy-number genotypes are reported.

CopySeq enables the inference of copy-number genotypes in genomic loci suspected to differ in copy-number (see Materials and Methods for details, and Figure 1). The first step undertaken by CopySeq, termed *locus selection*, involves the definition of putative CNV loci. Loci may be selected based on biological considerations, *e.g.*, to enable copy-number genotyping comprehensively in previously published CNV sets, or in a more focused manner in candidate loci of an association study. Following locus selection, the *mappability assessment* step assesses the mappability of all *k*-mer subsequences of the selected loci to identify sequence stretches that are unambiguously (uniquely) mappable with short reads, *i.e.*, such with no exact duplicate sequence in the reference genome. This step assures exclusion of (1) unspecific reads and (2) reads originating from paralogous sequences. Then, in the *read-mapping* step DNA reads are aligned onto the reference genome, and only unique matches retained, using a fast read-mapper such as the MAQ or BWA algorithm [23], [24]. Lastly, the *copy-number genotyping* step measures the locus-specific read-depth in the mappable sequence fraction and infers copy-number genotypes for each sample by relating the resulting read-depth value to the expected locus read-depth after correction for GC-content biases [25] using a smoothing spline-based approach. In particular, CopySeq uses a Gaussian classifier that regards discrete locus copy-numbers as probability distributions and infers copy-number genotypes with confidence scores (see Materials and Methods). The copy-number genotype thereby indicates the diploid copy-number of a locus of interest in a given genome. In the case of bi-allelic CNVs, this enables distinguishing homozygous from heterozygous CNVs (*e.g.*, heterozygous deletion = ‘1’ copy; homozygous deletion = ‘0’ copies; homozygous reference allele, or ‘no deletion’ = ‘2’ copies).

Optionally, CopySeq incorporates redefined boundaries (or breakpoints) of CNVs, available for confined CNV subsets [26], prior to the *copy-number genotyping* step by applying different conceptual approaches: *i.e.*, ‘paired-end mapping’ (PEM), which identifies CNVs from paired reads that map abnormally onto the reference genome [4]; or ‘breakpoint-junction sequence analysis’ (BJA), which detects CNVs by aligning sequence reads onto CNV breakpoint-junctions [26]. The rationale for applying such boundary-redefinition approaches is that accurate (*i.e.*, redefined) CNV-boundaries facilitate the proper interpretation of read-depth data, thus enabling more accurate copy-number genotype inference (see below).

### Data source

To assess the performance of CopySeq on low-coverage genome sequences we acquired NGS data from 150 individuals with different ancestries, *i.e.*, genomes that were recently sequenced at low coverage in the 1000GP pilot phase 1 (Table S1 and Materials and Methods): 52 unrelated African individuals with ancestry from Nigeria (Yoruba from Ibadan; YRI); 53 Asians, including 29 unrelated Chinese individuals from Beijing (CHB) and 24 unrelated Japanese from Tokyo (JPT; we analyzed all 53 Asian individuals together as the “CHB+JPT” group [27]); and 45 individuals of European ancestry from Utah (CEU), USA, including 42 unrelated individuals and 3 members of a parent-offspring trio. The analyzed genomes were sequenced at 3–4× fold coverage on average; most reads had a read-length of 36 nt.

### Assessing the genotyping concordance of 500 CNVs on chromosome 1

**Figure 2:**
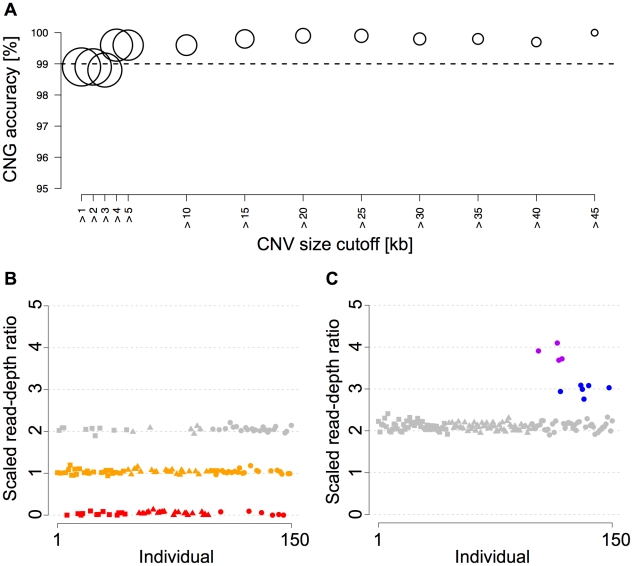
Copy-number genotyping results in a chromosome 1 CNV set. **A**. Copy-number genotyping concordance between CopySeq- and microarray-based [14] copy-number genotypes inferred for 99 CNVs on chromosome 1 in 118 individuals, using different CNV size cutoffs. Plotted circles represent the total number of high-confidence genotypes, with the largest circle corresponding to >10,000 copy-number genotypes and the smallest to 348 copy-number genotypes. As expected, the genotyping concordance increases with higher CNV size cutoffs. **B–C**. Copy-number genotyping results for chromosome 1 example CNVs across 150 individuals, *i.e.*, a bi-allelic deletion (chr1:150,822,330–150,853,218; see **B**) as well as a bi-allelic duplication (chr1:164,451,105–164,460,994; see **C**). Copy-number genotypes inferred by CopySeq are indicated with different colors: ‘0’, red; ‘1’, orange; ‘2’, grey; ‘3’, blue; ‘4’, purple. Individuals have been arranged according to population: squares, CEU; triangles, CHB+JPT; circles, YRI. The scaled read-depth ratio (indicated on the y-axis) has been calculated by multiplying the read-depth ratio by two.

To evaluate CopySeq, we first assembled known CNVs from human chromosome 1, for which copy-number genotypes were previously inferred with Affymetrix SNP 6.0 microarrays [14] (a SNP/CNV hybrid microarray platform). Namely, we compared CopySeq with copy-number genotypes from McCarroll *et al.*, who analyzed 270 individuals out of which 118 overlapped with our study, to initially evaluate CopySeq (see Materials and Methods). Out of 100 CNVs [14] with a median size of 6.8 kb (mean = 11.2 kb), only one CNV displayed less than 500 mappable 36-mers and thus was excluded (Materials and Methods). We found that most CNV loci were covered by an appreciable number of sequencing reads, with a mean of 685 reads (median = 418). CopySeq was used to generate 14,850 (99 loci times 150 samples) copy-number genotypes in this CNV set, with inferred locus copy-numbers ranging from ‘0’ copies up to ‘5’ copies (Table S5 and Figures S8, S9). The copy-number genotypes displayed an excellent genotyping concordance of 98.9% with the Affymetrix-array based results (Tables S3, S6, S7; Figures 2ABC, S18A). Note that by assuming that array-based genotypes are correct, genotyping concordances achieved with CopySeq can be considered as lower bound estimates for genotyping accuracies (note that discordances in specific genotypes could obviously be either due to errors in the array-based genotypes or due to errors in CopySeq's genotypes). In general, deletion genotypes inferred by CopySeq yielded higher concordances than duplication genotypes. We quantified this by calculating the positive predictive value (PPV) for deletions (99.6%) and duplications (89.1%) (see Table S8), suggesting that while both deletions and duplications are identified at high accuracy, duplications are more difficult to ascertain than deletions by CopySeq, microarray-based genotyping, or both methods.

We also estimated genotyping concordances for various different CNV size cutoffs (Figure 2A), and, as expected, observed an increase in concordance between CopySeq and Affymetrix-arrays with CNV size. However, high concordances were obtained also for relatively small CNVs (*e.g.*, 97.5% for CNVs 1–2 kb in size), suggesting the applicability of CopySeq across a wide CNV size-range in 3–4× coverage sequence data.

Furthermore, we combined CopySeq with PEM and BJA (Materials and Methods), and found that CNV-boundary redefinition with PEM or BJA leads to improved genotyping concordances. Specifically, when applying PEM we measured a genotyping concordance with Affymetrix-arrays of 99.6%, and when applying BJA we measured a concordance of 99.7% (Tables S2, S9, S10) – although the numbers of CNVs ascertained was comparably low for BJA and PEM, as for many loci the CNV-boundaries were unknown. A further advantage of CNV-boundary redefinition is that it can help untangle complex CNV loci, as we exemplified below.

We also compared CopySeq's copy-number genotypes to genotypes recently inferred with a high-resolution Agilent oligonucleotide array-CGH platform [6] through analyzing 401 CNV regions on chromosome 1 with a median size of 3.1 kb. Conrad *et al.* [6] analyzed 450 individuals out of which 149 overlapped with our study. Our analysis resulted in an excellent, albeit slightly weaker genotyping concordance of 89.1% – with small CNVs displaying higher concordances than large CNVs and some CNVs displaying 0% concordance (Tables S15; Figure S18B). When looking for the source of the discordance we found that many of the disagreeing copy-number genotypes occurred in large (>10 kb) CNVs embedded in SDs (see Text S1). Altogether, four of these large CNVs were ascertained both by the Agilent and the Affymetrix platform. We found that whereas in all four regions the Agilent CGH arrays tended to agree with CopySeq in a relative sense (mean Pearson correlation coefficient 0.78), Agilent array-CGH was for all four CNVs discordant with both the Affymetrix SNP arrays and CopySeq in terms of the absolute copy-number reported (note that absolute copy-numbers agreed between CopySeq and Affymetrix arrays in these regions; see Table S17). To improve the comparability between the platforms, we thus reasonably excluded CNVs intersecting with SDs when analyzing the Agilent array-CGH based results. This led to an overall improved copy-number genotyping concordance of 94.8% between CopySeq and Agilent array-CGH (Table S16).

Furthermore, we compared CopySeq to Alkan *et al.*'s approach [19], which interprets averaged read-depths as locus copy-numbers (*i.e.*, depth-of-coverage analysis without probabilistic genotyping model). We used CopySeq to analyze published short sequence reads from a single African male individual [28] which previously had been analyzed with regard to copy-number variation [19], and obtained a better concordance with Affymetrix SNP array-based copy-numbers for CopySeq (97.2%) than for depth-of-coverage analysis without genotyping model (80.2%). As suggested in [19] these concordance estimations excluded SD regions, as Alkan *et al.*'s approach infers copy-numbers in SDs as a genome-wide sum across all paralogous loci, rather than separately for each paralog (see Text S1 and Table S18).

Next, we randomly picked eighteen common CNVs and subjected them to quantitative PCR (qPCR) validation in three individuals each. The CNV sizes ranged from 707 bp to 127 kb (median size 3.7 kb) and most copy-number genotypes (65%) in these eighteen regions differed from the homozygous reference copy-number of ‘2’. In total, 49 out of 54 (91%) copy-number genotypes were supported by the qPCR experiments, results that were in good agreement with the genotyping concordances determined based on the microarray platforms (see Text S1 and Table S19). We further compared CopySeq's results to a set of loci that had previously been analyzed by fluorescent *in situ* hybridization (FISH) [19], with the FISH results validating CopySeq's copy-number genotypes in four out of five assessed loci (see Text S1 and Table S21).

Furthermore, we tested the effect of sequencing coverage on CopySeq's performance by generating sub-coverage datasets (0.5, 1, 2, 3, 4, 5, 10, 20, and 30×) of the high-coverage (∼40×) NA18507 genome [28] and assessed to what extent the genotype concordance with two complementary microarray platforms changed with coverage (see Text S1). Although, unsurprisingly, genotyping accuracies improved with increasing coverage, low-coverage (3–4×) genomes displayed only a minor decrease in accuracy compared to a 30–40× genome (0.8–1.6%; see Figure S16), suggesting low-coverage sequencing offers an excellent tradeoff between cost, throughput, and sensitivity in variant detection. We also assessed the effects sequencing errors may have on CopySeq's genotypes in low-coverage data, and found their influence to be minor (see Text S1).

### Measuring copy-number genotypes in olfactory receptor (OR) loci across 150 individuals

Our initial assessment of CopySeq suggested an excellent accuracy in genomic regions ascertained with microarrays. Given that in principle any genomic region mappable by unique sequence reads can be analyzed with CopySeq, we next specifically assessed CopySeq's performance in a set of relatively hard-to-ascertain regions. Namely, as a proof-of-concept, we assessed genomic loci associated with the largest human gene family – *i.e.*, the 388 OR gene and 463 OR pseudogene loci, most of which are not ascertained by state-of-the-art commercial genotyping array platforms [14]. In particular, we reasoned that CopySeq may enable the first comprehensive assessment of the extent of variation in terms of integer locus-copy numbers in the OR gene family.

**Figure 3:**
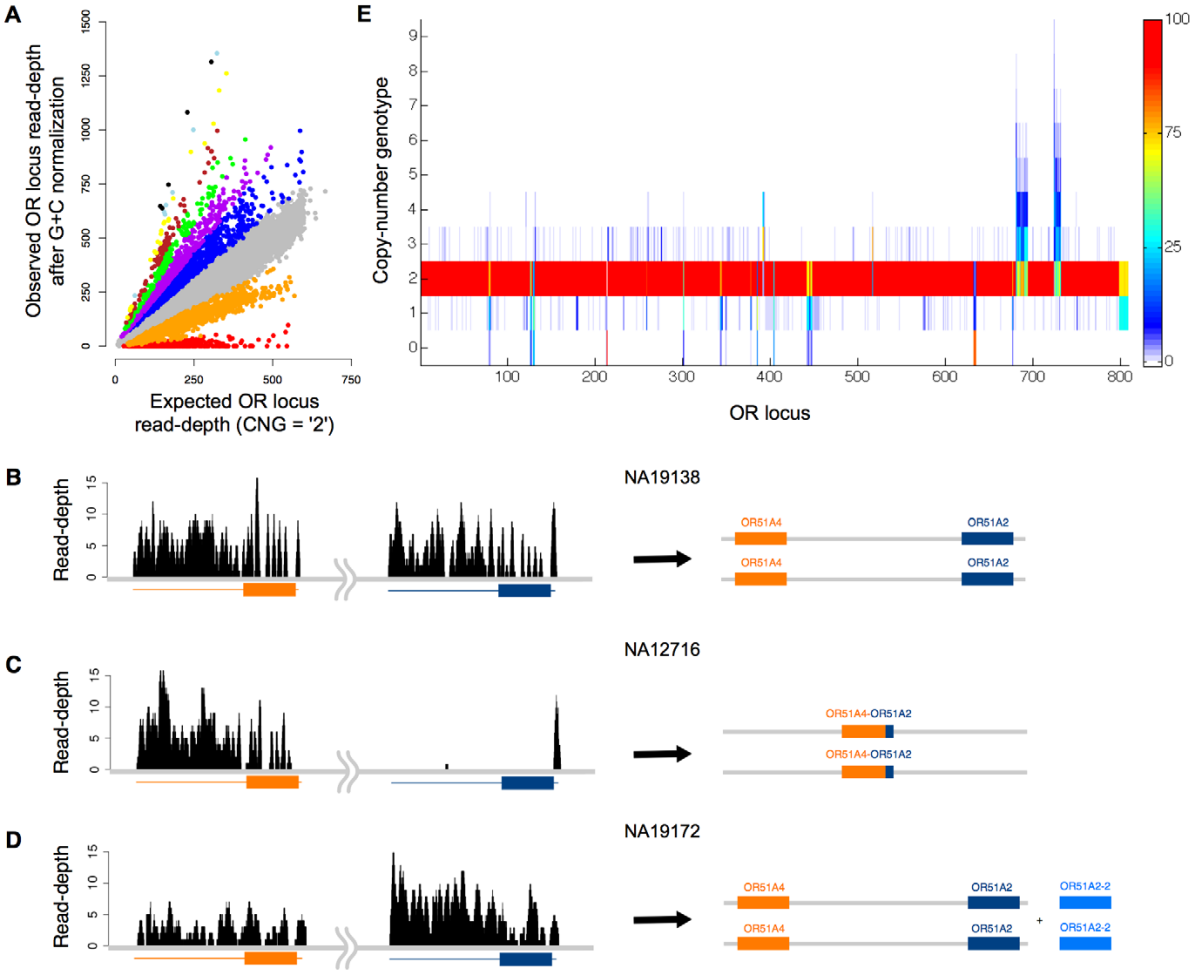
Copy-number genotype inference in olfactory receptor (OR) loci across 150 individuals. **A**. Distribution of locus-specific read-depth measurements in 808 OR loci. Altogether 121,200 data points are depicted (808 loci times 150 samples). Points relate the GC-adjusted read-depth to the *expected* read-depth, which is estimated based on the *k-mer* mappability of a locus and the genomic sequencing coverage of a sample. CopySeq copy-number genotypes are indicated by colors (bottom to top): ‘0’, red; ‘1’, orange; ‘2’, grey; ‘3’, blue; ‘4’, purple; ‘5’, green; ‘6’, brown; ‘7’, yellow; ‘8’, light blue; ‘9’, black). **B–D**. Dissecting a *complex* CNV region with CopySeq. The displayed region (chr11:4,921,968–4,930,581) harbors a multi-allelic CNV involving both a deletion and a duplication. The deletion results in an OR51A2—OR51A4 fusion-gene [4]. Read-depths are shown on the left and the inferred locus-structure on the right. CopySeq was carried out in conjunction with breakpoint-junction analysis [26], generating the following copy-number genotypes. NA19138: ‘2’ for OR51A4, ‘2’ for OR51A2, ‘0’ for the fusion-gene (**B**); NA12716: ‘0’, ‘0’, ‘2’ (**C**); NA19172: ‘2’, ‘4’, ‘0’ (**D**). Orange and blue boxes indicate open-reading frames (ORFs), and orange/blue lines denote the respective loci (with 3′ and 5′- regions). Both ORFs are on the reverse strand of the reference genome. The gene fusion occurred near the ORFs' 5′-end within a sequence stretch where both share extensive homology (thus, no reads map to this stretch uniquely). **E**. Copy-number genotype map of OR loci in 150 individuals. Each bar represents the frequency of a copy-number genotype (y-axis) at a particular OR locus (x-axis). Colors indicate copy-number genotype frequencies (color scheme is on the right).

We analyzed the OR loci as ∼3 kb regions encompassing the single-exon open reading frames (ORFs) and downstream as well as upstream sequence stretches (Materials and Methods). We assessed the mappability of reads onto OR loci and excluded only ∼5% (22 genes and 21 pseudogenes) from our analysis, as they displayed less than 500 mappable 36-mers per locus (Figure S1). Following read mapping we found that OR loci were covered on average by 209 (median = 190; see *e.g.*, Figure 3BCD) uniquely aligning reads per individual.

### Constructing a personalized locus copy-number map of ORs in 150 individuals

**Figure 4:**
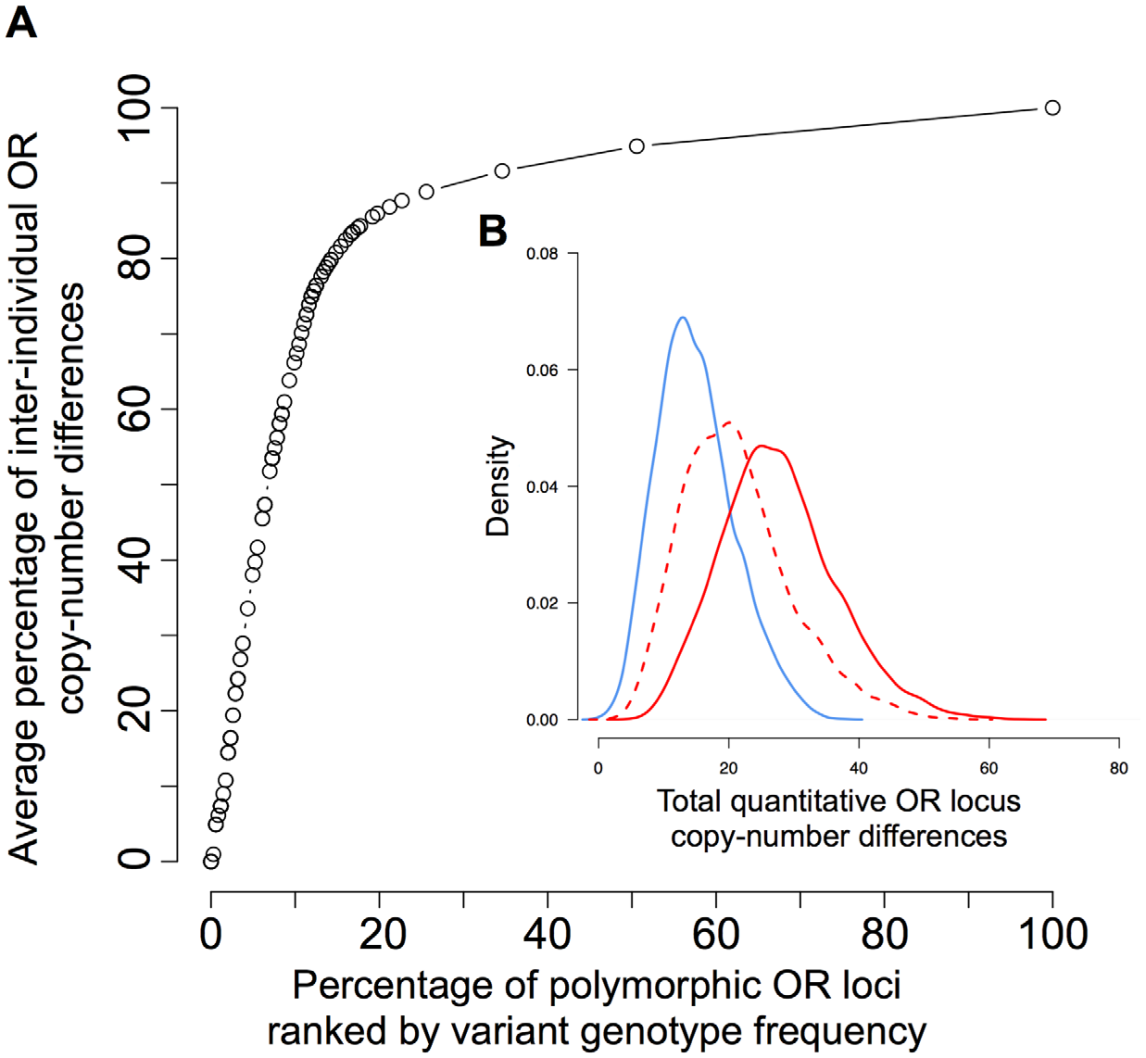
Distribution of inter-individual copy-number differences in autosomal OR loci. **A**. Commonly variable loci account for the majority of inter-individual OR copy number differences. OR loci were ranked by the frequency at which they displayed a copy-number genotype other than ‘2’ (indicating a CNV), followed by iterative exclusion of the rarest CNVs (*i.e.*, first the loci that most rarely vary in copy-number were excluded, then the more common ones). Pair-wise copy-number differences between all samples were calculated, and average copy-number differences across all pair-wise comparisons determined. The y-axis indicates the inter-individual copy-number difference as a percentage of the maximum average copy-number difference, and the x-axis indicates the percentage of all copy-number variable (polymorphic) OR loci for each OR frequency rank step. For example, ∼15% of the OR loci account for ∼80% of the inter-individual OR copy-number differences between any two samples. **B**. Distribution of inter-individual OR copy number differences computed separately for each pair of samples. Pair-wise copy-number differences were computed as quantitative differences between copy-number genotype values summed up over all OR loci between pairs of samples (x-axis). (In this regard, for example, the difference for a given locus is 2, if in one sample a copy-number genotype of ‘0’ and in the other a copy-number genotype of ‘2’ is inferred.). Blue solid line: OR genes; red solid line: OR pseudogenes; red dotted line: OR pseudogenes, excluding the CNV-enriched OR7E family.

We analyzed 808 mappable OR loci in 150 humans using CopySeq to construct a comprehensive and accurate OR locus copy-number map. Eleven of these loci are on chromosome X and thus naturally differ in copy-number between females and males. We thus focused in our analyses, described below, on CNVs in the 797 remaining autosomal regions, and made use of the eleven X-chromosomal regions for optimally setting the parameters of the method (*i.e.*, the Q-value; see Materials and Methods). Altogether, CopySeq inferred 4,573 loci with a copy-number different from the homozygous reference allele, which fell into 313 copy-number variable OR loci (Table S4). These involved 2,137 deletions (autosomal locus copies of ‘0’ to ‘1’) and 2,436 duplications (‘3’ and up to ‘9’ locus copies; Figure 3E). We excluded six out of the 313 autosomal copy-number variable OR regions, on the basis of previous reports that these loci, or their closest paralogs in the genome, likely represent extremely rare CNVs in the reference genome, or alternatively mis-assemblies [22] (these six loci displayed no, or very few, reference alleles). The remaining 307 loci were classified into 265 bi-allelic CNVs (*i.e.*, 135 bi-allelic deletions and 130 bi-allelic duplications) and 42 multi-allelic CNVs based on their inferred locus copy-numbers in 150 individuals (Materials and Methods). The fraction of variable OR genes and pseudogenes is 33% (130/387) and 38% (137/464), respectively. On average, we detected 25 copy-number variable OR loci per individual, *i.e.*, 8 OR genes (2.3%) and 17 OR pseudogenes (3.9%) (Figure S17). These correspond to, on average, 43 quantitative inter-individual copy-differences (Figure 4 and Table S11).

### Validating our OR gene copy-number map

**Figure 5:**
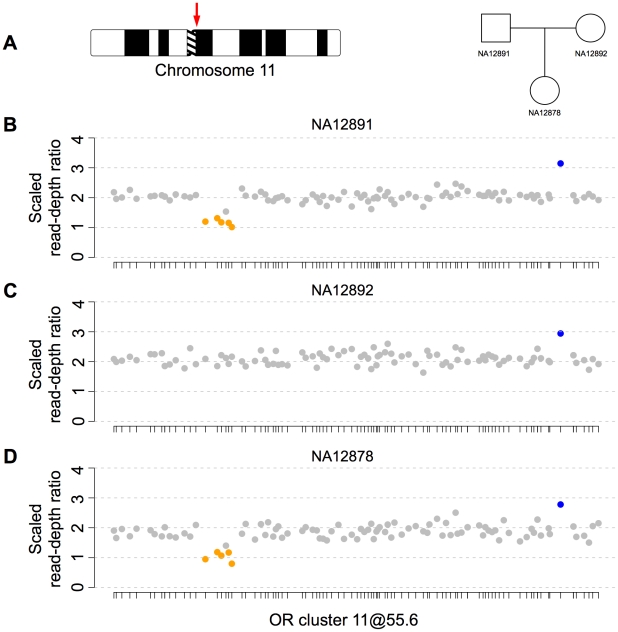
Heritability of CNVs in a parent-offspring trio of European ancestry. **A**. Chromosomal origin of the largest human OR genomic cluster and pedigree of the European family. **B–D**. CNV inheritance, indicated in terms of scaled read-depth ratios and inferred copy-number genotypes among 96 bi-allelic OR loci located in the largest human OR cluster (11@55.6; see nomenclature in http://genome.weizmann.ac.il/horde/; chr11:54,842,512–56,344,668). The x-axis represents genomic coordinates, and individual OR positions are marked by ticks. The copy-number genotypes identified in NA12891 (**B**), NA12892 (**C**), and NA12878 (**D**), were inferred based on low-coverage genomic data (Table S1) and are consistent with Mendelian segregation. Bi-allelic CNVs were classified according to copy-number genotypes identified in the European (CEU) individuals. Copy-number genotypes are color-coded: ‘1’, orange; ‘2’, grey; ‘3’, blue.

We next assessed the accuracy of CopySeq in OR loci using three distinct approaches. *First*, we examined a parent-offspring trio with European ancestry sequenced at low-coverage for the segregation of 772 OR loci that appeared bi-allelic, or displayed no CNV, across the examined European individuals (Materials and Methods). We found that all 16 copy-number genotypes inferred in the daughter, which included 7 heterozygous and 9 homozygous deletions, were consistent with Mendelian segregation (Figures 5, S10) suggesting high genotyping accuracy.

**Figure 6:**
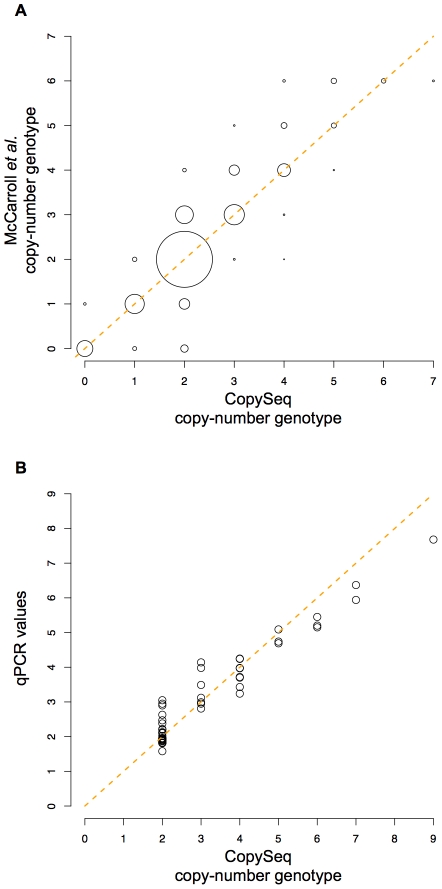
Concordance of copy-number genotypes inferred in OR loci with microarray-based calls and qPCR experiments. **A**. Comparison of >5,000 copy-number genotypes inferred in OR loci, using CopySeq, with microarray-based [14] copy-number genotypes. The comparison is based on 46 OR loci, assessed in 118 individuals. Circle size indicates the number of comparisons falling into a certain bin (the largest circle, representing >3,000 copy-number genotypes, corresponds to concordant copy-number genotype calls of the homozygous reference allele, *i.e.*, copy-number = ‘2’). Blue lines denote the function y = x and have been included to facilitate evaluation of the data. **B**. Validation of 50 copy-number genotypes in 5 OR loci×10 samples by qPCR. Experimentally determined qPCR values are expressed in terms of adjusted Ct values, which were estimated as described in the Materials and Methods section.

*Second*, we compared CopySeq with microarrays, *i.e.*, copy-number genotypes inferred with Affymetrix SNP 6.0 arrays [14] and Agilent CGH arrays [6] (Figures 6A, S11). The Affymetrix arrays ascertain ∼5% (46) of the autosomal 3 kb OR loci, allowing us to compare ∼5,400 copy-number genotypes in OR loci across the 118 overlapping samples. Indeed, copy-number genotypes reported in McCarroll *et al.* [14], ranging from 0 to 6, show a strong correlation with our genotype calls (Pearson correlation = 0.91; *P*<2.2e-16). We estimated a CNV false discovery rate of 1.7% (26/1561) and a sensitivity of 75% (1535/2061) for CopySeq (Materials and Methods), under the conservative assumption that the microarray-based calls [14] contain no false positives as well as no false negatives. Furthermore, under the same conservative assumption we estimated positive predictive values (or PPV) of 97% (683/704) for deletions and 99% (852/857) for duplications in the OR loci. We also compared our OR copy-number genotypes with genotypes inferred with Agilent CGH arrays [6] which ascertained ∼6% (51) of the OR loci, enabling us to compare >7,000 genotypes in the 149 overlapping samples. This comparison also revealed highly significant, albeit slightly weaker correlations (Pearson correlation 0.73; *P*<2.2e-16; Figure S11) – similar to the results we obtained for chromosome 1 CNVs.

*Third*, we used qPCR to obtain independent validation results for our copy-number genotypes in 10 individuals across five loci, by assessing 50 copy-number genotypes through experimental validation (Materials and Methods). Three of the five loci were randomly picked and two specifically selected since they displayed particular wide ranges in copy-number genotype (*i.e.*, up to ‘9’ copies). The loci further included regions that can reasonably be regarded as particularly hard to ascertain: *i.e.*, >90% of the nucleotides overlapped with SDs in four loci; four loci displayed multi-allelic CNVs; and ∼50% (24/50) of the assessed copy-number genotypes corresponded to duplications. Nonetheless, measured correlations with CopySeq's copy-number genotypes were excellent for the qPCR measurements (Pearson correlation = 0.96, *P*<2.2e-16; Table S13). Among these, we found that correlations were of high magnitude for CopySeq's genotypes including the extreme copy-number of ‘9’ (Figure 6B), which suggests that CopySeq enables generating copy-number genotypes accurately over a wide range of locus copy-numbers. Furthermore, we picked the four loci displaying the strongest discrepancies between CopySeq and microarray studies [6], [14] (*i.e.*, genotype concordance <30%) for further qPCR validation in eight individuals each; three of these four loci intersected with recently duplicated SDs. The qPCR results were consistent with CopySeq in three out of the four assessed loci (Figure S14, Table S14, and Text S1), suggesting that NGS-based genotypes are at least similarly accurate, and may possibly be more accurate, than array-based genotypes in such regions.

### Comprehensive analysis of the OR copy-number map

Having established the accuracy of CopySeq in OR loci we next performed a global analysis of our OR copy-number map. First, we related deletions and duplications to previously published CNVs, *i.e.*, to CNVs reported in the DGV (version from December 2009) and in a recent microarray-based analysis of OR loci [21] currently not included in DGV. We found that 199 of the identified 307 copy-number variable autosomal OR loci overlap with already published CNVs. 50 out of 52 commonly variable OR loci (*i.e.*, such with reference allele frequency <95%; Table S3; see Text S1) had previously been reported. The remaining 108 OR loci were previously not reported to vary in copy-number; this included 99/108 (92%) rare CNVs (allele frequency <1.0%). Obviously, future surveys examining larger numbers of individuals are likely to report further rare CNVs affecting ORs.

Our genotype frequency analysis further revealed several instances in which the majority of individuals displayed a non-reference allele. Given the importance of establishing a common and comprehensive OR repertoire for functional studies we analyzed these cases in detail, first by calculating CNV allele frequencies in all bi-allelic loci. This analysis suggested that in eight OR loci (including, for instance, OR2BH1P) the reference allele represented a minor allele (*i.e.*, reference allele frequency <50%); two of these loci involved genes (see below). Furthermore, we estimated reference allele frequencies in multi-allelic loci by assuming the presence of the homozygous reference allele if a locus copy-number of ‘2’ was inferred (see Text S1). This analysis revealed that in one multi-allelic OR pseudogene locus (OR11J2P) the reference sequence appears to represent a minor frequency allele (Table S3). Moreover, we estimated confidence intervals (95%) for reference allele frequencies and identified five additional loci (*e.g.*, OR4A45P) that are situated in transition between minor and major alleles, *i.e.*, with an alternative allele frequency close to 50% (see Text S1).

We next analyzed in further detail OR loci that displayed unusual (*i.e.*, non-reference) copy-number genotypes in the vast majority of samples. In particular, our results indicated that OR4C3 and OR4C5 genes as well as the OR4C4P pseudogene (all located in one genomic interval on chromosome 11) are duplicated in most (>95%) individuals (Table S3). We mined an alternative assembly of the human genome and found a close duplication (95% identity) of this genomic interval at another, distinct location on chromosome 11, suggesting that the reference genome version at the original interval may either be based on a rare deletion in the region (see Text S1), or may potentially represent a mis-assembly of the reference (as recently discussed in Young *et al.*, 2008). Whichever the case might be, absence of the common allele sequence from the reference genome and the high sequence identity between the duplicated segments, resulted in mapping of all reads originating from both loci onto one locus. Notably, we also identified a segment on chromosome 12 which contained three OR pseudogenes (OR7E140P, OR7E148P, OR7E149P) that were homozygously deleted in all European and Asian individuals, but were present in the Yoruba individuals with ∼36% allele frequency. The absence of orthologs of these pseudogenes in chimpanzee and orangutan suggests that they likely represent a recent human-specific insertion.

In addition, we assessed whether common, rather than rare CNVs are responsible for the majority of measured inter-individual OR copy-number differences. We thus ranked copy-number variable OR loci by the frequency at which they displayed an alternative (CNV) allele and recomputed the inter-individual OR copy-number differences. We found that a small number of relatively common variants affecting OR loci were responsible for most of the ascertained variation: *i.e.*, the ∼15% most commonly variable loci captured approximately ∼80% of the inter-individual copy-number differences, and the ∼50% most common loci captured ∼95% of the differences (Figure 4A). Thus, common CNVs lead to most inter-individual differences in OR copy-number and thus may have a relatively strong impact on variation in smell perception in humans; these common variants thus represent attractive candidate regions for future association studies.

We mostly analyzed CNVs in an OR locus-by-locus basis. It is evident from Figure 3E, however, that CNVs present at high frequency (*i.e.*, OR loci that frequently display CNGs other than ‘0’) tend to cluster, suggesting that they may form CNV hotspots or correspond to *large* CNVs spanning multiple loci [21], [22]. We thus assessed consecutive CNV calls in annotated genomic OR clusters, assuming that adjacent OR loci that are both involved in a duplication or are both involved in a deletion, respectively, may potentially be explained by a single *large* CNV. This analysis revealed that ∼36% of CNV events involve single-OR-locus CNVs, whereas the remaining (potentially *large*) CNVs may span at least two adjacent OR loci (Table S4).

Deletions are particularly likely to have an impact on smell perception, as they may abrogate OR function. In our set, we found that 14.5% (56/387) of the OR genes harbored at least one deletion allele, and in 5.9% (23/387) of the OR loci, deletions were observed with an allele frequency >1.0%. Homozygous deletions are of particular interest due to their potential phenotypic effects. We found that these are widespread with 25% of the analyzed individuals displaying at least one homozygous OR gene deletion and some individuals displaying up to four such ‘holes’ in their functional OR content. To obtain an inclusive list of alleles responsible for holes in the human OR repertoire we also mined the 150 individuals for SNPs associated with OR gene inactivation (*i.e.*, those causing segregating pseudogenes [29]) and identified 24 previously known and 49 novel SNPs resulting in altered OR gene start and stop codons (Materials and Methods and Table S12). The list of inactivating genetic variants, including locus deletions and segregating pseudogenes, covers ∼15% and ∼20% of the OR gene repertoire, respectively. These genetic variants represent excellent candidates for future association studies on olfaction.

### Studying genomic biases with regard to CNVs in OR loci

We next used CopySeq to obtain insights into how region-specific genomic biases may have shaped the genomic distribution of CNVs affecting ORs. First, we compared the relative CNV abundance between OR pseudogenes and OR genes. In this regard, both random drift [20] and selective constraints [21] have been implicated in influencing the distribution of CNVs in OR genes and pseudogenes. Our analysis of copy-number genotypes indicated that pseudogenes generally show more variance in locus copy-number than genes (Figure 4B). Selective constraints can be assessed by examining deletions, *i.e.*, the removal of functional genes, as deletions are more often deleterious than duplications and thus are more biased away from functionally relevant genomic regions ([1] and references therein). We found that on average less genes (3.8 per individual, *i.e.*, 1.0% of the OR gene set) than pseudogenes (9.5 per individual, *i.e.*, 2.0%) were deleted per individual, a trend that was significant (2-fold relative depletion; *P* = 0.009 based on a permutation test; see Text S1). Furthermore, 0.4 genes (0.1% of the OR gene set) and 3.9 pseudogenes (0.8% of the set) were homozygously deleted in each individual (8-fold relative depletion; *P* = 0.004, permutation test). Both trends persisted, but lost significance when excluding the 7E subfamily, a rapidly evolving OR subfamily with 85 members [30] (see Text S1). Thus, while selective constraints acting on OR genes may in part explain the distribution of CNVs in OR genes and pseudogenes, these constraints are not extensive for the OR family, and formational biases presumably contributed to the observed genomic distribution of CNVs affecting ORs (for example, we note that the proportion of multi-allelic loci among CNV loci is similar between OR gene and OR pseudogene loci, *i.e.*, in each case about 5%).

In contrast to previous surveys, CopySeq enabled us to comprehensively assess whether multi-allelic CNVs and bi-allelic CNVs affect OR loci in different sequence contexts. In particular, we assessed to what extent bi-allelic and multi-allelic CNVs occur in SDs. The enrichment of copy-variable ORs in SDs (90/307) was significantly higher compared to non-variable ORs (86/464) (∼1.6-fold; *P*<1e-4, permutation test). Our results revealed that particularly multi-allelic OR loci were strongly enriched in SDs (3.5-fold over non-variable OR loci; *P* = 0, chi-square = 44.9, chi-square test; Figure S12). Furthermore, we observed a 4.3-fold enrichment for loci displaying high copy-number genotypes (‘5’ and more copies; Figure S15) and a 2.7-fold enrichment for loci displaying both deletions and duplications (Figure S12). This association is possibly due to the predisposition of regions rich in SDs to show recurrent CNV formation by non-allelic homologous recombination [15].

**Figure 7:**
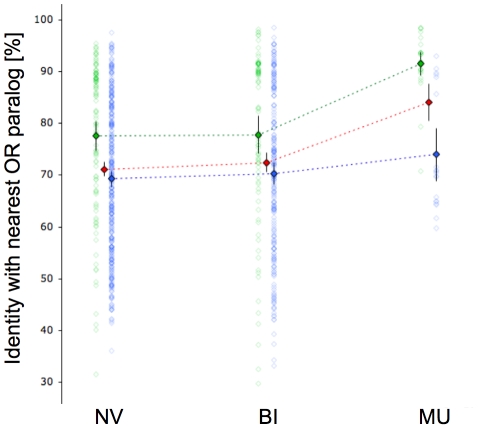
Analysis of ‘young’ and ‘ancient’ ORs. The figure displays the distribution of sequence identities with the most similar (‘nearest’) paralog for non-variable, bi-allelic, and multi-allelic OR loci. Each point represents the sequence identity of an OR to its nearest paralog (y-axis), and the type of locus (non-variable, NV; bi-allelic, BI; multi-allelic, MU). Green points: OR locus lacks a one-to-one ortholog in the chimpanzee genome; blue points: OR locus has a one-to-one ortholog in the chimpanzee genome (as assessed by comparing human and chimpanzee ORFs at the DNA level using BLAST, and classifying as one-to-one orthologs sequences displaying mutually highest sequence identity). Blue and green rhomboids represent the corresponding distribution average; red rhomboids represent averages for NV, BI, and MU. Rhomboid error bars represent 95% confidence intervals of the average.

In addition, CopySeq enabled us to dissect the contribution of evolutionarily *young* and more *ancient* ORs to copy-number variation in OR loci. It was reported that *young* ORs (some of which correspond to SDs) are particularly prone to be affected by CNVs, with *young* loci defined both based on the presence of paralogs sharing high sequence identity and based on the lack of one-to-one orthologs in the chimpanzee [21]. Our analysis revealed that *young* ORs affected by CNVs mainly lie in multi-allelic loci. As shown in Figure 7 multi-allelic loci displayed a significant enrichment for ORs with high sequence identity paralogs compared to both bi-allelic loci and non-variable loci (*i.e.*, the average sequence identity to the closest paralog was 84.5% in the multi-allelic loci and ≤73% in both the non-variable or bi-allelic loci, respectively; the differences are significant with *P*<0.0001; t-test). Furthermore, multi-allelic ORs displayed a >2-fold enrichment for ORs lacking a one-to-one ortholog in chimp compared to each other group (the differences were significant with *P*<0.0001; Chi-square test). Possible explanations for the differences include selective constraints and formational biases, both of which likely vary among different genomic regions.

### Population-specific aspects of OR copy-number variation

**Figure 8:**
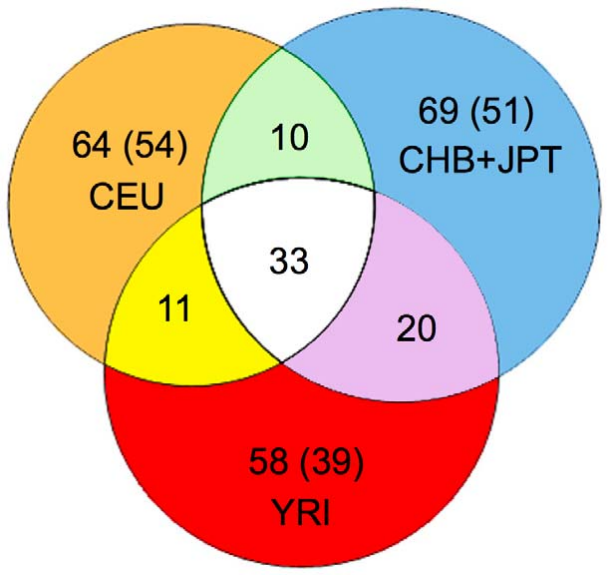
Analysis of the population distribution of bi-allelic OR loci reveals shared and population-specific CNVs. Venn diagram of 265 bi-allelic OR loci, which were distributed according to their recorded presence in the three populations analyzed (CEU, CHB+JPT, and YRI). Numbers in parentheses indicate OR loci in which a single copy-number genotype other than ‘2’ (indicating a CNV) was observed across 150 individuals; these loci may display rare, rather than population-specific CNVs.

We next assessed whether CNVs affecting OR loci display differences among individuals from diverse ancestries. Even when excluding rare CNVs that were identified only once amongst all individuals we observed 19 CNVs only in the analyzed Africans, 18 only in the Asians, and 10 only in the Europeans (Figure 8A). Furthermore, we carried out principal component analysis (PCA) of the copy-number genotypes generated across all 265 bi-allelic autosomal CNV loci. The PCA yielded a visible separation of the African group from the combined group of Europeans and Asians by the first two principal components (Figure S13A), and a separation of all three ethnic groups when analyzing the second and third component (Figure S13B). Note that the better distinction of Africans from European and Asian groups is in line with the well documented bottleneck effect, as evident from multiple large scale SNP studies [27]. When examining the failure of the first component to separate the three ethnic groups we identified a common bi-allelic deletion spanning three OR genes (OR4C11, OR4P4, OR4S2) and two OR pseudogenes, which drove the separation into three visible clusters by the first component (Figure S13). The three clusters represent the average OR locus copy-number genotype, with the left cluster representing the homozygous reference allele, the central cluster the heterozygous deletion, and the right cluster the homozygous deletion, respectively. The PCA and further analysis showed that all African individuals analyzed in our survey have at least one copy of the allele, whereas 7–10% of Europeans and Asians have all three functional OR genes homozygously deleted. In this regard, for example, a deletion, which encompassed the OR52E8 gene, was observed with appreciable allele-frequency (18%) in the Africans, whereas the allele was not observed in the other populations. Overall our PCA analysis of bi-allelic loci reflects findings from previously published SNP results [27], even though we used only 265 bi-allelic loci in our PCA analysis as opposed to hundreds of thousands of SNPs. Lastly, CopySeq enabled us to examine population differences in the distribution of bi-allelic and multi-allelic OR loci: indeed, in at least 11 OR loci CNVs were observed as multi-allelic in one population and bi-allelic in another (see Text S1).

### Dissecting a multi-allelic OR CNV region

We reasoned that the spatial resolution of NGS data may enable us to further dissect complex multi-allelic OR loci, *i.e.*, loci in which different CNV alleles coincide in the same genomic segment. Dissecting multi-allelic loci represents a crucial step to inform future association studies that examine the functional impact of each CNV allele separately. As a proof-of-principle we applied CNV-boundary redefinition to analyze a genomic interval containing the adjacent genes OR51A4 and OR51A2, which in some individuals form a fusion gene [4] (Figure 3BCD). In particular, since the sequenced breakpoints [4] of the deletion leading to the gene fusion fall into the respective OR coding regions, we inferred copy-number genotypes with CNV-boundary redefinition based on breakpoint-junction analysis (Figure 3BCD). Our analysis with CopySeq revealed that while the deletion is a variant with ∼32% allele frequency, an additional duplication comprising only the OR51A2 gene is also frequently present, *i.e.*, was genotyped in 6 individuals (Table S4). Thus, applying CopySeq with CNV-boundary redefinition can help facilitate the dissection of multi-allelic CNV loci.

## Discussion

We have developed a computational approach, CopySeq, that discovers copy-number variable loci and subsequently assesses their locus copy-number in NGS data, using a rationale based on formal hypothesis testing. As such, CopySeq may facilitate analyzing CNVs in NGS-based genome–wide association studies. Our analyses revealed an excellent concordance of CopySeq with microarray platforms, qPCR experiments, and FISH experiments, suggesting high genotyping accuracy. We note that one possible source for discrepancies between array-CGH and CopySeq in CNVs intersecting with SDs might be the heuristic transformation of microarray intensity data in SDs into genotypes by population-wise clustering [6]. CopySeq does not apply population-wise clustering nor do its calls depend on comparing read-depths with reference samples for normalization. This makes CopySeq particularly suitable for genotyping CNVs in single individuals or for genotyping rare alleles (*i.e.*, cases where too few data exist for population-wise clustering).

While arrays are presently widely applied for CNV analysis [6], [14], [31] we foresee that in the near future with the completion of the 1000GP and other large-scale NGS projects there will be more genomes sequenced than such for which comprehensive array-based genotyping data will be available. Consequently, we anticipate that in the future, NGS-based genotyping of CNVs is likely to be widely applied. NGS data are generated in a genome-wide fashion and sequencing data can be re-interpreted without requiring experimental re-design to enable accurate copy-number genotyping, once new high-confidence CNV sets are becoming available (*e.g.*, following the assembly of new sequence insertions [32]). We expect that in the future, CopySeq will be applied along with CNV discovery approaches such as paired-end mapping [4] to combine the advantages of copy-number genotype ascertainment with accurate CNV discovery and CNV-boundary redefinition.

Furthermore, we demonstrated that CopySeq accurately infers copy-number genotypes in SD regions (regions known to be hard to ascertain for genetic variants), *i.e.*, in the OR gene family that is rich in highly identical paralogs. Our analysis of 150 individuals afforded the first comprehensive ascertainment of locus copy-numbers in the OR gene family. We found that more than a third of the human reference OR repertoire varies in copy-number across individuals and described many novel CNVs. Our first comprehensive report of copy-number genotypes in these regions provides a valuable resource for the community, since genotypes are an important prerequisite in associating CNVs with odor perception. While previous reports demonstrated an enrichment of variable ORs in SDs [22], our analysis revealed that multi-allelic and bi-allelic OR loci are differentially affected by SDs. In some cases, we furthermore observed distinct OR gene counts in different populations. We note that while many more individuals need to be genotyped before population-specificity of these variants can be confirmed, allele frequency differences are likely to contribute to population differences in smell-perception [33]. Also, while other studies hypothesized that OR genes and pseudogenes evolved in a neutral fashion by genomic drift [20], [22], our data suggest weak evolutionary pressures acting on OR genes (Figures 4B, S17).

We further observed an abundance of OR genes that are dysfunctional in a subset of the individuals analyzed. In this regard, OR deletion alleles and SNPs leading to gene pseudogenization are widespread, *i.e.*, about 15% and 20% of the functional OR repertoire harbor such variants, respectively. These inactivating variants represent attractive candidates for future association studies focusing on odorant perception [29], [34].

While CopySeq enables probabilistic copy-number genotyping in NGS data, it still has its limitations. One limitation of CopySeq is that it is confined to sequences already present in the reference genome – a limitation that will likely diminish soon, when more alternative human genome assemblies will become available. Furthermore, only unambiguously mappable sequences are considered by CopySeq. In this regard, ∼1% of the human genome is in very recently segmentally duplicated regions with >99.5% identity [35] – a fraction in which most short DNA reads will be non-unique. These regions are presently excluded by CopySeq. However, we reasonable expect that this limitation will diminish soon, as longer and more easily mappable reads (150 bp, or longer) are presently becoming the standard in NGS. In fact, in the upcoming main phase of the 1000GP human genomes will be sequenced mostly with paired-ends, with each end 100–150 bp in size or longer, which will facilitate the application of CopySeq in recently duplicated regions. Also, longer reads will enable the fine-mapping of CNV breakpoints and consequently will enable CNV-boundary redefinition (*e.g.*, by BJA) for a larger fraction of CNVs than is presently possible.

Very recently duplicated regions (>99.5% identity) can already be analyzed with Alkan *et al.*'s approach, which considers non-unique genomic mapping positions. Nevertheless, in non-SD regions we found that CopySeq displayed higher concordances than Alkan *et al.*'s approach with Affymetrix array-based locus copy-numbers (97.2% *vs.* 80.2%). Possible reasons for the improved concordance of CopySeq may be an increased accuracy of a statistical copy-number genotyping framework compared to depth-of-coverage analysis without a probabilistic genotyping model. In addition, CopySeq's genomic *k*-mer filtering scheme may have contributed to its improved concordance by removing read-depth specific noise originating from distant paralogs.

Finally, our inference and validation of genetic variants may guide the way to similar analyses for other difficult-to-ascertain CNV regions, such as the medically relevant [1] *CCL3L1*, β-defensin, and *FCGR* loci (see, for example, our analysis in the Text S1 with regard to the *FCGRB* locus on chromosome 1). Furthermore, CopySeq can be easily adapted to genome-wide scale analyses. As thousands of human genomes are becoming sequenced in the context of biomedical research studies (*e.g.*, cancer genomes or constitutional abnormalities), there is a strong need for accurate copy-number genotyping approaches operating on NGS data.

## Materials and Methods

### Acquisition and mapping of short-read data

Illumina sequencing data were obtained from the 1000 Genomes Project (1000GP; ftp://ftp.1000genomes.ebi.ac.uk/; July 2009 release). Those reads have been aligned against the reference genome (hg18; Build 36.1) with the MAQ [24] aligner (default parameters). The DNA reads were mostly sequenced as paired-end fragments with a read length of 36 nt. For each sample we recorded the total coverage of uniquely mapped reads (‘ends’), and kept unambiguous read-alignments onto the following regions: sets of previously defined CNVs on chromosome 1; a set of genomic regions comprising ∼1% of the reference genome that were analyzed to correct for the G+C content in a sample-specific manner (see below); a set of 5 Mb genomic intervals for variance model parameter estimation (see below); and all human OR loci (see below). The identification of unambiguous (unique) read alignments benefitted from the MAQ feature to infer unambiguous alignments even if only one end of a paired read aligns uniquely to the genome, by combining information from the mapped end and the paired-end insert size distribution [24]. Instances of duplicated fragments (*i.e.*, PCR artifacts of the NGS library) were removed during the read mapping process (using the rmdup function of the MAQ toolkit).

### Definition of chromosome 1 CNV test set

To assess the performance of CopySeq we obtained 100 CNV loci <50 kb from chromosome 1 for which copy-number genotype measurements based on microarrays were available [14]. Out of these one CNV locus was excluded due to low mappability with 36-mers (*i.e.*, less than 500 mappable 36-mer subsequences within the CNV locus). CNV sizes in the resulting set of 99 CNVs range from 1–49 kb with a median CNV size of 6.9 kb (mean = 11 kb).

### Definition of genomic regions for G+C-content normalization

The G+C correction step of CopySeq required the analysis of regions that ideally should be invariable with regard to locus copy-number. Therefore, we sampled 30 Mb in 10 kb bins (*i.e.*, ∼1% of the human reference genome) and excluded regions annotated as copy-number variable in the Database of Genomic Variants (DGV).

### Definition of genomic regions for variance model parameter estimation

We randomly sampled one hundred 50 kb loci from all autosomes that are invariable in copy-number as assessed by CNV entries in the DGV database (v9, March 2010). We further controlled that the number of sampled loci within an isochore family is proportional to the genome-wide amount of DNA in isochore families. To model the dependency of locus size and read-depth ratio variance within a locus class (*e.g.*, 1 kb or 5 kb), we generated in *total* 15 datasets by subdividing each 50 kb locus into non-overlapping segments of various length (*i.e.*, 1, 1.25, 1.5, 1.75, 2, 2.25, 2.5, 3, 5, 7.5, 10, 20, 30, and 40 kb).

### Definition of human olfactory receptor loci

We obtained the genomic coordinates of 851 annotated human olfactory receptor (OR) genes and pseudogenes from the HORDE database (Build 42; http://genome.weizmann.ac.il/horde/). CopySeq requires at least 500 bp of sequence to which reads can be mapped uniquely. ∼19% of the OR open reading frame (ORF) sequence display less than 500 bp of mappable sequence, explaining the necessity to extend OR loci by flanking sequences. OR loci included the ∼1 kb intron-less coding region as well as non-coding segments up- and downstream, *i.e.*, 100 bp of 5′-sequence and 2 kb of 3′-sequence. We regarded such ‘extension’ of the loci of interest to 3 kb as reasonable, since previously described [4], [21] CNVs affecting ORs were several kb in size. Indeed, mining CNVs by long insert size paired-end mapping [4] in an individual studied by the 1000GP (NA12878) confirmed that only very few CNVs (*i.e.*, three CNVs in NA12878 including the known OR51A2—OR51A4 fusion in Figure 3) harbor breakpoints in the OR territories. In the few cases where CNV breakpoints do fall into these territories, we recommend application of CopySeq with CNV-boundary redefinition. Throughout the manuscript the phrase “intact olfactory receptor ORFs” is used synonymously with “OR genes”, “genes”, or “OR gene repertoire”, whereas “disrupted olfactory receptor ORFs” are used synonymously with “OR pseudogenes” or “pseudogenes”.

### Construction of human genome mappability maps

We used Rozowsky *et al.*'s approach to generate mappability maps of the human reference genome using *k*-mer lengths of 36, 51, and 76nt, respectively [36]. The mappability maps contain information about the frequency of each genomic *k*-mer sub-sequence, *i.e.*, how many times the *k*-mer occurs exactly on the Watson and Crick strand in the reference genome. The 36, 51, and 76 *k*-mers account for the three different Illumina read length sizes that were used in the 1000GP. CopySeq infers copy-number genotypes by assessing reads aligned against the *mappable* part of the genome, defined as *k*-mers subsequences that result in a genome-wide *k*-mer frequency of one (*i.e.*, *k*-mer sequences that remapped against the reference exactly once).

### Estimation of locus read-depth ratios

Before inferring copy-number genotypes, CopySeq measures the *locus read-depth ratio* for each predefined locus in question. The *observed locus read-depth* is defined as the sum of reads from a sample that unambiguously map within the boundaries of the predefined locus. The *locus read-depth ratio* 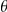 is defined as the ratio between the *observed locus read depth D* and the *expected locus read-depth E*, an estimate generated by evaluating the locus-specific G+C-content (see below), the mappability map, and the genome-wide sequencing coverage. In particular, for a predefined locus *i*, in individual *j*, and using the mappability map *k* (*i.e.*, *k*-mer size *k*) the expected locus read-depth *E_ijk_* for invariant locus *i* is estimated with 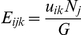, where *u_ik_* is the number of *k*-mers within locus *i* that are unique in the genome, *N_j_* is the number of uniquely aligned sequence reads against the reference genome in individual *j*, and *G* is the size of the genome (2,858,018,193 nucleotides in hg18, excluding the mitochondrial DNA).

In order to infer copy-number genotypes (copy-number genotypes) for locus *i* the read-depth ratio 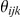 was calculated with 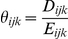, where *D_ijk_* is the observed locus read-depth (*i.e.*, number of reads mapped onto unique *k*-mer positions in the locus) and *E_ijk_* is the expected locus read-depth. Thus, for example, a ‘normal’ copy-number genotype (*e.g.*, copy-number genotype = ‘2’ in autosomal DNA, which may be considered as the ‘baseline’ for locus copy-number measurements) will result in a read-depth ratio 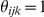, a copy-number genotype of ‘1’ (heterozygous deletion) will result in 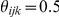, and a copy-number genotype of ‘3’ (heterozygous duplication) in 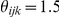 (see Figure S19).

### Locus G+C-content normalization

Earlier reports observed a correlation between Illumina sequence read coverage and G+C-content [9]. To correct for this confounding factor for read-depth analysis, CopySeq makes use of a set of 3,000 *normalization loci* (see above) to construct a G+C-normalization curve separately for each individual. This normalization curve is used for each locus to adjust its locus read-depth ratio according to the G+C content. Outlier loci (representing for example *de novo* CNVs) were identified by conservative criteria (*i.e.*, 1^st^-Quartile(read-depth ratio)-3*IQR(read-depth ratio) and 3^rd^-Quartile(read-depth ratio)+3*IQR(read-depth ratio)). For each normalization locus the G+C content of its mappable nucleotides was calculated and the sample-specific relationship between locus G+C content and locus read-depth ratio 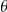 assessed. A sample-specific cubic smoothing spline function (using the R function *smooth.spline*) was fitted into the distribution of zero-centered data in order to model the underlying trend in the data by controlling for the smoothing parameter 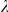 via the generalized cross-validation criteria. The spline fit was later on used as a normalization function to predict the read-depth ratio accounting for the locus G+C-content. The resulting fit explained about 67% of the Poisson variance over-dispersion (Figure S5) (*i.e.*, the *n*-fold variance as compared to the theoretical variance expected from random sequencing) and accounts for the observed reduced locus read-depth ratio at both sides of the G+C-distribution [25] (see Figure S2). Using the obtained fit we calculated the expected RDR 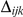 that is solely explained by the locus G+C content with the R function *predict.smooth.spline* and corrected the raw read-depth ratio with 
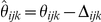
; as above, 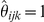 refers to normal locus copy-number, duplications result in 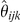 greater than 1 and deletions in 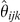 smaller than 1. Of note, after G+C-content normalization we observed a strong relation between sequencing coverage and read-depth variance (*i.e.*, decrease of variance with increase in coverage) that was not evident before normalization (see Figure S4). Based on the normalized read-depth ratio variance of the normalization loci and a cutoff value (0.01) we excluded 20 out of 170 initially assessed individuals from our analysis that were sequenced at low-coverage (<1×) (Figure S6).

### Copy-number genotyping: CNV identification

CopySeq initiates the copy-number genotyping step with a *CNV identification* module, in which two distinct hypotheses are tested at each locus: the null hypothesis *H_0_*: 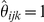 (*i.e.*, ‘normal’ locus copy-number) and the alternative hypothesis *H_1_*: 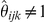 (*i.e.*, presence of a CNV). As values of 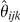 follow approximately a normal distribution in locus-copy number invariant *normalization loci* (Figure S3), we reasonably applied the *z*-statistic for assessing whether a given locus read-depth ratio deviates from the null hypothesis *H_0_* (*i.e.*, whether the read-depth ratio is unexpectedly high, or low, indicating a CNV). The alternative hypothesis *H_1_* is that 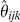 is drawn from a different distribution (duplication or deletion). Thus, the *z*-statistic for locus *i* in individual *j* was obtained with 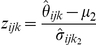, where 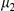 is the expected read-depth ratio given a normal (‘2’) locus-copy number, *i.e.*, 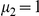, and 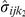 is the read-depth ratio standard deviation defined as 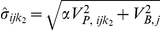, with 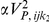 as the locus-length dependent read-depth ratio variance scaled by factor alpha (explained below) assuming an invariant locus and an additive read-depth ratio variance component 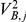 representing additional experimental *background noise* (see below). Each *z*-score *z_ijk_* was transformed into a two-sided p-value *p_ijk_* using the cumulative distribution function of the standard normal distribution 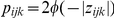. *P*-values were corrected for multiple testing, yielding Q-values, using Benjamini & Hochberg FDR correction. A global Q-value threshold was empirically determined in the following way. We estimated the CNV recall rate by calling *deletion* genotypes (*i.e.*, copy-number genotype = ‘1’) for 11 OR loci on chromosome X in 57 *male* samples, assuming that they display no CNVs (Figure S7). Using this setup, a Q-value threshold of 5% resulted in an inferred CNV recall rate of 96.5% for ∼3 kb loci, *i.e.*, in 605 out of 627 cases CopySeq predicted a *deletion*, as expected in the male samples. We furthermore estimated the CNV false discovery using the *female* samples, by conservatively assuming that all OR loci on chromosome X display a normal locus copy-number of ‘2’. Using this setup, in none of 1,023 cases CopySeq predicted ‘CNVs’, suggesting a very low CNV false-discovery rate.

### Copy-number genotyping: Locus copy-number inference

To generate genotype calls, CopySeq models copy-number genotypes as probability distributions accounting for locus-length, copy-number genotype, and sample-dependent noise. Specifically, copy-number genotype probability distributions are modeled as Gaussian distributions that incorporate both a Poisson variance term that depends on the read-depth of a locus, as well as an additional global (background) variance term that does not depend on locus length and copy-number genotype. The mean of each Gaussian is set according to expected values of the locus read-depth ratio, where the expected value for the locus read-depth ratio is 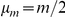, with *m* being a specific copy-number genotype among *c* possible copy-number genotypes *C_0_…C_c_*. For example, for *m* = 2 (no CNV) *μ*_2_ = 1; for *m* = 1 (heterozygous deletion) *μ*_1_ = 0.5; and for *m* = 3 (heterozygous duplication) *μ*_3_ = 1.5. These theoretical means are in excellent agreement with experimental data (see Figure S19). The probability density function for copy-number genotype *m* is calculated as:
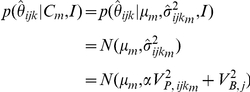
where 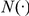 is the Normal probability density function, 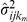 is our read-depth ratio variance estimate, 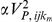 is the scaled locus-length- and copy-number genotype-dependent variance term, and 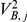 is an additive global *background noise* variance term (explained below). We calculated the locus-length- and copy-number genotype-dependent read-depth ratio variance 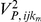 for copy-number genotype *m* as 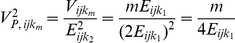, where 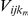 is the theoretical Poisson read-depth variance that increases linearly with copy-number genotype *m*, 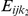 is the expected locus read-depth given an invariant locus copy-number of ‘2’, and 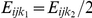 is the read-depth for a locus copy number ‘1’. The expression 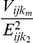 is also known as the *squared coefficient of variation* and can be viewed as the *scaled* variance of the Poisson distribution. The *a priori* knowledge about the model and parameters is summarized in the background information *I*. The most plausible copy-number genotype is inferred using classical Bayes' theorem:
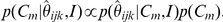
The prior density *p(C_m_)* is modeled as a uniform density function with *p(C_m_)* = 1/*c*, where *c* is the total number of possible copy-number genotype values. We reasonably chose to use a uniform prior as a neutral prior for classification on a genome-wide scale. Although we could foresee alternative approaches for estimating non-uniform priors, such as expectation maximization (EM), this would require extensive training data to reflect the underlying copy-number distribution for genomic locus of interest, including regions with high and low allele frequencies. Using Bayes' theorem, each locus was labeled with a copy-number genotype *m* that maximizes the posterior probability using the *maximum a posteriori* (MAP) estimate.

### Copy-number genotyping confidence score estimation

CopySeq calculates a confidence score (similar to a logarithm of odds, or LOD, score) for each locus, expressing the uncertainty of assigning the correct copy-number genotype, with
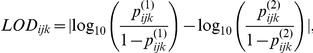
where 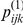 and 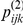 are estimated probabilities for the most plausible and the second most plausible copy-number genotype, respectively. A LOD score of 2, *e.g.*, means that the probability for the most plausible copy-number genotype is 100 (*i.e.*, 10^2^) times higher than for the next plausible copy-number genotype.

### Estimation of the non-locus dependent read-depth ratio variance 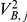 and scaling factor alpha 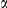


We observe that the theoretical minimal variance predicted by a Poisson sampling model is insufficient to explain the observed locus read-depth variance in loci of various length (see Figure S3D). We thus consider a variance model that approximates the observed locus length-dependent and length-independent variance in order to account for the over-dispersion. We assume that the read-depth ratios behave independently and are drawn as random samples from a normal distribution for loci with the same size. Normal quantile-quantile (Q-Q) plots of loci with different sizes (*e.g.*, 1 kb, 5 kb, and 10 kb) support the assumption of normality for read-depth ratios (see Figure S3ABC). We observe that the total read-depth ratio variance for loci of a specific length class follows a linear combination of a global (non-locus dependent) background variance term 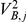 and a scaled length-dependent Poisson variance term 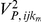, *i.e.*, 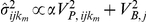 (see Figure S3E). The model parameters alpha and background variance were estimated via linear regression with the variance model dataset as described above. The average background variance among the 150 samples is approximately similar across samples (∼0.002) and the scaling factor alpha ranges between ∼1–4.

### CNV-boundary redefinition by paired-end mapping and breakpoint-junction analysis

CopySeq has the ability to use paired-end mapping [4] (PEM) and the recently published breakpoint-junction data [26] (BJA), *i.e.*, to redefine the boundaries (*i.e.*, breakpoints) of CNVs for copy-number genotyping on a subset of CNV loci (i.e., such for which PEM or BJA information are available). Thereby, CopySeq defines CNV-boundaries as previously described in publications on PEM and BJA. We assessed the utility of integrating PEM and BJA into CopySeq to enable CNV boundary-redefinition. Specifically, we focused on the chromosome 1 CNV set (see above). In particular, in the case of PEM we fine-mapped the breakpoints of CNVs using high-confidence (long-read and high-coverage) paired-end reads from the sample NA18505, which was sequenced at >8.5× physical coverage with 3 kb insert size paired-end reads generated with the 454 sequencing technology; the long reads generated by 454 sequencing allow for high-confidence placement of paired-ends [4]. Furthermore, the paired-end library insert size was ∼3 kb, a reasonable insert size for analyzing OR loci, which may recombine by non-allelic homologous recombination involving ∼900 bp ORFs [4], [21]. To initiate the CNV-boundary redefinition for CNV-loci of interest, we required a reciprocal overlap of >51% between microarray-based CNV coordinates of the chromosome 1 test set and the CNV-breakpoints as inferred by PEM. BJA was carried out using a library of ∼1,800 SVs with sequenced breakpoints [26], and applying the same overlap criteria (>51%) as for the PEM data. When applying PEM or BJA we reasonably assumed, based on previously published observations [4], [26], that in bi-allelic CNV loci breakpoints are identical across analyzed individuals; thus, CopySeq was able to use redefined CNV-boundaries in all individuals when assessing bi-allelic CNVs (note that BJA and PEM cannot be applied in the case of multi-allelic CNV loci, as CNV boundaries may differ in recurrent CNV formation events).

### Estimation of genotyping concordance with microarray-based studies

To assess the concordance of CopySeq-based copy-number genotypes with array-based copy-number genotypes we obtained data from two previous array-based surveys [6], [14]. The sample overlap between our study and the array-based studies is high with 118 (McCarroll *et al.*) and 149 (Conrad *et al.*), respectively. *Genotyping concordance* is defined as the number of copy-number genotypes that display *exactly identical* values (*e.g.*, copy-number genotype = ‘4’) between two studies, divided by the total number of copy-number genotypes that have been inferred. Each locus was tested in all individuals where data was available. In a small number of cases (see Tables S4 and S5, and data submitted with the array-based studies [6], [14]) no copy-number genotypes were inferred at a given confidence score threshold (‘NA’); these specific tests were obviously not considered when estimating genotyping concordance. When estimating genotyping concordance for OR loci, corresponding array-based CNVs [6], [14] were considered if they fully spanned the respective OR locus (*i.e.*, we required the OR locus to be fully contained in the respective CNV region previously assessed with arrays). We used standard terminology for statistical measures such as sensitivity, specificity, and precision rate (or positive predictive value, PPV; see *e.g.*, Table S6).

### Copy-number genotype validation by qPCR

Real-time quantitative PCR (qPCR) was carried out as described in [21]. Each experiment was carried out with 40 cycles and ended with a melting curve step to verify product specificity. Reactions with more than one peak in the melting curves were removed from further analysis. To address experimental variability due to primer differences and fluctuation in DNA concentration, we used a copy number-invariable gene (DSCR1) for normalization [21] and repeated each reaction at four DNA concentrations of 1, 2, 4, and 8 ng, in duplicates; the four concentrations allowed us to estimate the primer efficiency. Importantly, qPCR does not provide a measure of an absolute amount of material, but rather a comparison between samples. Thus, when analyzing a qPCR experiment we used copy-number genotypes inferred by CopySeq as an anchor point for calculations. Correlations were obtained from measuring copy-number differences across individuals at a given locus. Subsequent analyses were carried out as follows: (1) we calculated a matrix of pairwise Ct differences between all sample pairs for each OR and for DSCR1, at every DNA concentration separately. (2) To account for fluctuations in Ct arising from experimental procedures (*e.g.*, fluctuation in DNA concentrations or pipetting), we subtracted the DSCR1 based difference matrix from the OR difference matrix. We refer to the resulting matrix as the *corrected difference matrix*. (3) We combined data from all four concentrations by averaging the true pairwise Ct difference for each sample pair at every locus. (4) For each sample and locus, we used the predicted values from the other 9 samples as anchor, and thus computed 9 estimates for each genotype. For the transformation we used the experimentally estimated primer efficiency values. We then averaged the 9 values for each sample at each locus before calculating correlations. Primer sequences are listed in Tables S14, S20.

### Selection of OR loci for segregation analysis in an European parent-offspring trio

We followed the segregation pattern of OR copy-numbers in the parent offspring trio of European ancestry (NA12878, NA12891, and NA12892), by classifying 797 autosomal OR loci into 772 mono- and bi-allelic CNVs according to copy-number genotypes in CEU samples only. These 772 regions were analyzed to assess the segregation of copy-number genotype assignments.

### Analysis of segmental duplications

Coordinates for segmental duplications (SDs) were obtained from the UCSC genome browser (‘Segmental Dups’ track; hg18). Autosomal OR loci were classified as overlapping a SD if ≥51% of the ∼3 kb locus sequence overlapped.

### Permutation test for gene and pseudogene enrichment analysis

A permutation test was used to assess the significance of enrichment of certain copy-number genotypes among genes and pseudogenes. 1,000 permutations of possible values of a test statistic under random rearrangements of the gene and pseudogene labels were calculated to construct an exact (null) distribution. The test statistic was defined as the difference of the average number of predefined copy-number genotypes per group with two groups *A* and *B*, *i.e.*, 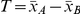, whereby group *A* is the collection of genes and group *B* is the collection of pseudogenes. The difference between the group means without permutation was calculated and referred to the observed value of the test statistic *t*. The permutation test was designed to determine whether the observed difference between the group means is large enough to reject the null hypothesis that the two groups have an identical probability distribution. To assess significance, a two-sided p-value of the test was calculated as the proportion of sampled permutations where the absolute difference of *T* was greater than or equal to the absolute value of *t*. The null hypothesis was rejected at a significance level *C* = 0.05.

### Principal component analysis with bi-allelic OR copy-number genotypes

We applied principal component analysis (PCA) on 150 individuals and on copy-number genotype data of 265 bi-allelic OR loci as implemented in the *R* function *prcomp* (www.r-project.org).

### SNP data mining

We extracted SNP data from the April 2009 SNP release of the 1000GP; a release encompassing SNP calls from 59 CHB+JPT, 56 YRI, and 56 CEU individuals, respectively. We identified all SNPs intersecting with OR coding regions and assessed their predicted effect on coding sequence using Perl scripts. Frequency and quality information relating to SNP data are available at the 1000GP website (http://www.1000genomes.org).

### CopySeq software and algorithm speed

CopySeq is implemented in Java and utilizes the SAM-SDK (http://picard.sourceforge.net/) for fast sequence alignment access. It can be obtained from http://embl.de/~korbel/copyseq/. CopySeq computes a typical genome-wide CNV dataset (with up to 30,000 CNV loci) in ∼1h.

## Supporting Information

Text S1 Supplementary Information for “Systematic Inference of Copy-Number Genotypes from Personal Genome Sequencing Data Reveals Extensive Olfactory Receptor Gene Content Diversity”.
(0.23 MB DOC)

Figure S1 Mappability of DNA sequences in OR loci. OR loci, regions with a median length of 3kb (including upstream and downstream regions), are sorted based on the number of uniquely mappable k-mers (i.e., one exact occurrence in the reference genome, build 36.1) per OR locus, with k = 36nt. Values on the y-axis, referring to the number of unique k-mers per locus, are expressed as starts (i.e., start sites) of 36-mer reads mapped onto the locus. Values on the x-axis are expressed in terms of the fraction of OR loci (in percent). For example, ∼95% of the total set of 851 OR loci in the reference genome have at least ∼500 mappable 36-mers (indicated by dashed line), and were thus considered in our study.
(0.20 MB TIF)

Figure S2 Sample-specific G+C-content bias normalization. Dots indicate the G+C content (or ‘GC content’, calculated as G+C/(A+C+T+G)) and raw read-depth ratios (i.e., #expected/#observed reads) measured in a set of normalization loci (i.e., copy-number invariable loci; see Materials and Methods). G+C bias was corrected by analyzing 3,000 10kb loci (i.e., ∼1% of the human genome) in each sample. Although read-depth ratios are expected to be evenly distributed in the genome, the panels A and B show a sample-specific bias in the read-depth of these normalization loci that appears associated with the G+C content; examples displayed correspond to NA10851 (A) and NA19238 (B). For each sample, G+C characteristics were fitted using a smoothing spline-based model that accounts for the non-uniform G+C behavior (red line). The G+C model fit served as a normalization curve for all loci assessed in a given sample.
(0.25 MB TIF)

Figure S3 Normality assumption for G+C content-adjusted read-depth ratios and read-depth ratio variance model fit. A–C. The figures display histograms (top) and normal Q-Q plots (bottom) of G+C content-adjusted read-depth ratios of three locus size classes (1 kb, 5 kb, and 10 kb; left to right) in NA10851. The red line, overlaid onto the histograms, displays a normal distribution fit to the histograms. The red line in the Q-Q plots implies that the adjusted read-depth ratio data are approximately normally distributed. D. The figure displays the dependency between the expected Poisson sampling read-depth ratio variance (x-axis) and the observed G+C-adjusted read-depth ratio variance (y-axis) for loci of different size classes (i.e., 1 kb, 1.25 kb, 1.5 kb, 1.75 kb, 2 kb, 2.25 kb, 2.5 kb, 3 kb, 5 kb, 7.5 kb, 10 kb, 20 kb, 30 kb, 40 kb, and 50 kb), measured with constant copy-number (‘2’) in NA10851. The red line represents a linear regression fit to the variance data (see Materials and Methods). E. This figure shows the G+C content-adjusted read-depth ratio variance (y-axis) for different classes of locus length (x-axes; same locus sizes as in (D)) in NA10851. The red line is the fit of the CopySeq variance model to the data (see Materials and Methods). The dashed horizontal line is the estimated copy-number and locus-length independent background variance term.
(0.31 MB TIF)

Figure S4 Variance of G+C normalization locus read-depth ratios. The figure relates the variance in read-depth ratio measurements to the genomic sequencing coverage before (A) and after normalization for G+C content (B). Altogether, 170 samples (i.e., including such excluded from further analysis; see Figure S6) were binned according to sequencing coverage. Box plots represent inter-quartile intervals for each bin (25%, 50%, and 75%), and the thick lines the respective median. Altogether, 20 samples were excluded due to increased variance, most of which displayed a very low sequencing coverage (i.e., <1-fold haploid coverage).
(0.25 MB TIF)

Figure S5 Poisson variance over-dispersion before and after G+C-content normalization. Boxplot of Poisson variance over-dispersion as assessed by control regions in 170 samples before (‘Pre’) and after (‘Post’) G+C content bias correction. Over-dispersion is defined as the n-fold variance as compared to the theoretical variance expected from random sequencing (i.e., assuming a Poisson sampling process). The average variance over-dispersion drops from 10.5-fold to 3.5-fold after G+C-content normalization.
(0.15 MB TIF)

Figure S6 Sample exclusion based on read-depth ratio variance. Samples are sorted on the x-axis by the G+C adjusted variance in the read depth ratio, recorded in the normalization loci (see Materials and Methods and Figure S4). The red line indicates the variance cutoff that was used to exclude samples (0.01); 150 samples were further analyzed in this study.
(0.19 MB TIF)

Figure S7 False discovery rate (FDR) threshold in the initial CNV identification step. The figure displays the recall rate for 11 OR loci on the X-chromosome, which were assessed in 57 male samples as a function of different Q-value (FDR) cutoffs in CopySeq's CNV identification step (see Materials and Methods). The recall rate was calculated, assuming that OR loci on chromosome X show no copy-number variation, i.e., all 627 copy-number genotypes in males are expected to display a copy-number genotype of ‘1’ (whereas copy-number genotypes of ‘2’ are expected in female samples); this assumption makes the X-chromosomal OR loci a reasonable test set for identifying a suitable Q-value threshold. For example, when applying a Q-value threshold of 5%, 96% of all OR loci in male samples were correctly identified in the CNV identification step and assigned a copy-number genotype of ‘1’. Furthermore, CopySeq identified a copy-number genotype of ‘2’ in all loci in 93 female samples.
(0.34 MB TIF)

Figure S8 Read-depth distribution in benchmark chromosome 1 CNV set and 150 individuals. Distribution of locus-specific read-depth measurements in the 99 chromosome 1 test loci and 150 individuals. As in Figure 3, the displayed points relate the G+C-adjusted read-depth to the expected read-depth (i.e., copy-number genotype = ‘2’), which is estimated based on the k-mer mappability of a locus and the genomic sequencing coverage of a sample. CopySeq copy-number genotypes are indicated by colors (bottom to top): ‘0’, red; ‘1’, orange; ‘2’, grey; ‘3’, blue; ‘4’, purple; ‘5’, green).
(0.31 MB TIF)

Figure S9 Copy-number genotypes in 150 individuals recorded in the chromosome 1 test set. Copy-number genotyping results for chromosome 1 example CNVs across 150 individuals (see Figure 2 for other examples). Copy-number genotypes inferred by CopySeq are displayed using the same color code as in Figure 2. Samples are sorted by ancestry (square, CEU; triangle, CHB+JPT; circle, YRI). A. chr1:10,293,128–10,300,570; bi-allelic deletion specific for samples with ancestry from Nigeria (YRI). B. chr1:61,886,594–61,890,775; bi-allelic deletion only observed in the CEU and CHB+JPT. C. chr1:235,190,798–235,198,134; bi-allelic duplication observed only in samples of Nigerian ancestry. D. chr1:755,964–799,636; multi-allelic CNV locus, only observed in CHB+JPT samples.
(0.25 MB TIF)

Figure S10 Heritability of locus copy-numbers in all 808 analyzed OR loci. Scaled read-depth and inferred copy-number genotypes of all 808 OR loci analyzed in a CEU parent-offspring trio (from top to bottom: daughter, NA12878; father, NA12891; mother, NA12892). OR loci are sorted according to chromosome and chromosomal coordinate (from left to right). Inferred copy-number genotypes are displayed with the same color code as in Figures 2, 3, 4. Several OR loci of interest are highlighted. Figure 4 displays, in part, the same data, but owing to space limitations focuses on the largest OR cluster, i.e., a cluster of OR genes and pseudogenes on chromosome 11.
(0.46 MB TIF)

Figure S11 Concordance of CopySeq copy-number genotypes and copy-number genotypes from the microarray-based study by Conrad et al. Comparison of >7,000 CopySeq-based locus copy-number assignments in OR loci with microarray-based copy-number genotypes from Conrad and co-workers [1]. The comparison is based on 51 OR loci, assessed in 149 individuals. Circle size indicates the number of comparisons falling into a certain bin (the largest circle, representing >5,000 copy-number assignments, corresponds to concordant copy-number genotype calls of ‘2’). The orange line denote the function y = x and has been included to facilitate evaluation of the data. The Pearson correlation between the Conrad et al. calls (made with an Agilent oligonucleotide array-CGH platform) and the CopySeq calls was 0.73, slightly weaker than the correlation we measured between CopySeq and Affymetrix SNP 6.0 arrays. This may be due to a higher accuracy of the Affymetrix arrays [2], e.g., owing to the sophisticated probe selection procedure employed; alternatively this may be due to an enrichment of particularly hard to ascertain OR loci (which may display relatively low correlations) in the Conrad et al. study.
(0.19 MB TIF)

Figure S12 Enrichment of segmental duplications in bi-allelic deletion and multi-allelic OR loci. Fraction of OR loci intersecting annotated SDs (i.e., assessed by a > = 51% overlap of the OR locus with SDs, based on the UCSC genome browser track ‘Segmental Dups’. OR loci were divided into five different classes: NV, non-variable (n = 501); DUP, bi-allelic duplications (n = 130); DEL, bi-allelic deletions (n = 135); DELDUP, multi-allelic OR loci displaying both deletion and duplications (n = 21); MULTDUP, multi-allelic loci that presumably underlie multiple duplications (i.e., loci with at least one copy-number genotype of ‘5’ or higher; n = 21). Red line indicate the average fraction of analyzed autosomal OR loci intersecting with SDs (i.e., 22%).
(0.20 MB TIF)

Figure S13 Principal component analysis (PCA) on 265 bi-allelic OR loci and 150 individuals. Population-specific copy-number variation of OR loci displayed by principle component analysis (PCA). PCA analysis was carried out on 265 bi-allelic OR loci in 150 individuals explaining 39% of the total variance (PC1 = 18.9%; PC2 = 12.6%; PC3 = 7.5%). Individuals are colored by ethnic group (YRI, red; CEU, orange; CHB+JPT, blue). A. The first component separates the African (YRI) population from the non-African populations (CEU, CHB, JPT). The three large clusters (PC1: ∼0, ∼1, ∼3) correspond to the copy-number genotypes ‘2’, ‘1’, and ‘0’ (from left to right) of a large, common deletion on chromosome 11 (encompassing OR4C11, OR4P4, OR4S2, OR4V1P, and OR4P1P). B. The second and third principal components facilitate a limited separation between the three populations (highlighted by ellipses), which may be explained by population-specific allele frequencies of a small number of CNVs.
(0.24 MB TIF)

Figure S14 qPCR experiments in OR loci with discordant copy-number genotypes. Four OR loci displaying the least concordance between CopySeq and microarray-based copy-number genotypes (x-axis) were tested by qPCR (y-axis) (Table S14). Each qPCR experiment used triplicates and was repeated at least twice when two primer pairs were used for each locus and three times for loci analysed with one specific primer pair (Detailed Ct, avg delta-Ct and stdev delta-Ct values for all experiments are summarized in Table S14). One representative experiment is shown where experimental qPCR values are compared to copy-number genotype predictions from CopySeq (red), Conrad et al. (blue) or McCarroll et al. (green). The following eight samples were assessed to enable evaluating discrepant calls: NA11881, NA11920, NA11994, NA11995, NA12003, NA12045, NA12155, and NA12716. A. OR4A45P: chr11:48,557,464–48,560,479. Missing data points: CopySeq, none; Conrad et al., none. The absence of a signal (Ct of 40) in qPCR results confirms the copy-number genotype of 0 inferred by CopySeq rather than the copy-number genotype of ‘2’ inferred by Conrad et al. Also, both primer pairs for this locus show the same results and confirm the conclusion made. B. OR11K1P: chr15:19,818,218–19,821,246. Missing data points: CopySeq, none; Conrad et al., NA12003; McCarroll et al., NA11920, NA12045, NA12716. The relative difference of up to 1.55 delta-Ct between DNAs (stdev of delta-Ct between DNAs over all three experiments≤0.09 Ct) and the clustering into several subgroups suggests a multi-allelic CNV, as predicted by CopySeq and McCarroll et al., rather than the absence of a CNV, as predicted by Conrad et al. C. OR5H5P: chr3:99,398,645–99,401,669. Missing data points: CopySeq, none; McCarroll et al., NA11920, NA12045. The almost identical Ct values (maximum difference of 0.23 delta-Ct) in the normal Ct range between all DNAs suggests absence of a CNV, as called by CopySeq, rather than the existence of a homozygous or heterozygous deletion, as inferred by McCarroll et al. (the latter would have resulted in a clear difference in qPCR values, as observed in (A)). D. OR4N4: chr15:19,883,737–19,886,784. Missing data points: CopySeq, none; McCarroll et al., NA11920, NA12045, NA12716. Clear Ct differences between samples support the multi-allelic copy-number genotype calls from both CopySeq and McCarroll et al. for this locus. However, missing data points for McCarroll et al. does not enable us to draw a final conclusion as to which copy-number genotype calls are in better agreement with the qPCR results.
(0.45 MB TIF)

Figure S15 Enrichment of segmental duplications in high copy-number genotype calls. Fraction of 118,650 (791 autosomal loci times 150 samples) OR copy-number genotype calls intersecting annotated SDs (i.e., assessed by requiring a > = 51% overlap of the OR locus with SDs, based on the UCSC genome browser track ‘Segmental Dups’)
(0.24 MB TIF)

Figure S16 Effect of sequencing coverage on genotyping error rate. Ten different coverages were generated by down-sampling DNA reads from the published NA18507 genome [7]. The following sequencing coverages were used: 0.5×, 1×, 2×, 3×, 4×, 5×, 10×, 20×, 30×, and 40×. CopySeq was applied on these read sets using default parameters (with requested LOD score >0). We compared CopySeq calls to two genome-wide genotype sets for NA18507, i.e., the sets published by McCarroll et al. [2] (A) and Conrad et al. [1] (B). CNV loci intersecting SDs were excluded to circumvent differences in the interpretation of locus-specific copy numbers between platforms in these regions. Genotyping error rates were calculated as #false genotypes/(#true genotypes+#false genotypes).
(0.18 MB TIF)

Figure S17 Distribution of variable OR loci within 150 individuals. The figure shows the percentage distribution of variable OR loci per individual relative to the reference genome. Blue solid line: OR genes; red solid line: OR pseudogenes; red dotted line: OR pseudogenes, excluding the CNV-enriched OR7E subfamily.
(0.22 MB TIF)

Figure S18 Locus-specific genotyping concordance for 500 benchmark CNVs on chromosome 1. The plots show the locus-specific genotyping concordance for 99 CNV loci on chromosome 1 taken from McCarroll et al. (A) and 401 CNV loci on chromosome 1 taken from Conrad et al. (B). Values are sorted by increasing concordance. While the vast majority of loci showed high concordance, a small subset of loci displayed low concordance (in the Conrad et al. comparison the latter were slightly enriched for small or SD-enriched CNV loci), implying genotyping errors by CopySeq or the respective microarray-based platforms.
(0.25 MB TIF)

Figure S19 Approximately linear dependency between locus copy-number and read-depth ratio. This figure shows the distribution of adjusted read-depth ratios for 99 CNV loci on chromosome 1 among 118 individuals for which we had prior information in terms of copy-number genotype assignments [2], i.e., individual densities were generated by using the previously published genotype information and grouping our read-depth data according to the genotype assignments in [2]. Copy-number densities are indicated by colors (left to right): ‘0’, red; ‘1’, orange; ‘2’, grey; ‘3’, blue; ‘4’, purple. Thus, for example, all loci classified in [2] having CNG = ‘0’ appear in the density plot in the red density class. The number of data points per density class is: ‘0’ = 322, ‘1’ = 888, ‘2’ = 10,184, ‘3’ = 100, ‘4’ = 21. The plot shows that the expected read-depth ratio mean of a copy-number genotype class scales approximately linearly with copy-number. The mean read-depth ratio values for the copy-number genotype classes (i.e., ‘0’ = 0.11, ‘1’ = 0.54, ‘2’ = 0.99, ‘3’ = 1.44, ‘4’ = 1.96) show good correspondence with the theoretical expected values (‘0’ = 0, ‘1’ = 0.5, ‘2’ = 1, ‘3’ = 1.5, and ‘4’ = 2). Of note, this analysis is obviously heavily depending on the genotype assignments made in [2].
(0.25 MB TIF)

Table S1 Individuals analyzed in this study and numbers of filtered sequencing reads considered. All analyzed individuals were recently sequenced in the “pilot 1 project” of the 1000GP at relatively low sequencing coverage. Samples for which high-quality sequencing data were available included one parent-offspring trio of European ancestry, which consisted of father (NA12891), mother (NA12892), and daughter (NA12878). 20 samples were excluded based on the total variance of G+C-adjusted read-depth ratios that we measured in the normalization loci (see Figure S4B). Most excluded samples were sequenced with very low sequence-depth (i.e., <1-fold haploid coverage; see Figure S4B). We applied a variance cutoff of 0.01 for excluding samples.
(0.04 MB XLS)

Table S2 Genotype concordance with SNP array-based calls on the chromosome 1 test set.
(0.03 MB DOC)

Table S3 List of OR loci considered in the analysis. The table lists all 808 OR loci (Build 36.1; hg18) and indicates for each OR locus whether the DGV and a recent survey not listed in DGV [10] have reported CNVs in these loci. Also, genotype frequencies are indicated for all copy-number genotypes from “0” to “9”. Furthermore, for bi-allelic loci, allele-frequency calculations are presented for the CNV allele and the reference. For bi-allelic and multi-allelic loci the reference allele frequency together with a 95% confidence interval was calculated. We removed manually OR6R2P from the output, following the identification of an exact copy of that pseudogene in the unassembled fraction of the reference genome (hg18).
(0.20 MB XLS)

Table S4 Table of copy-number genotype calls in 150 samples and 808 OR loci.
(0.81 MB XLS)

Table S5 Table of copy-number genotype calls in 150 samples and 99 chromosome 1 benchmark CNV loci.
(0.11 MB XLS)

Table S6 Outcomes copy-number genotyping on chromosome 1 benchmark set.
(0.04 MB DOC)

Table S7 High-confidence copy-number genotyping on benchmark set.
(0.04 MB DOC)

Table S8 Outcomes CNV identification with CopySeq on chromosome 1 benchmark set.
(0.04 MB DOC)

Table S9 Outcomes copy-number genotyping integrated with paired-end mapping on chromosome 1 benchmark set.
(0.04 MB DOC)

Table S10 Outcomes copy-number genotyping integrated with breakpoint junction library analysis on chromosome 1 benchmark set.
(0.04 MB DOC)

Table S11 Summary of detected CNVs affecting OR loci.
(0.03 MB DOC)

Table S12 Identified SNPs that lead to OR gene inactivation and pseudogenization. The table lists 73 SNPs that were identified in the 1000GP data, which were predicted to be deleterious to the respective OR gene (i.e., they either result in a premature stop codon or mutate the first methionine).
(0.03 MB XLS)

Table S13 Inferred copy-number genotypes based on qPCR for 5 OR loci.
(0.03 MB XLS)

Table S14 Summary of additional qPCR experiments in 4 OR loci with discordant copy-number genotypes.
(0.03 MB XLS)

Table S15 Copy-number genotyping concordance between CopySeq and custom Agilent CGH arrays (Conrad et al.)
(0.04 MB DOC)

Table S16 Copy-number genotyping concordance between CopySeq and custom Agilent CGH arrays (Conrad et al.) in regions that do not intersect with SDs.
(0.04 MB DOC)

Table S17 Copy-number genotyping concordance between CopySeq and two array platforms in large CNVs intersecting with SDs.
(0.04 MB DOC)

Table S18 Comparison of CopySeq copy-number genotyping with the read-counting approach by Alkan and coworkers.
(0.05 MB DOC)

Table S19 qPCR validation of copy-number genotypes in 18 CNV loci on chromosome 1.
(0.16 MB DOC)

Table S20 qPCR primer sequences.
(0.06 MB DOC)

Table S21 Concordance of CopySeq and fluorescent in situ hybridization (FISH) results.
(0.05 MB DOC)
