# Supplementary material for: Systematic Inference of Copy-Number Genotypes from Personal Genome Sequencing Data Reveals Extensive Olfactory Receptor Gene Content Diversity
Source: PLoS Comput Biol. 2010 Nov 11;6(11):e1000988. doi: 10.1371/journal.pcbi.1000988 (PMC2978733; doi:10.1371/journal.pcbi.1000988)
Supplement: Text S1 — Supplementary Information for “Systematic Inference of Copy-Number Genotypes from Personal Genome Sequencing Data Reveals Extensive Olfactory Receptor Gene Content Diversity”. (0.23 MB DOC) [file pcbi.1000988.s001.doc]

**Supplementary Information for “Systematic Inference of Copy-Number Genotypes from Personal Genome Sequencing Data Reveals Extensive Olfactory Receptor Gene Content Diversity”**

### Sebastian M. Waszak, Yehudit Hasin, Thomas Zichner, Tsviya Olender, Ifat Keydar, Miriam Khen, Adrian M. **Stütz**, Andreas Schlattl, Doron Lancet, Jan O. Korbel

**Measuring the genotyping concordance between CopySeq and a high-resolution custom array-CGH platform.** We used CopySeq to generate copy-number genotypes for a set of chromosome 1 CNVs that were recently genotyped with a high-resolution custom array-CGH platform from Agilent [1]. These CNVs form a larger set and include a larger number of relatively small CNVs than the chromosome 1 CNV loci previously analyzed with Affymetrix arrays [2]. Altogether, 149 out of 150 individuals assessed in our study were also genotyped by Agilent array-CGH and thus were used to estimate copy-number genotyping concordances between CopySeq and array-CGH. We first applied our mappability filter criterion by requiring at least 500 uniquely mappable *k*-mers per CNV (see Materials and Methods), and found that more than 95% of the 422 chromosome 1 CNV loci assessed by array-CGH were ascertainable by CopySeq according to this criterion (*i.e.,* we excluded only 21 CNV regions based on our mappability cutoff). We analyzed these 401 assessable CNV regions, with sizes ranging from 509 bp to 1.0 Mb (median size 3.1 kb), and obtained an overall genotyping concordance of 89.1% between CopySeq and Agilent arrays [1] (Table S15), *i.e.,* a lower concordance than determined between CopySeq and Affymetrix SNP 6.0 arrays [2] (98.9%, see main text).

To examine the basis of this lower concordance, we next assessed the effect of SV size on the concordance estimates. In particular, we examined whether small CNVs in the set display a lower concordance than large CNVs. Surprisingly, this analysis revealed that the genotyping concordance between CopySeq and Agilent array-CGH was higher for small CNVs (<5 kb) than for larger CNVs (>10 kb) (Table S15). More specifically, we observed the highest genotyping concordance when applying no CNV size cutoff, whereas the lowest concordance was observed for the largest CNV size class. This result was in contrast to our findings with regard to the concordance of CopySeq- and Affymetrix array-based calls, *i.e.,* CopySeq and Affymetrix arrays displayed the highest concordance for large CNVs (see main text). To assess reasons for the relatively low concordance observed for large CNVs we specifically analyzed CNV loci >10 kb that displayed a genotyping concordance between CopySeq and array-CGH of <10%. We found that 21 out of the 401 CNV loci fulfilled these criteria, and we aimed to identify the most plausible absolute locus copy-number in these by comparing copy-number genotypes between CopySeq, Agilent array-CGH [1], and Affymetrix arrays [2]. Of note, 20 of the 21 filtered loci (95%) overlapped with annotated SDs (the average portion of the CNV sequence overlapping with SDs in these was 87%). Four of these 21 CNVs were ascertained both with Affymetrix arrays and by Agilent array-CGH (see Table S17), enabling us to compare these loci in detail. All of these four CNVs intersected with SDs. We found that in all four loci the copy-number genotypes were mostly concordant between Affymetrix arrays and CopySeq (Table S17), whereas they were discordant between Agilent array-CGH and CopySeq as well as between Agilent array-CGH and Affymetrix arrays. Despite the discordance in terms of accurate copy-number estimates, however, CopySeq and Agilent array-CGH agreed well in terms of relative copy-number calls (mean Person correlation coefficient = 0.78). As an example, a >115 kb CNV (chr1:1,557,744-1,673,566) in Conrad *et al.* was assigned a copy-number of ‘3’ in most individuals (Table S17) based on array-CGH, while CopySeq assigned it a copy-number genotype of ‘2’ in most of these individuals consistent with Affymetrix SNP 6.0 array assignments. In another case, a >420 kb CNV (chr1:147,300,034-147,726,239) was classified as a multi-allelic duplication based on CGH arrays with copy-numbers ‘3’ and ‘4’ as the most frequent copy-numbers. However, both CopySeq and Affymetrix array-based copy-number genotypes disagreed with array-CGH based genotypes, instead reporting copy-numbers of ‘1’ and ‘2’ in the majority of individuals.

We further assessed a chromosome 1 locus of particular interest, *i.e.,* a CNV region of biomedical relevance containing the *FCGR3B* gene in an SD-rich region [3] (chr1:159,746,569-159,913,382). Although no corresponding CNVs were assessed by Agilent and Affymetrix arrays at this locus, it raised our attention due to its relevance for disease association studies (see below) and due to the strong discrepancy in copy-number genotypes reported for this locus between Agilent array-CGH results and CopySeq (0% concordance). The Agilent array-based study assigned most individuals at this locus a copy-number of ‘4’, however, CopySeq estimated that most individuals own ‘2’ locus copies. Copy-number variation within this locus was previously associated with autoimmune and inflammatory diseases [3]. Several studies reported [3,4,5] with different assays probing several positions in the locus – qPCR, semi-quantitative PCR, array-CGH, and multiplex ligation-dependent probe amplification – that most individuals carry ‘2’ copies at this locus, consistent with CopySeq’s genotyping results. This CNV locus illustrates a case where accurate copy-number genotyping may be relevant for the outcome of disease association studies. Furthermore, the above examples suggest that CopySeq can generate accurate absolute copy-number calls in SD-rich CNV regions by evaluating the uniquely mappable portion of these regions.

Given the discrepancies of CopySeq and Agilent CGH arrays in CNV regions embedded in SDs, we next focused on ‘unique’ regions in which copy-number genotypes can most plausibly be compared in an absolute sense between platforms. We excluded CNV regions assessed with Agilent arrays that intersected with SDs (minimum overlap 1 bp) and in doing so reduced the number of examined CNV regions by approximately 15% (Table S16). Analyzing the reduced set we determined an excellent overall genotyping concordance of 94.8% between CopySeq and Agilent CGH arrays [1]. Furthermore, we observed an increase of genotyping concordance with CNV size (compare with Figure 2A) – after excluding SD regions. For example, CNVs >5 kb and >40 kb displayed a genotyping concordance of 97.3% and 99.8%, respectively (Table S16).

**Comparison of CopySeq to the Alkan *et al.* approach.** We compared CopySeq to the approach published by Alkan *et al.* [6], which interprets averaged read-counts as locus copy-numbers (depth-of-coverage analysis without probabilistic genotyping model). The Alkan *et al.* approach evaluates all genomic mapping positions (including non-unique ones) with the mrFAST [6] algorithm, which – compared to mappers designed for aligning reads onto uniquely mappable genomic regions – runs relatively slowly. On the other hand, mrFAST, in comparison to other read-mappers, has the advantage of being able to ascertain hits also in highly similar segmentally duplicated regions. According to timing estimates published in Alkan *et al.* [6] mrFAST processing of all of the 1000 genomes pilot 1 data would require several weeks on a fairly large-scale computer cluster (*i.e.,* a cluster with hundred processors). We thus reasonably decided to compare CopySeq and the Alkan *et al.* approach by focusing on a published dataset that was previously analyzed with mrFAST [6], *i.e.,* genome sequencing reads of the African individual NA18507, which were generated by Illumina sequencing (these reads were originally published in [7]). Alkan *et al.* mapped these reads onto the reference genome with mrFAST [6] and generated locus copy-number calls for all (*i.e.,* nearly 1000) CNV regions that can be genotyped with Affymetrix SNP 6.0 microarrays [2]. Alkan *et al.* compared their results to Affymetrix array based copy-number genotypes generated by McCarroll *et al.* [2]. To ensure comparability of the data, Alkan *et al.* focused in their comparison on CNV regions that did not intersect with segmental duplications (SDs), *i.e.,* in excluding regions that display an overlap with SDs of at least 1 bp. In this regard, Alkan *et al.* reasoned that in SD-regions microarray-based assignments might be concordant with read-counting in a relative but not in an absolute sense – thus, Alkan *et al.* decided to exclude those regions when comparing their results with Affymetrix array-based results.

In order to reasonably compare CopySeq and Alkan *et al.*’s approach [6] we obtained published NA18507 genome sequencing data [7] from the short read archive (http://www.ncbi.nih.gov/sra). We mapped the reads with BWA [8] (mapping quality cutoff = 10) and used CopySeq to generate genome-wide copy-number genotypes for all McCarroll *et al.* CNV regions. We excluded all CNV regions with SD-overlap ≥1 bp (apart from removing these SD-overlapping regions we did not remove any further CNV regions, *e.g.,* on the basis of repeat-content or read-mappability). As can be seen in Table S18, CopySeq achieved an overall higher concordance with Affymetrix arrays (97.2%) than the Alkan *et al.* approach (80.2%). Although the improved concordance was particularly evident for short CNV regions (*i.e.,* regions ≥1 kb), also in larger regions (*e.g.,* ≥10 kb) CopySeq achieved higher concordances than the Alkan *et al.* approach (Table S18).

**qPCR validation of predicted copy-number genotypes on chromosome 1.** We selected 18 common CNVs randomly from chromosome 1, with sizes ranging from 707 bp to 127 kb and a median size of 3.7 kb, resulting in a set comprising 13 CNV regions previously analyzed with Agilent array-CGH [1] and 5 previously analyzed by Affymetrix SNP 6.0 arrays [2] (note that two CNV regions were analyzed both by array-CGH and Affymetrix SNP 6.0 arrays previously). Quantitative real-time PCR (qPCR) was performed on the European parent offspring trio (NA12878, NA12891, and NA12892) and Ct values were normalized to facilitate the comparability between CopySeq’s copy-number genotypes and qPCR results. In 16 out of the 18 loci locus copy-numbers were inferred from the qPCR results, *i.e.,* (*i*) from the absence of a signal indicating a copy-number of ‘0’, and (*ii*) from differences in Ct values of exactly ‘1’ ΔCt, which enabled us to discriminate locus copy-numbers of ‘1’ and ‘2’.

In two loci, no signal differences were observed among the three samples, which agreed – in a *relative* sense – with the prediction of CopySeq that there are no locus copy-number differences among the assessed individuals (NA12878, NA12891, and NA12892) in these loci. As can be seen in Table S19, 49 out of 54 (91%) copy-number genotype assignments in three samples were supported by the qPCR experiments, *i.e.,* 12 out of 12 homozygous deletions (copy-number genotype=‘0’), 18 out of 23 heterozygous deletions (copy-number genotype=‘1’), and 19 out of 19 homozygous reference alleles (copy-number genotype=‘2’). The discrepant genotypes resulted from three relatively small CNVs (*i.e.,* 958 bp, 1.6 kb, and 2.3 kb) and the GC-adjusted read-depth ratios were borderline in all three cases, with values between 0.68-0.76 (note, a read depth ratio of 0.5 corresponds to a copy-number genotype of ‘1’ and a read depth ratio of 1 corresponds to a copy-number genotype of ‘2’).

**CNV allelic status assignment.** CNV loci were inferred as *multi-allelic*, rather than *bi-allelic*, if deletions and duplications were observed at the same locus or if copy-number genotypes >’4’ were observed. Out of 42 identified multi-allelic loci, 20 were defined as such based on a copy-number genotype assignment of a single sample contradicting a bi-allelic pattern (Table S4). In 10 of these 20 cases, a single duplication was recorded in a locus otherwise dominated by deletions; in 6 cases a single deletion was recorded in a locus otherwise dominated by duplications, and in the 4 remaining cases only two genotypes, *i.e.,* a deletion and a duplication, respectively, were inferred. Classifications of CNV loci as multi-allelic supported by ≥2 copy-number genotypes were termed *high support* classifications.

**Allele frequency estimation in multi-allelic OR loci.** While for bi-allelic loci the exact allelic combination (homozygosity or heterzygosity of the reference and CNV) can be inferred unambiguously from copy-number genotypes, this is not the case in multi-allelic loci where different combinations of alleles may yield the same copy-number genotype. Thus, to approximate the reference allele frequency in multi-allelic loci we assumed that all copy-number genotypes of ‘2’ represent a homozygous combination of the reference allele. Note that this assumption is valid for loci displaying multiple duplication alleles and no deletion alleles. Furthermore, the assumption may be reasonable also for multi-allelic loci displaying both deletions and duplications, *i.e.,* when either the deletion or the duplication alleles (or both) are rare, which makes it unlikely that a copy-number genotype of ‘2’ results from a combination of a duplication and a deletion. Given that these conditions were met for all multi-allelic OR loci, we approximated reference allele frequency in multi allelic loci as square root of the frequency at which the copy-number genotype ‘2’ was observed. For example at OR11H13P locus there are multiple duplication alleles (copy-number genotype range is ‘2’ to ‘8’), and copy-number genotype ‘2’ was inferred in 44 individuals, thus we estimated the reference allele frequency *f_r_* as *f_r_* = = 54.2%. According to this analysis, in one multi-allelic locus, OR11J2P, the reference sequence represented the minor allele with an allele frequency of 21.6% in all populations while in an additional three cases (OR7E86P, OR11H13P, OR11K1P) the reference represented the minor allele in at least one population.

**Reference allele frequency confidence interval calculation**. We examined the abundance of OR loci displaying a minor reference allele frequency based on observed allele frequencies in 150 individuals. We also calculated confidence intervals, as follows, to reflect the uncertainty in identifying minor alleles from measurements in 150 individuals. Namely, we calculated the 95% confidence interval for reference allele frequencies with $\hat{f}_{r}\pm1.96\sqrt{\hat{f}_{r}(1-\hat{f}_{r})/n}$, where $\hat{f}_{r}$ is the reference allele frequency estimate and *n* is the sample size (*i.e.*, 150 in this case). Based on this calculation we classified OR loci into a transition group of both major and minor allele, if the 95% confidence interval spanned the 50% allele frequency value. For example, we classified the OR pseudogene OR4A45P into the transition group of minor/major allele, because the reference allele frequency falls within the interval (46.7%, 62.7%) with 95% probability.

**Identification of CNVs bi-allelic in one population and multi-allelic in another.** Among 22 multi-allelic OR loci inferred with ‘high support’, we observed 11 OR loci that appeared multi-allelic in at least one population and bi-allelic in at least one other population. These 11 map to four genomic OR clusters, with a single cluster (14@19.5) on chromosome 14 encompassing eight ORs. In the analyzed YRI individuals this cluster displayed copy-number genotypes of ‘2’, ‘3’, and ‘4’, consistent with a bi-allelic duplication, whereas in Europeans and Asians copy-number genotypes of ‘5’ and ‘6’ were observed in several individuals (see Table S4). Such a copy-number genotype distribution could be explained by a lower frequency of one of the CNV alleles, ‘loss’ of one allele in the Africans, or a ‘gain’ of the allele in a common ancestor of the European and Asian populations.

**Identifying reference genome idiosyncrasies.** We observed a number of autosomal loci in which CopySeq never, or extremely rarely, displayed a copy-number genotype of ‘2’ (*i.e.*, cases in which a CNV was identified in all, or nearly all, analyzed individuals). One possible explanation for such an observation is that the reference genome (hg18) displays a rare CNV at such loci (alternatively, such observations could correspond to assembly errors in the reference). One example involves the two genes OR4C3 and OR4C5 and the pseudogene OR4C4P. Mining data from a recently published personal genome assembly [9] (HuRef) we noticed the presence of a genomic cluster of three pseudogenes (OR4C49P, OR4C45P, and OR4C48P) that are not in the hg18 assembly but share high sequence identity with OR4C3, OR4C5, and OR4C4P. Sequencing reads from all 150 individuals can be uniquely mapped to that cluster, indicating that despite their absence from the reference genome these three pseudogenes are present in the vast majority of YRI, CEU, CHB, and JPT individuals.

A similar case is evident at the OR9G1 locus. According to the HuRef assembly [9] the reference genome represents a deletion allele at this site. The deletion includes OR9G9, which is a close paralog of the OR9G1 that does occur in hg18. As a result, reads originating from OR9G9 may increase the measured depth-of-coverage of the OR9G1 locus. Therefore, the observed copy-number genotypes of ‘2’ and ‘3’ for the OR9G1 locus may in fact correspond to a copy-number genotype of ‘2’ for OR9G1 and copy-number genotypes of ‘0’ and ‘1’ for OR9G9. The fact that copy-number genotypes of ‘2’ and ‘3’ were frequently observed in our study suggests that the deletion allele affecting OR9G9 is relatively common (Table S4).

**Contribution of the OR7E subfamily to the differential abundance of CNVs in OR pseudogenes and OR genes.** Whether OR pseudogenes and genes are differentially affected by CNVs has been a matter of some discussion in the past. As stated in the main text, our analysis confirmed the trend that OR pseudogenes are more frequently affected by CNVs than OR genes [10]. We went on analyzing the contribution of particular OR pseudogene subfamilies and found that much of the difference between OR genes and pseudogenes is attributable to the OR7E subfamily, an OR subfamily consisting of 85 pseudogenes and only one gene. The OR7E subfamily, which is embedded within complex segmental duplications, has been reported as rapidly expanding [11] and as involved in disease-causing chromosomal rearrangements [12] in humans. Among OR pseudogenes, members of the OR7E subfamily constitute ~57% (8/14) of the homozygously deleted pseudogenes, while their proportion within the entire OR pseudogene set is only ~18%. In the entire dataset, OR deletions were 2-fold more frequent found in pseudogenes than in genes, and homozygous deletions were 8-fold more likely in pseudogenes than in genes (see main text). Removal of the OR7E family from our analysis results in more similar rates of deletion between pseudogenes and intact ORs, although the overall trend of an increased variability in OR pseudogenes persisted: on average slightly more pseudogenes than genes were deleted in each individual (1.04% *vs.* 0.97% per sample, respectively), and homozygous deletions were 2-fold more often affecting pseudogenes (0.22% per sample) than genes (0.11% per sample).

**Additional qPCR experiments in OR loci with discordant copy-number genotypes.** We further tested by qPCR the four OR loci displaying the least concordance between CopySeq and microarray-based approaches (Table S14). The following eight samples were assessed to enable evaluating discrepant calls: NA11881, NA11920, NA11994, NA11995, NA12003, NA12045, NA12155, and NA12716. Primer placement was carried out manually, and positions harboring SNPs (dbSNP 130) were excluded from the primer design to ensure locus specificity.

In the first locus (OR4A45P), the data observed in the qPCR measurements agreed with CopySeq-based copy-number genotypes, rather than with the microarray-based copy-number genotypes; in particular, the qPCR supported the presence of a homozygous deletion as no signal was observed in those individuals as inferred by CopySeq, rather than a duplication as inferred in [1] (Figure S14A). Furthermore, the presence of the deletion, as predicted by CopySeq, is supported by a ~3.4 kb deletion at the corresponding locus in the Celera human genome assembly; in addition, the deletion is supported by a deletion entry in dbSNP (‘rs67661836’).

In the second locus (OR11K1P), CopySeq’s copy-number genotypes were discrepant from the array-CGH based study [1], but were concordant with the Affymetrix microarray-based study [2] (Figure S14B). The qPCR results for this locus were concordant with CopySeq and Affymetrix arrays, *i.e.,* they agreed with the presence of a multi-allelic duplication, which was not detected by array-CGH in these individuals [1].

In the third locus (OR5H5P) the almost identical normalized Ct values between individuals (*i.e.,* qPCR values in the normal range) suggested that no CNV is present at the locus, as predicted by CopySeq, rather than homozygous and heterozygous deletions, as predicted in [2] (Figure S14C).

In the fourth locus (OR4N4) the qPCR results suggest the presence of a CNV (Figure S14D), thus supporting both the array-based study [2] and CopySeq. In this case, however, the resolution of the qPCR data was insufficient to assess whether the concordance was higher between qPCR and the array-based study or between qPCR and CopySeq.

**Influence of sequencing depth on copy-number genotyping.** In order to assess to what extent CopySeq‘s genotyping accuracy depends on the sequencing coverage, we generated 10 different coverages by down-sampling DNA reads from the published NA18507 genome [7]. The sequence coverages we used were 0.5x, 1x, 2x, 3x, 4x, 5x, 10x, 20x, 30x, and 40x. CopySeq was applied on these read sets using default parameters (with requested LOD score >0). We compared CopySeq calls to two genome-wide copy-number genotype callsets for NA18507, *i.e.,* the sets published by Conrad *et al.*, and McCarroll *et al*. We excluded CNVs overlapping SDs, owing to issues in comparing these regions across platforms (see above). Figure S16 displays the results of these comparisons in terms of estimated lower boundary genotyping errors (calculated as #false genotypes/(#true genotypes + #false genotypes)), where we conservatively assumed that all array-based copy-number calls are *correct* (all calls discrepant with CopySeq were conservatively assumed to represent *genotyping errors* of CopySeq). Unsurprisingly, with higher sequencing depth the genotyping error decreased. Importantly however, there was only a slight difference in genotyping error between an extensively covered human genome (30-40x) and a genome sequenced with a low coverage typical for the 1000GP (3-4x), *i.e.,* the coverage used in our study. Of note, below 2x coverage we observed a sharp increase in the estimated genotyping error – equivalent to a decrease in accuracy – which suggests the existence of a lower bound for the sequencing coverage. These results strongly support the utility of CopySeq for genotyping CNVs in the 1000GP’s low-coverage data. Although 30-40x genomes generate slightly better results than 3-4x genomes (with a decrease in genotyping error of approximately 0.8-1.6%), the ten-fold cost reduction of low-coverage sequencing relative to high-coverage sequencing makes low-coverage sequencing an attractive approach that enables analyzing large sample sets at low cost.

**Simulation of sequencing errors and read-mapping onto paralogous loci.** CopySeq’s genotyping approach evaluates reads mapping *uniquely* onto the genome, *i.e.,* such aligned onto “mappable” genomic regions. This rationale implies that biases may occur in the event of “read mis-mapping” to incorrect, non-specific genomic regions owing to misalignment. To assess whether such biases may have affected our analyses we went on assessing whether in the regions of interest of this study – *i.e.,* all our OR regions, paralogous loci which to some extent are equivalent to segmental duplications – may have facilitated the mis-mapping of reads in the presence of SNPs or sequencing errors by altering the read-depth at non-specific paralogous sites. Thus, we examined the influence of sequencing errors and SNPs on OR regions with extensive simulations. We generated two identical read-sets by randomly sampling paired 36-mer reads from the genome: the first set (“set A”) by drawing reads from OR regions without sequencing error; the second set (“set B”) by introducing sequencing errors into the first set and assuming an Illumina sequencing error probability of 1.5% per nucleotide (see [13]). Of note, the resulting error rate is considerably higher than the rate at which SNPs occur in the genome (~0.1% per nucleotide); thus we reasoned that our simulation also accounted for the effect of SNPs.

We used MAQ [14] to map both read-sets onto build36 of the reference genome. In “set A” (no sequencing error), we observed that 81.5% of the reads aligned uniquely against the genome — unsurprisingly, *all* (100%) of these uniquely aligned reads mapped onto the OR region they originated from (0% mis-mapping rate). For “set B” (with sequencing error), 81.0% of all reads aligned uniquely against the genome, and 99.9% of the uniquely aligned reads mapped onto the OR region they originated from (*i.e.,* 0.1% mis-mapping rate). That means, on average, ~0.5% (*i.e.,* the percentage of uniquely aligned reads in “set A” minus “set B”) of all reads were not aligned uniquely due to the introduced sequencing errors, *i.e.,* they were filtered out (discarded) owing to resultant multiple genomic hits displaying a single nucleotide mismatch. In addition, on average ~0.1% of all reads in “set B” mapped “off target” (*i.e.,* onto a paralogous region, typically another OR region).

Overall, the maximum “local read-loss” (*i.e.,* the missing of reads owing to their alignment to a “false” region or owing to non-unique mapping) we observed for an OR region was 7% (average <1%). Furthermore, the maximum “local read-gain” (*i.e.,* the introduction of reads originating from elsewhere in the genome into the OR region of interest) was 3% (average <0.1%). Of note, we observed the maximum mis-mapping rates in very recently duplicated OR regions from the OR7E family – the vast majority of other OR genes and pseudogenes displayed very little mis-mapping.

We went on to assess to what extent the mis-mapped reads (despite being relatively rare) may have affected CopySeq’s genotyping accuracy. Using our simulation data we estimated rates for “local read-gain” and “local read-loss” independently for each OR region, and then assessed the consequence of both forms of mis-mapping on copy-number genotyping. Namely, we sub-sampled reads from the previously published NA18507 genome to 3x coverage and systematically altered OR region specific read-counts according to the locus-specific “local read-gain” and “local read-loss” residuals. In our simulations only <0.5% of the OR copy-number genotype calls were affected by this (*i.e.,* changed their absolute value), demonstrating that sequencing errors (and hence also SNPs) have overall a very small effect. With ongoing improvements in the sequencing chemistry of state of the art NGS platforms (*i.e.,* enabling longer read lengths) this effect will further diminish in the future.

**Copy-number genotype predictions in variable loci validated by Alkan *et al.* with FISH**. We further tested CopySeq’s performance by genotyping CNV regions that were validated by Alkan *et al.* using FISH with ~40 kb fosmid probes in the high-coverage Bentley *et al.* (NA18507) genome. As noted before (*e.g.,* [15,16]) FISH probes containing SD blocks may lead to multiple signals in the genome, reflecting hybridizing paralogous sequences. In order to facilitate the comparison of FISH results with our CopySeq approach –a method focusing on unique, mappable regions – we first focused on regions assessed by FISH in [6] that do not fall into SDs. Only one region matches this criterion (Table S21); in this region the inferred locus copy-number agreed between FISH and CopySeq. To further test the abovementioned notion that FISH in effect measures the “sum of paralogues sequences” in regions where FISH probes hybridize to several paralogs [15], we further extended this analysis by assessing all four FISH regions from [6] that contain exactly one annotated SD. (Of note, we excluded FISH probes interspersed with multiple underlying SDs, as such complex structures may lead to ambiguous results when relating FISH results to a specific genomic locus.) Note that according to Sharp *et al.*’s argument FISH probes encompassing exactly one SD should measure a copy-number of ‘4’ in the absence of copy-number variation, since the FISH probe would hybridize to both alleles of each SD paralog. On the other hand, CopySeq should measure paralog-specific copy-numbers of ‘2’ for each paralog under the same scenario, the genome-wide sum of which would exactly match the FISH measurements (*i.e.*, ‘2’+’2’=‘4’). We assessed this idea by CopySeq genotyping the genomic region covered by the FISH probe in each case for (*i*) the locus the FISH probe was designed for, and (*ii*) the corresponding paralogous locus defined through the SD. In three out of the four FISH regions the sum of paralogous CopySeq copy-number predictions was indeed identical to the number of signals detected by FISH (see Table S21). For example, one FISH probe (chr16:68,708,494-68,749,105) detected ‘5’ copies genome-wide, whereas CopySeq detected ‘3’ copies for one locus and ‘2’ copies for its paralog (*i.e.,* equivalent to ‘5’ genome-wide). In the one discordant copy-number measurement we identified the inferred copy-numbers differed by a single quantitative copy (*i.e.,* FISH = ‘3’ and CopySeq’s “sum-of-paralogous-sequences” = ‘2’) – implying a wrongly called copy-number genotype for one of the paralogous loci. Taken together, in four out of five assessed regions (80%), CopySeq results were consistent with the FISH results, providing further supporting evidence for the accuracy of CopySeq’s genotype calls. Of further note, CopySeq “segmented” the paralog-unspecific FISH results into locus-specific copy-number genotypes – the genome-wide sum of which, as expected, exactly corresponded to the FISH results. These results demonstrate the ability of CopySeq to provide locus-specific copy-number values for the underlying paralogs, and thus to add value to FISH results by pointing at the respective locus that varies in copy-number.

**References**

1. Conrad DF, Pinto D, Redon R, Feuk L, Gokcumen O, et al. (2010) Origins and functional impact of copy number variation in the human genome. Nature 464: 704-712.

2. McCarroll SA, Kuruvilla FG, Korn JM, Cawley S, Nemesh J, et al. (2008) Integrated detection and population-genetic analysis of SNPs and copy number variation. Nat Genet 40: 1166-1174.

3. Fanciulli M, Norsworthy PJ, Petretto E, Dong R, Harper L, et al. (2007) FCGR3B copy number variation is associated with susceptibility to systemic, but not organ-specific, autoimmunity. Nat Genet 39: 721-723.

4. Asano K, Matsushita T, Umeno J, Hosono N, Takahashi A, et al. (2009) A genome-wide association study identifies three new susceptibility loci for ulcerative colitis in the Japanese population. Nat Genet 41: 1325-1329.

5. Breunis WB, van Mirre E, Geissler J, Laddach N, Wolbink G, et al. (2009) Copy number variation at the FCGR locus includes FCGR3A, FCGR2C and FCGR3B but not FCGR2A and FCGR2B. Hum Mutat 30: E640-650.

6. Alkan C, Kidd JM, Marques-Bonet T, Aksay G, Antonacci F, et al. (2009) Personalized copy number and segmental duplication maps using next-generation sequencing. Nat Genet 41: 1061-1067.

7. Bentley DR, Balasubramanian S, Swerdlow HP, Smith GP, Milton J, et al. (2008) Accurate whole human genome sequencing using reversible terminator chemistry. Nature 456: 53-59.

8. Li H, Durbin R (2009) Fast and accurate short read alignment with Burrows-Wheeler transform. Bioinformatics 25: 1754-1760.

9. Levy S, Sutton G, Ng PC, Feuk L, Halpern AL, et al. (2007) The diploid genome sequence of an individual human. PLoS Biol 5: e254.

10. Hasin Y, Olender T, Khen M, Gonzaga-Jauregui C, Kim PM, et al. (2008) High-resolution copy-number variation map reflects human olfactory receptor diversity and evolution. PLoS Genet 4: e1000249.

11. Newman T, Trask BJ (2003) Complex evolution of 7E olfactory receptor genes in segmental duplications. Genome Res 13: 781-793.

12. Giglio S, Calvari V, Gregato G, Gimelli G, Camanini S, et al. (2002) Heterozygous submicroscopic inversions involving olfactory receptor-gene clusters mediate the recurrent t(4;8)(p16;p23) translocation. Am J Hum Genet 71: 276-285.

13. Korbel JO, Abyzov A, Mu XJ, Carriero N, Cayting P, et al. (2009) PEMer: a computational framework with simulation-based error models for inferring genomic structural variants from massive paired-end sequencing data. Genome Biol 10: R23.

14. Li H, Ruan J, Durbin R (2008) Mapping short DNA sequencing reads and calling variants using mapping quality scores. Genome Res 18: 1851-1858.

15. Sharp AJ, Locke DP, McGrath SD, Cheng Z, Bailey JA, et al. (2005) Segmental duplications and copy-number variation in the human genome. Am J Hum Genet 77: 78-88.

16. Linardopoulou EV, Williams EM, Fan Y, Friedman C, Young JM, et al. (2005) Human subtelomeres are hot spots of interchromosomal recombination and segmental duplication. Nature 437: 94-100.
