# Supplementary figures and images for: Systematic Inference of Copy-Number Genotypes from Personal Genome Sequencing Data Reveals Extensive Olfactory Receptor Gene Content Diversity
Source: PLoS Comput Biol. 2010 Nov 11;6(11):e1000988. doi: 10.1371/journal.pcbi.1000988 (PMC2978733; doi:10.1371/journal.pcbi.1000988)

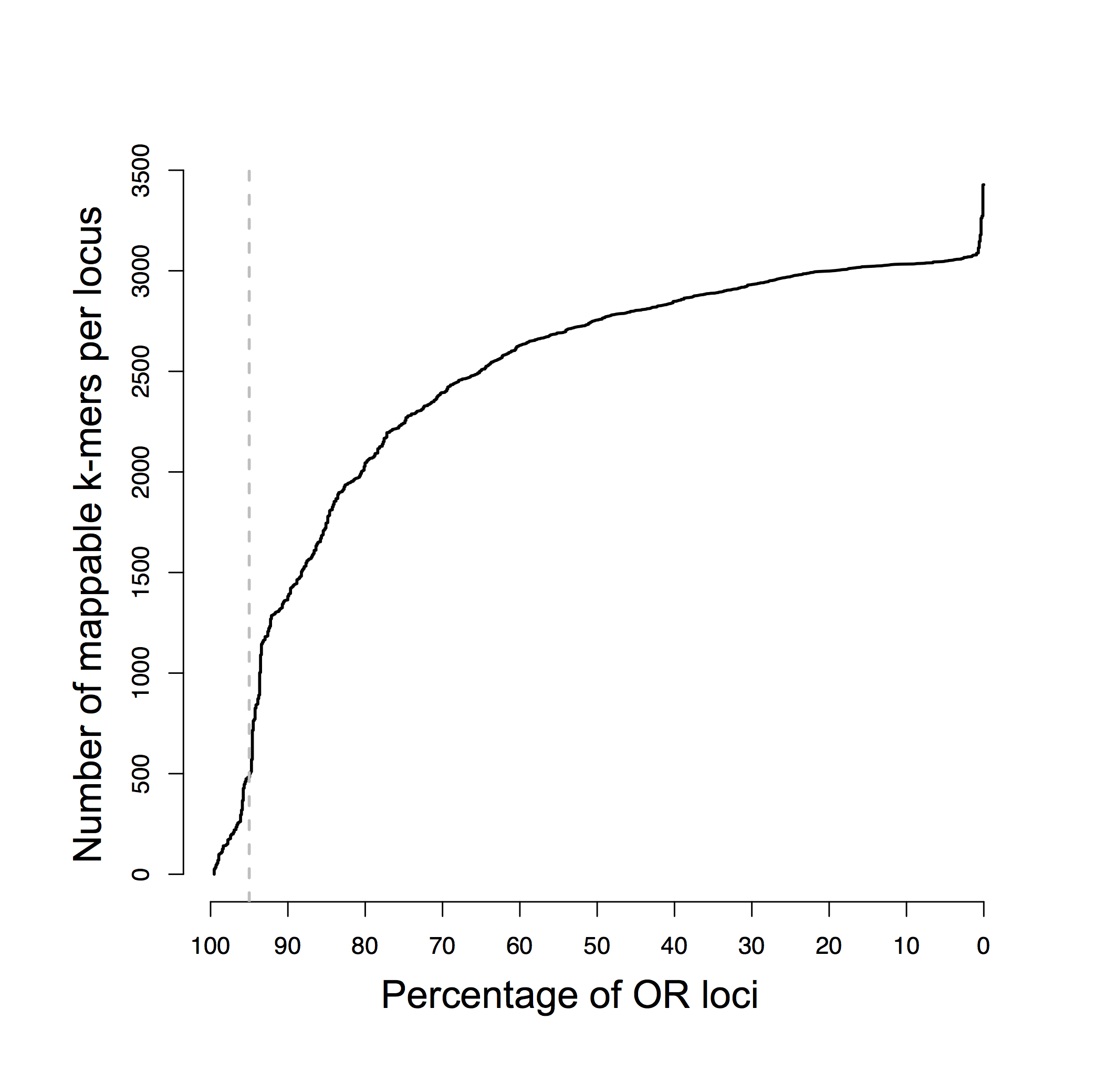

Supplement: Figure S1 — Mappability of DNA sequences in OR loci. OR loci, regions with a median length of 3kb (including upstream and downstream regions), are sorted based on the number of uniquely mappable k-mers (i.e., one exact occurrence in the reference genome, build 36.1) per OR locus, with k = 36nt. Values on the y-axis, referring to the number of unique k-mers per locus, are expressed as starts (i.e., start sites) of 36-mer reads mapped onto the locus. Values on the x-axis are expressed in terms of the fraction of OR loci (in percent). For example, ∼95% of the total set of 851 OR loci in the reference genome have at least ∼500 mappable 36-mers (indicated by dashed line), and were thus considered in our study. (0.20 MB TIF) [file pcbi.1000988.s002.tif]

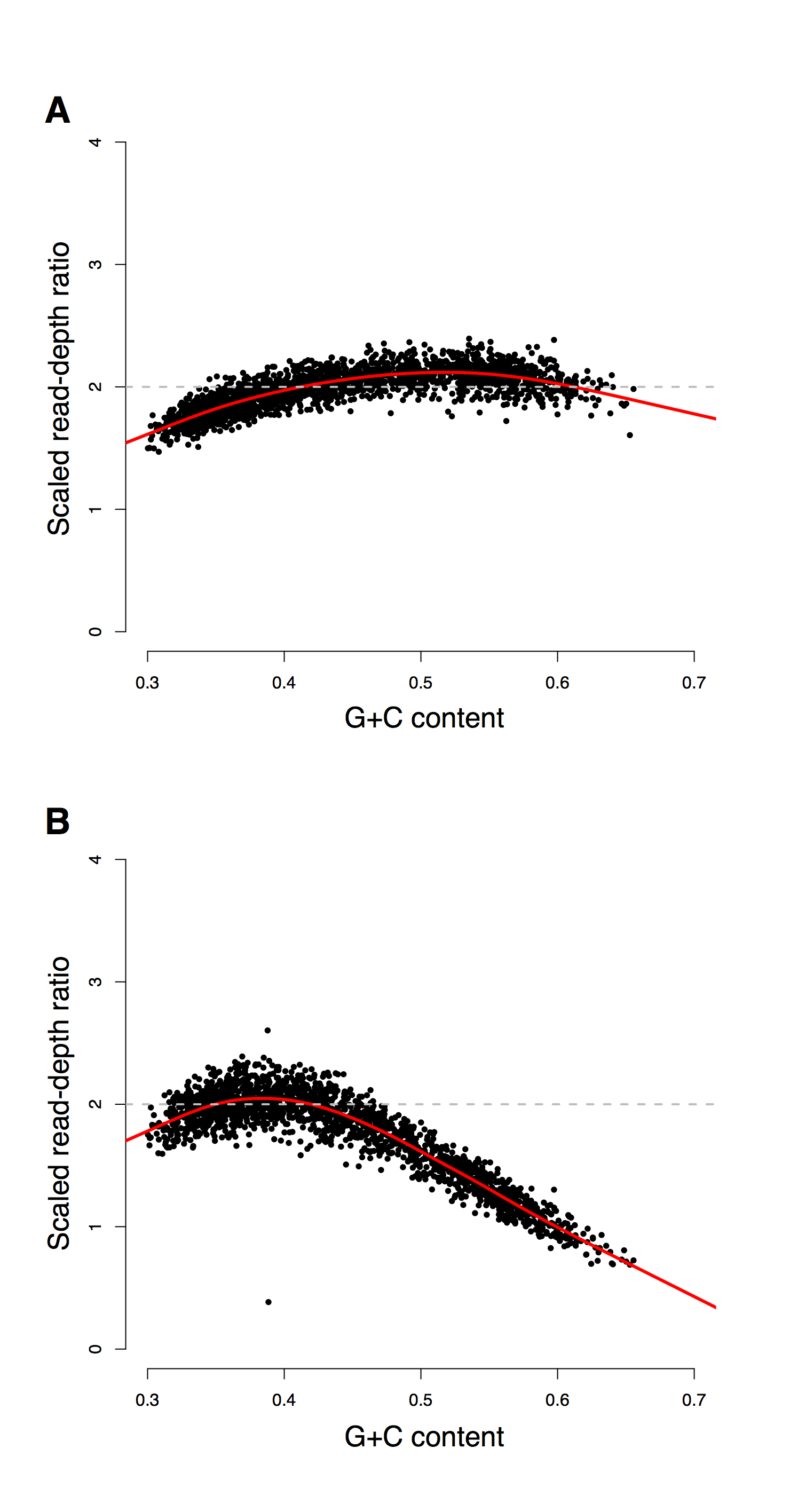

Supplement: Figure S2 — Sample-specific G+C-content bias normalization. Dots indicate the G+C content (or ‘GC content’, calculated as G+C/(A+C+T+G)) and raw read-depth ratios (i.e., #expected/#observed reads) measured in a set of normalization loci (i.e., copy-number invariable loci; see Materials and Methods). G+C bias was corrected by analyzing 3,000 10kb loci (i.e., ∼1% of the human genome) in each sample. Although read-depth ratios are expected to be evenly distributed in the genome, the panels A and B show a sample-specific bias in the read-depth of these normalization loci that appears associated with the G+C content; examples displayed correspond to NA10851 (A) and NA19238 (B). For each sample, G+C characteristics were fitted using a smoothing spline-based model that accounts for the non-uniform G+C behavior (red line). The G+C model fit served as a normalization curve for all loci assessed in a given sample. (0.25 MB TIF) [file pcbi.1000988.s003.tif]

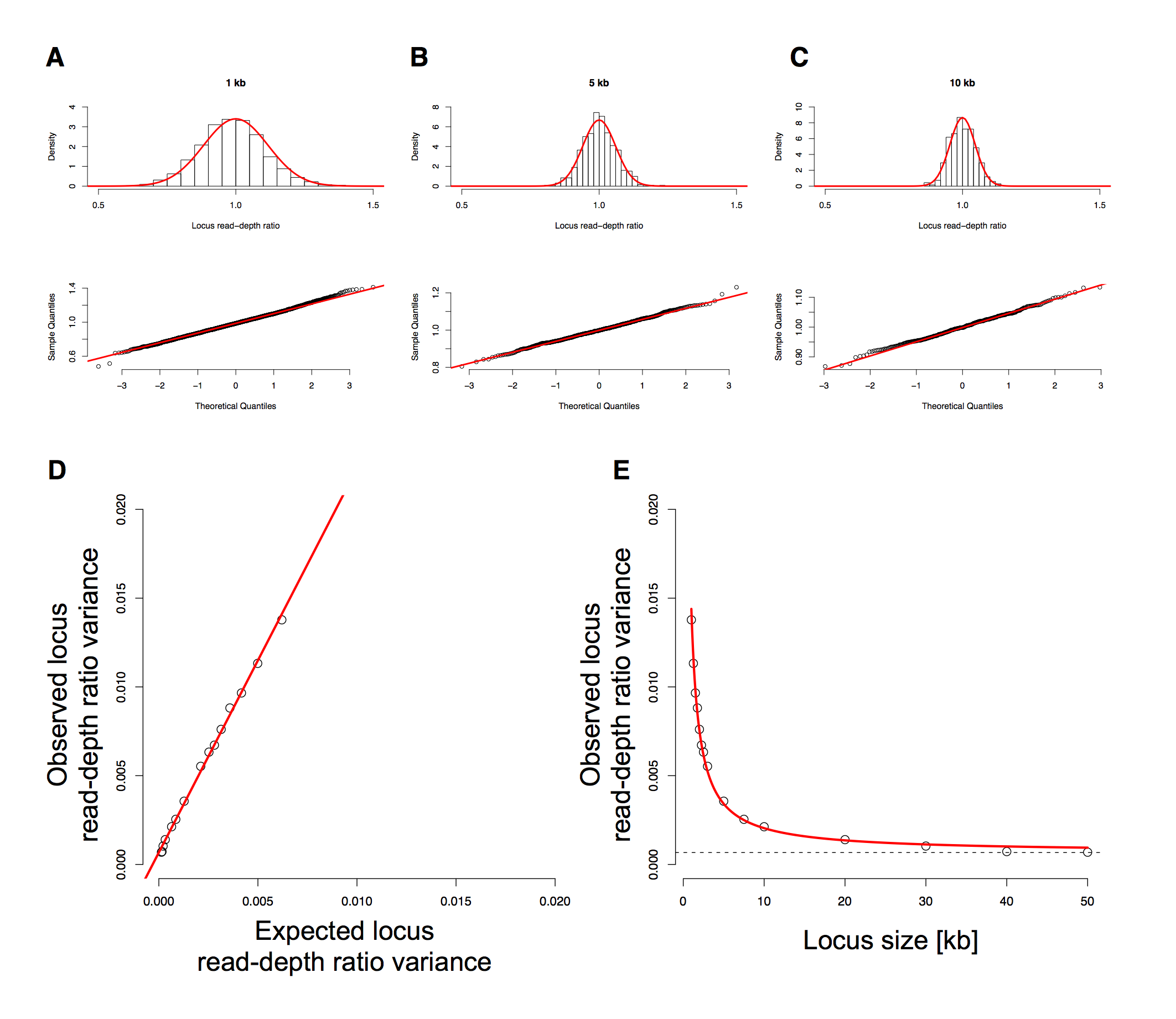

Supplement: Figure S3 — Normality assumption for G+C content-adjusted read-depth ratios and read-depth ratio variance model fit. A–C. The figures display histograms (top) and normal Q-Q plots (bottom) of G+C content-adjusted read-depth ratios of three locus size classes (1 kb, 5 kb, and 10 kb; left to right) in NA10851. The red line, overlaid onto the histograms, displays a normal distribution fit to the histograms. The red line in the Q-Q plots implies that the adjusted read-depth ratio data are approximately normally distributed. D. The figure displays the dependency between the expected Poisson sampling read-depth ratio variance (x-axis) and the observed G+C-adjusted read-depth ratio variance (y-axis) for loci of different size classes (i.e., 1 kb, 1.25 kb, 1.5 kb, 1.75 kb, 2 kb, 2.25 kb, 2.5 kb, 3 kb, 5 kb, 7.5 kb, 10 kb, 20 kb, 30 kb, 40 kb, and 50 kb), measured with constant copy-number (‘2’) in NA10851. The red line represents a linear regression fit to the variance data (see Materials and Methods). E. This figure shows the G+C content-adjusted read-depth ratio variance (y-axis) for different classes of locus length (x-axes; same locus sizes as in (D)) in NA10851. The red line is the fit of the CopySeq variance model to the data (see Materials and Methods). The dashed horizontal line is the estimated copy-number and locus-length independent background variance term. (0.31 MB TIF) [file pcbi.1000988.s004.tif]

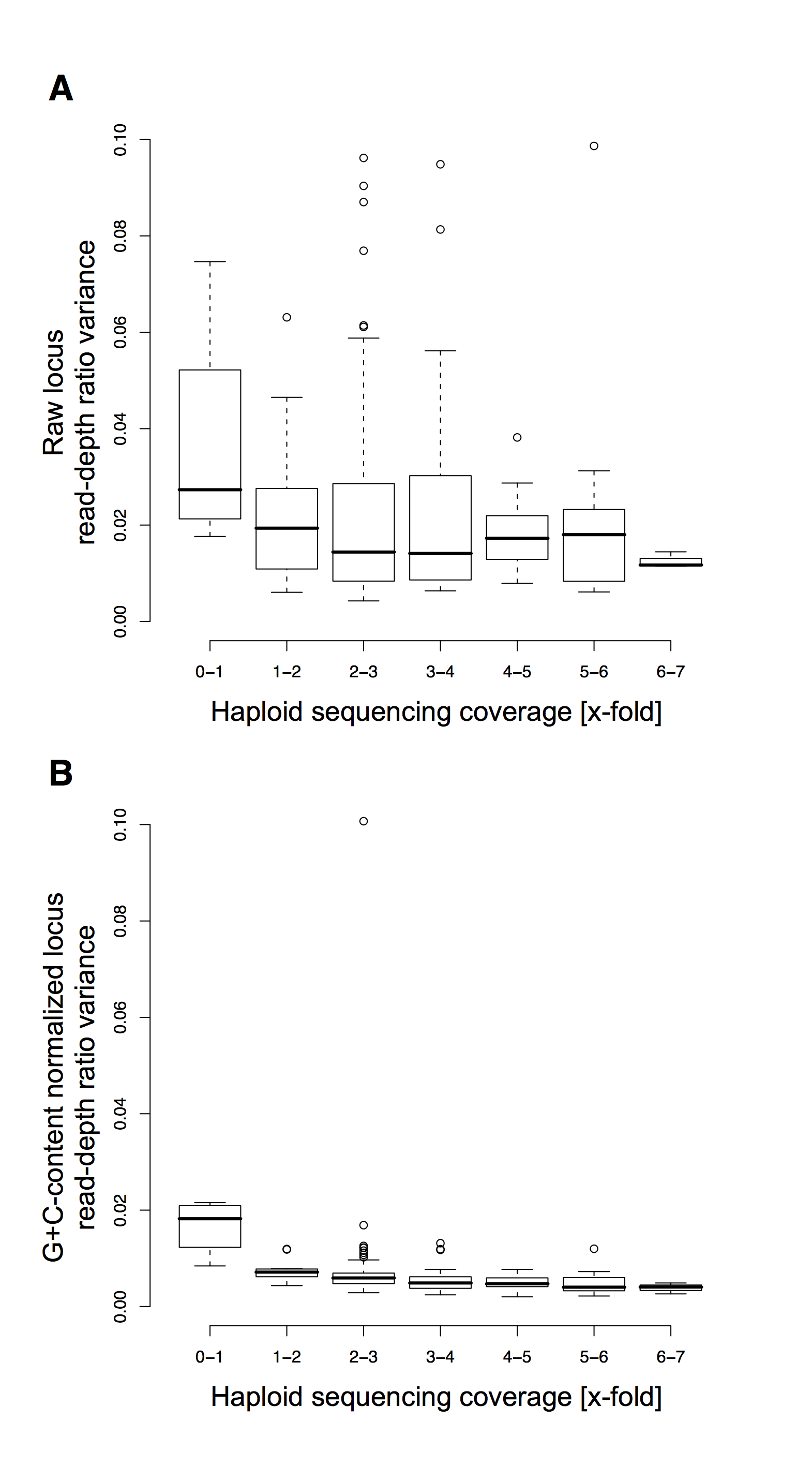

Supplement: Figure S4 — Variance of G+C normalization locus read-depth ratios. The figure relates the variance in read-depth ratio measurements to the genomic sequencing coverage before (A) and after normalization for G+C content (B). Altogether, 170 samples (i.e., including such excluded from further analysis; see Figure S6) were binned according to sequencing coverage. Box plots represent inter-quartile intervals for each bin (25%, 50%, and 75%), and the thick lines the respective median. Altogether, 20 samples were excluded due to increased variance, most of which displayed a very low sequencing coverage (i.e., <1-fold haploid coverage). (0.25 MB TIF) [file pcbi.1000988.s005.tif]

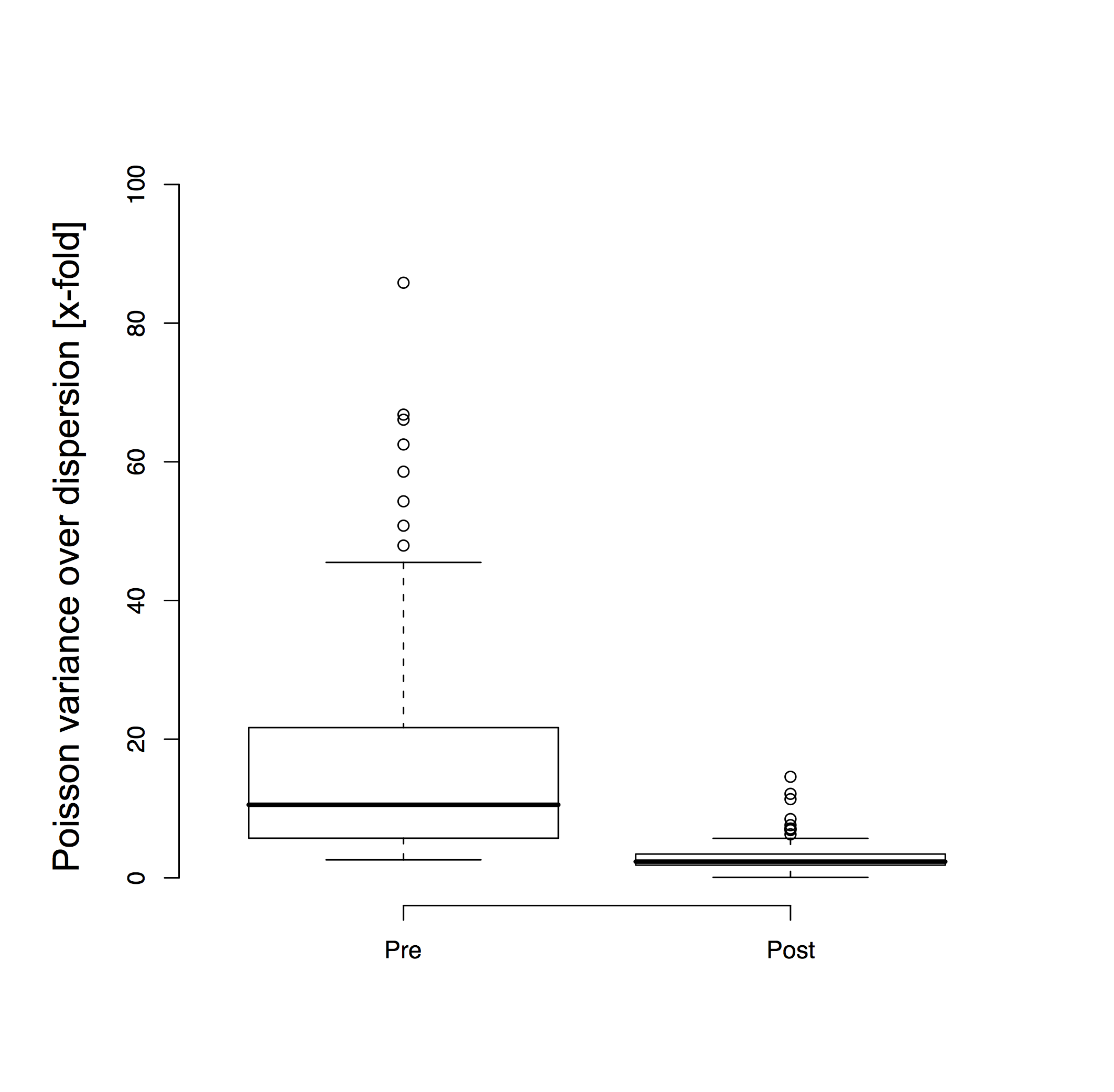

Supplement: Figure S5 — Poisson variance over-dispersion before and after G+C-content normalization. Boxplot of Poisson variance over-dispersion as assessed by control regions in 170 samples before (‘Pre’) and after (‘Post’) G+C content bias correction. Over-dispersion is defined as the n-fold variance as compared to the theoretical variance expected from random sequencing (i.e., assuming a Poisson sampling process). The average variance over-dispersion drops from 10.5-fold to 3.5-fold after G+C-content normalization. (0.15 MB TIF) [file pcbi.1000988.s006.tif]

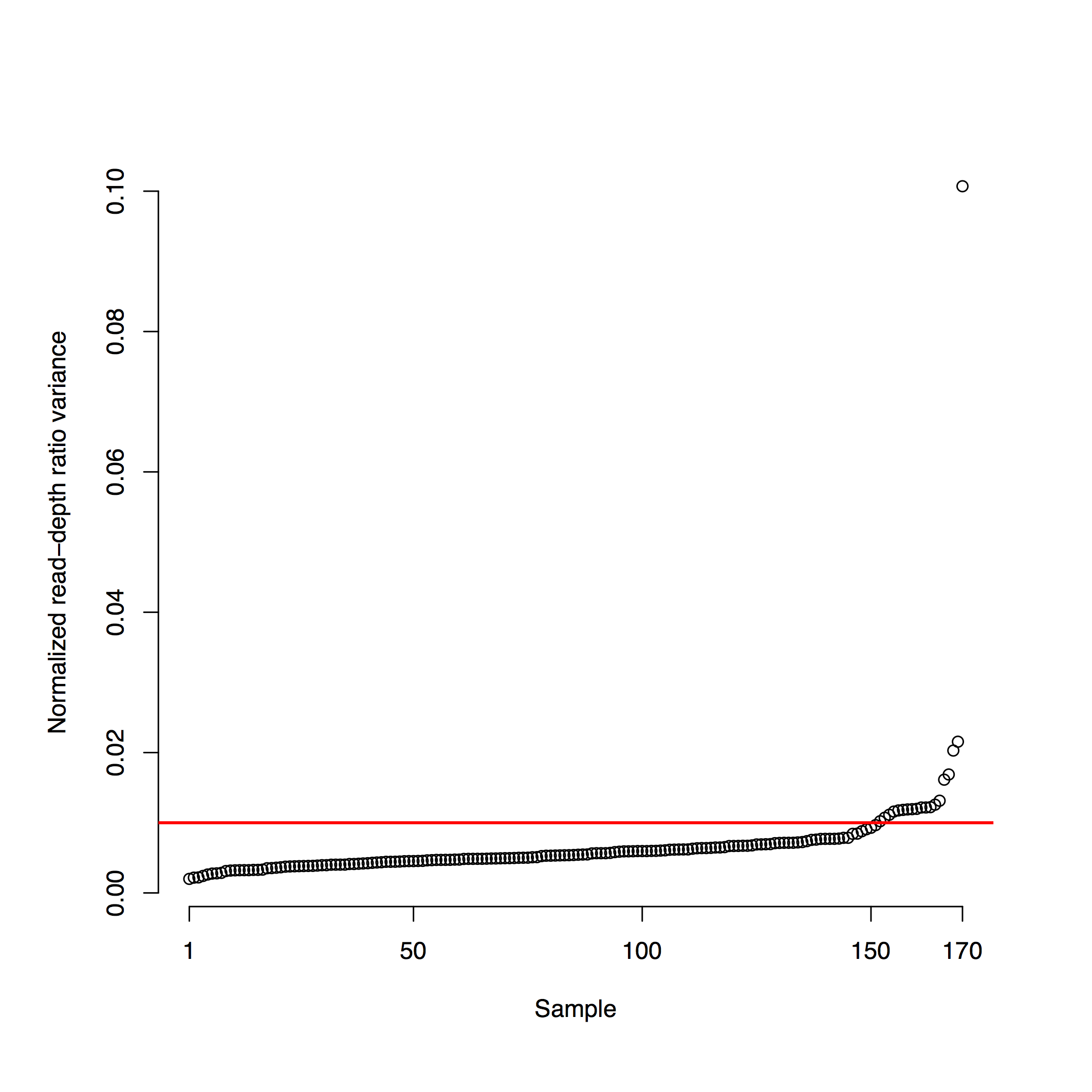

Supplement: Figure S6 — Sample exclusion based on read-depth ratio variance. Samples are sorted on the x-axis by the G+C adjusted variance in the read depth ratio, recorded in the normalization loci (see Materials and Methods and Figure S4). The red line indicates the variance cutoff that was used to exclude samples (0.01); 150 samples were further analyzed in this study. (0.19 MB TIF) [file pcbi.1000988.s007.tif]

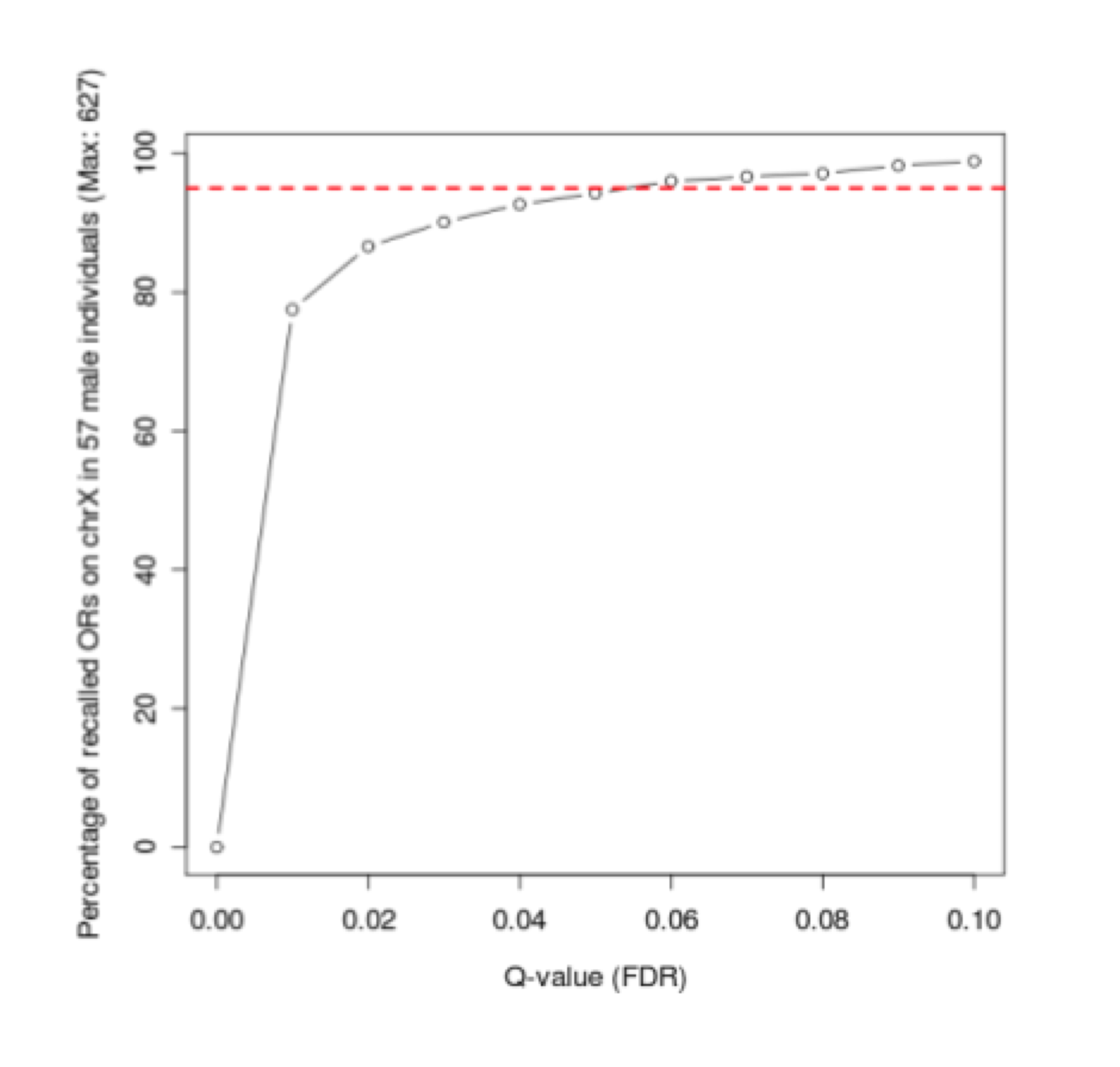

Supplement: Figure S7 — False discovery rate (FDR) threshold in the initial CNV identification step. The figure displays the recall rate for 11 OR loci on the X-chromosome, which were assessed in 57 male samples as a function of different Q-value (FDR) cutoffs in CopySeq's CNV identification step (see Materials and Methods). The recall rate was calculated, assuming that OR loci on chromosome X show no copy-number variation, i.e., all 627 copy-number genotypes in males are expected to display a copy-number genotype of ‘1’ (whereas copy-number genotypes of ‘2’ are expected in female samples); this assumption makes the X-chromosomal OR loci a reasonable test set for identifying a suitable Q-value threshold. For example, when applying a Q-value threshold of 5%, 96% of all OR loci in male samples were correctly identified in the CNV identification step and assigned a copy-number genotype of ‘1’. Furthermore, CopySeq identified a copy-number genotype of ‘2’ in all loci in 93 female samples. (0.34 MB TIF) [file pcbi.1000988.s008.tif]

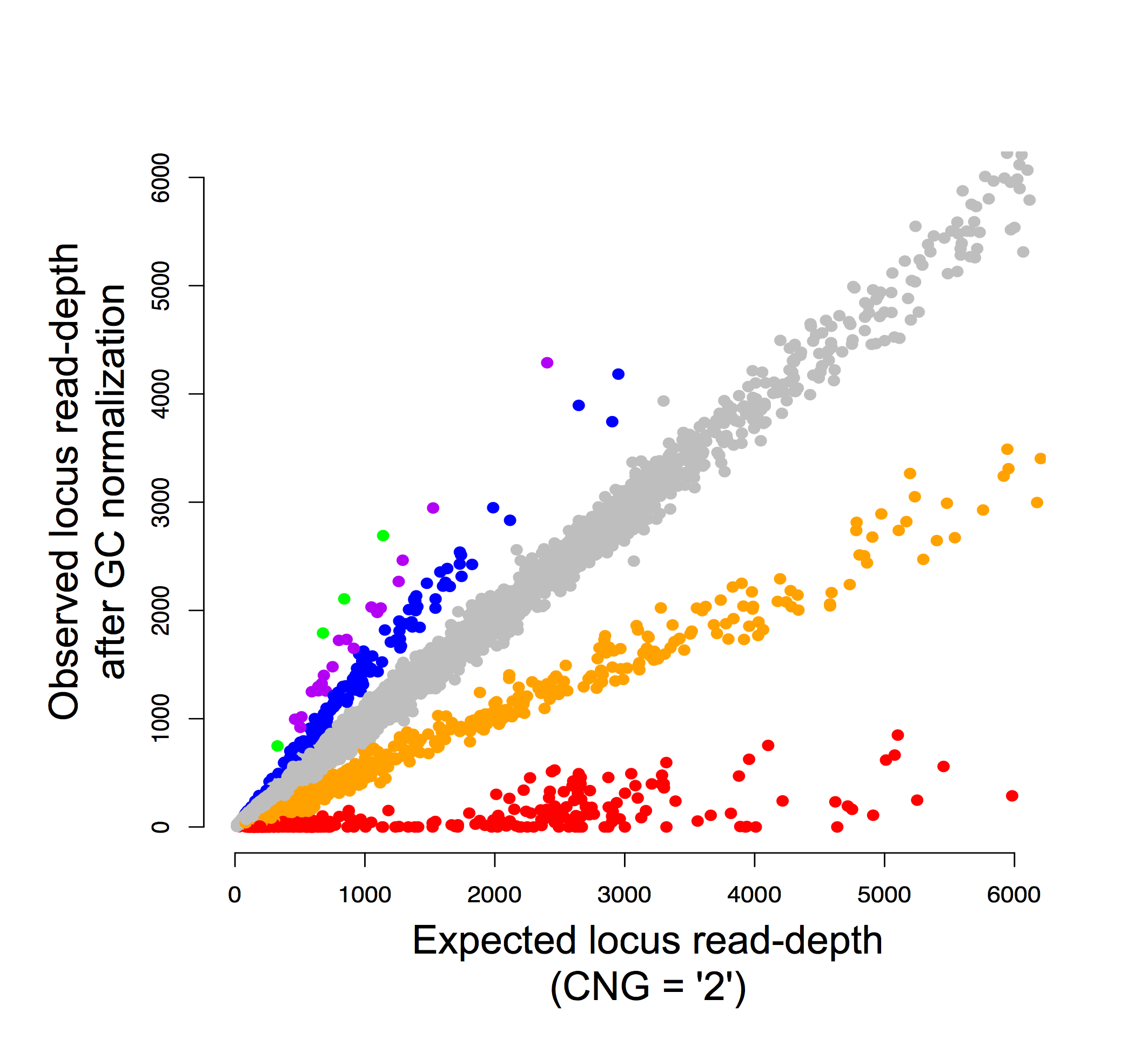

Supplement: Figure S8 — Read-depth distribution in benchmark chromosome 1 CNV set and 150 individuals. Distribution of locus-specific read-depth measurements in the 99 chromosome 1 test loci and 150 individuals. As in Figure 3, the displayed points relate the G+C-adjusted read-depth to the expected read-depth (i.e., copy-number genotype = ‘2’), which is estimated based on the k-mer mappability of a locus and the genomic sequencing coverage of a sample. CopySeq copy-number genotypes are indicated by colors (bottom to top): ‘0’, red; ‘1’, orange; ‘2’, grey; ‘3’, blue; ‘4’, purple; ‘5’, green). (0.31 MB TIF) [file pcbi.1000988.s009.tif]

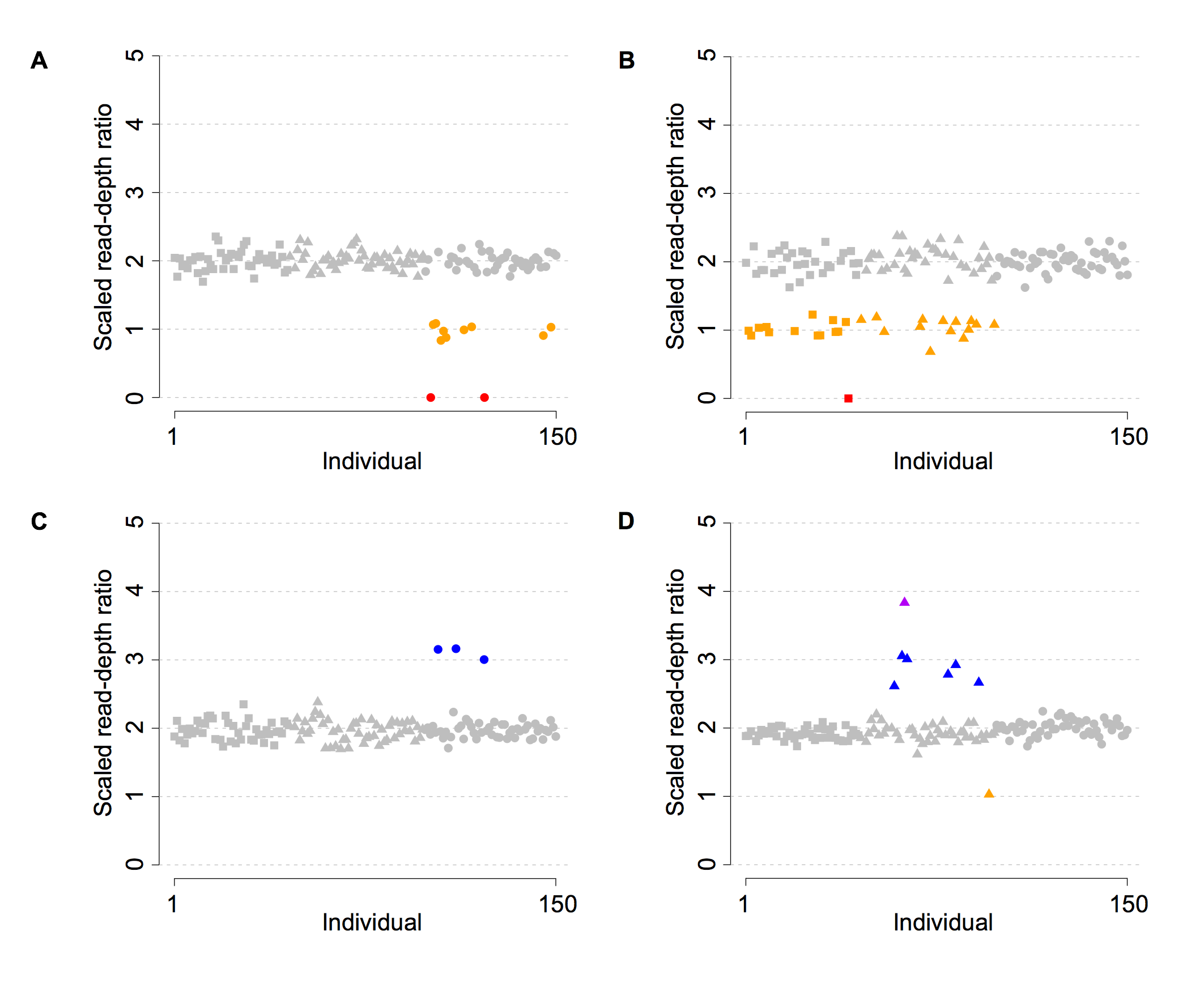

Supplement: Figure S9 — Copy-number genotypes in 150 individuals recorded in the chromosome 1 test set. Copy-number genotyping results for chromosome 1 example CNVs across 150 individuals (see Figure 2 for other examples). Copy-number genotypes inferred by CopySeq are displayed using the same color code as in Figure 2. Samples are sorted by ancestry (square, CEU; triangle, CHB+JPT; circle, YRI). A. chr1:10,293,128–10,300,570; bi-allelic deletion specific for samples with ancestry from Nigeria (YRI). B. chr1:61,886,594–61,890,775; bi-allelic deletion only observed in the CEU and CHB+JPT. C. chr1:235,190,798–235,198,134; bi-allelic duplication observed only in samples of Nigerian ancestry. D. chr1:755,964–799,636; multi-allelic CNV locus, only observed in CHB+JPT samples. (0.25 MB TIF) [file pcbi.1000988.s010.tif]

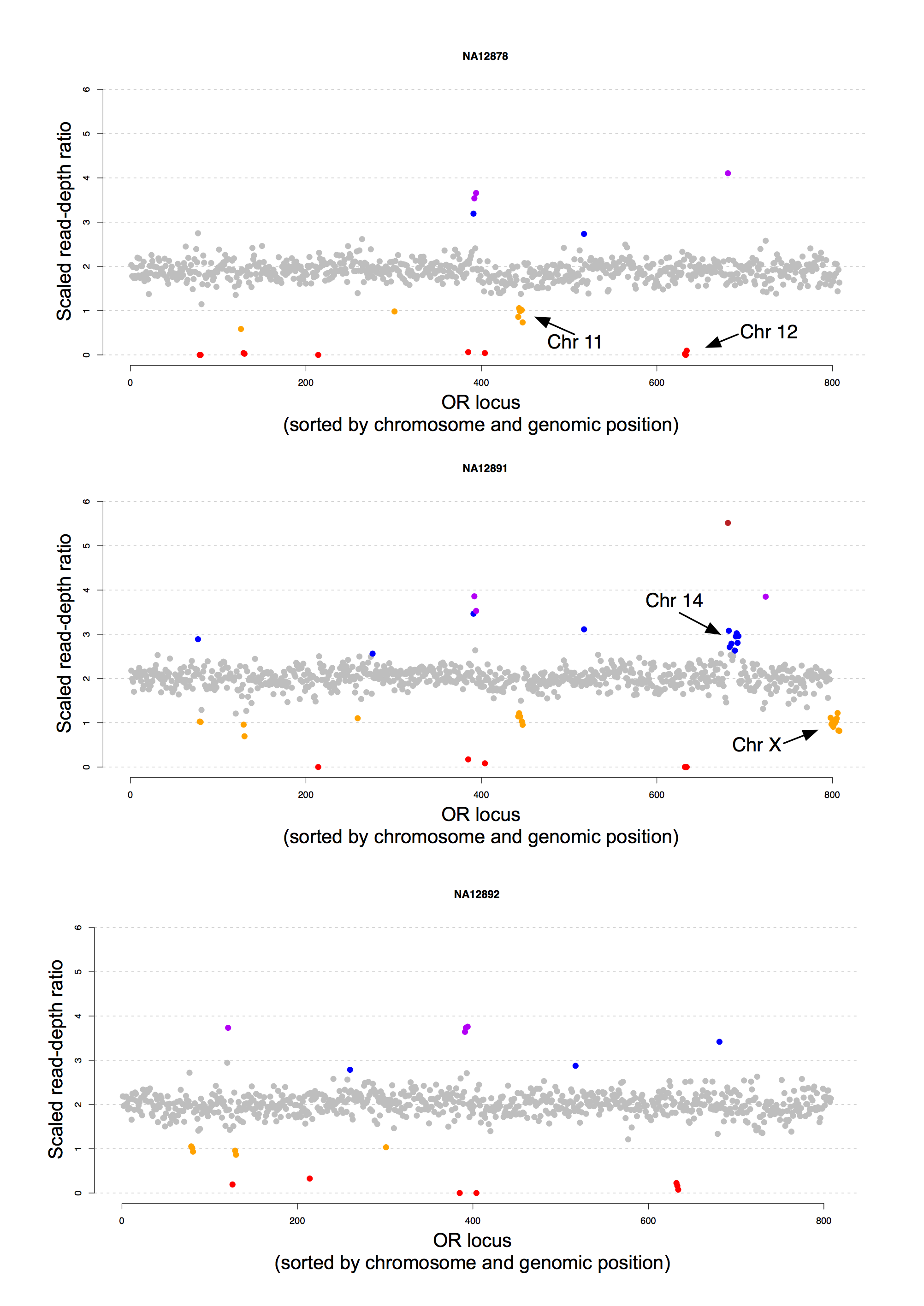

Supplement: Figure S10 — Heritability of locus copy-numbers in all 808 analyzed OR loci. Scaled read-depth and inferred copy-number genotypes of all 808 OR loci analyzed in a CEU parent-offspring trio (from top to bottom: daughter, NA12878; father, NA12891; mother, NA12892). OR loci are sorted according to chromosome and chromosomal coordinate (from left to right). Inferred copy-number genotypes are displayed with the same color code as in Figures 2, 3, 4. Several OR loci of interest are highlighted. Figure 4 displays, in part, the same data, but owing to space limitations focuses on the largest OR cluster, i.e., a cluster of OR genes and pseudogenes on chromosome 11. (0.46 MB TIF) [file pcbi.1000988.s011.tif]

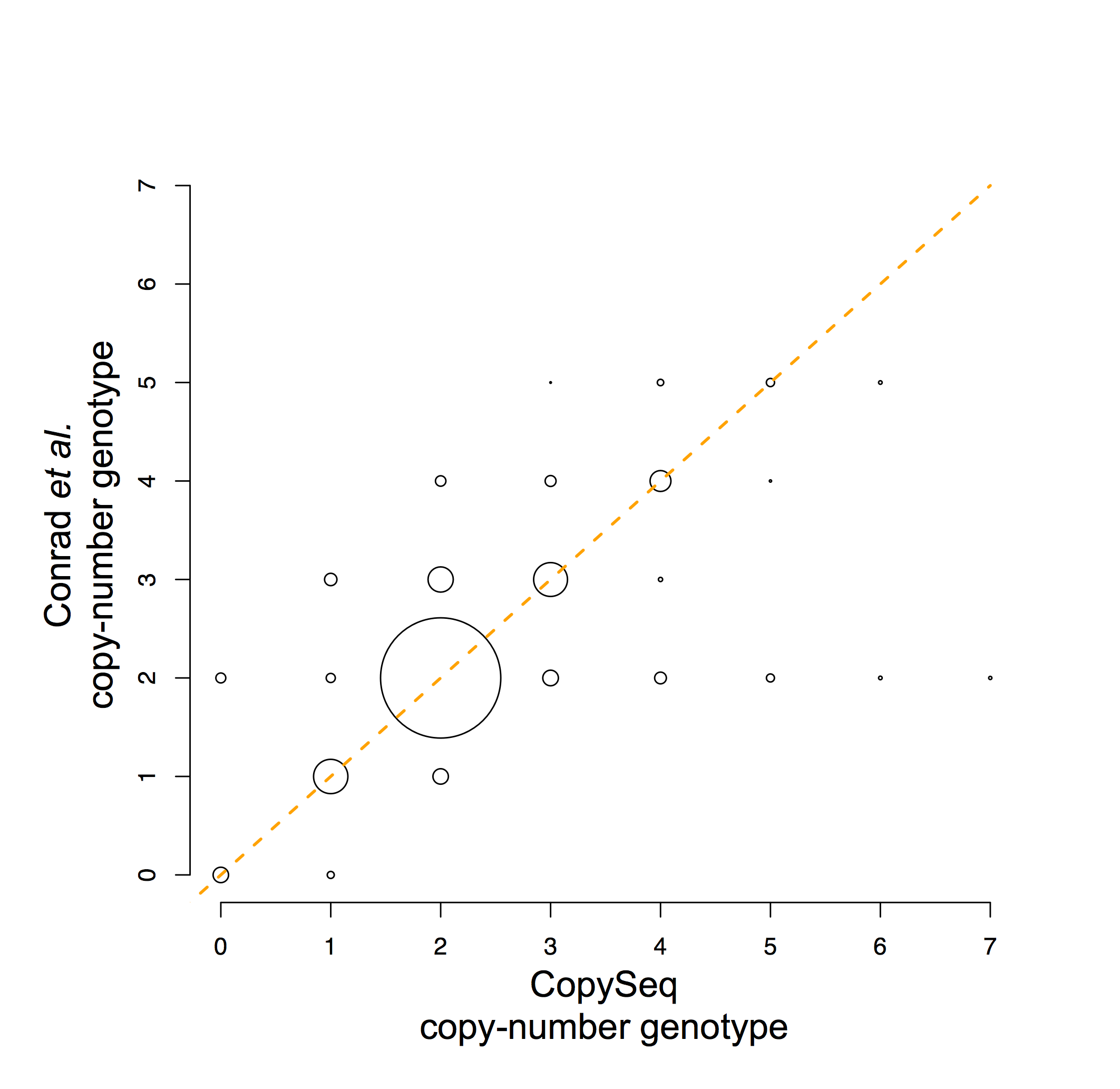

Supplement: Figure S11 — Concordance of CopySeq copy-number genotypes and copy-number genotypes from the microarray-based study by Conrad et al. Comparison of >7,000 CopySeq-based locus copy-number assignments in OR loci with microarray-based copy-number genotypes from Conrad and co-workers [1]. The comparison is based on 51 OR loci, assessed in 149 individuals. Circle size indicates the number of comparisons falling into a certain bin (the largest circle, representing >5,000 copy-number assignments, corresponds to concordant copy-number genotype calls of ‘2’). The orange line denote the function y = x and has been included to facilitate evaluation of the data. The Pearson correlation between the Conrad et al. calls (made with an Agilent oligonucleotide array-CGH platform) and the CopySeq calls was 0.73, slightly weaker than the correlation we measured between CopySeq and Affymetrix SNP 6.0 arrays. This may be due to a higher accuracy of the Affymetrix arrays [2], e.g., owing to the sophisticated probe selection procedure employed; alternatively this may be due to an enrichment of particularly hard to ascertain OR loci (which may display relatively low correlations) in the Conrad et al. study. (0.19 MB TIF) [file pcbi.1000988.s012.tif]

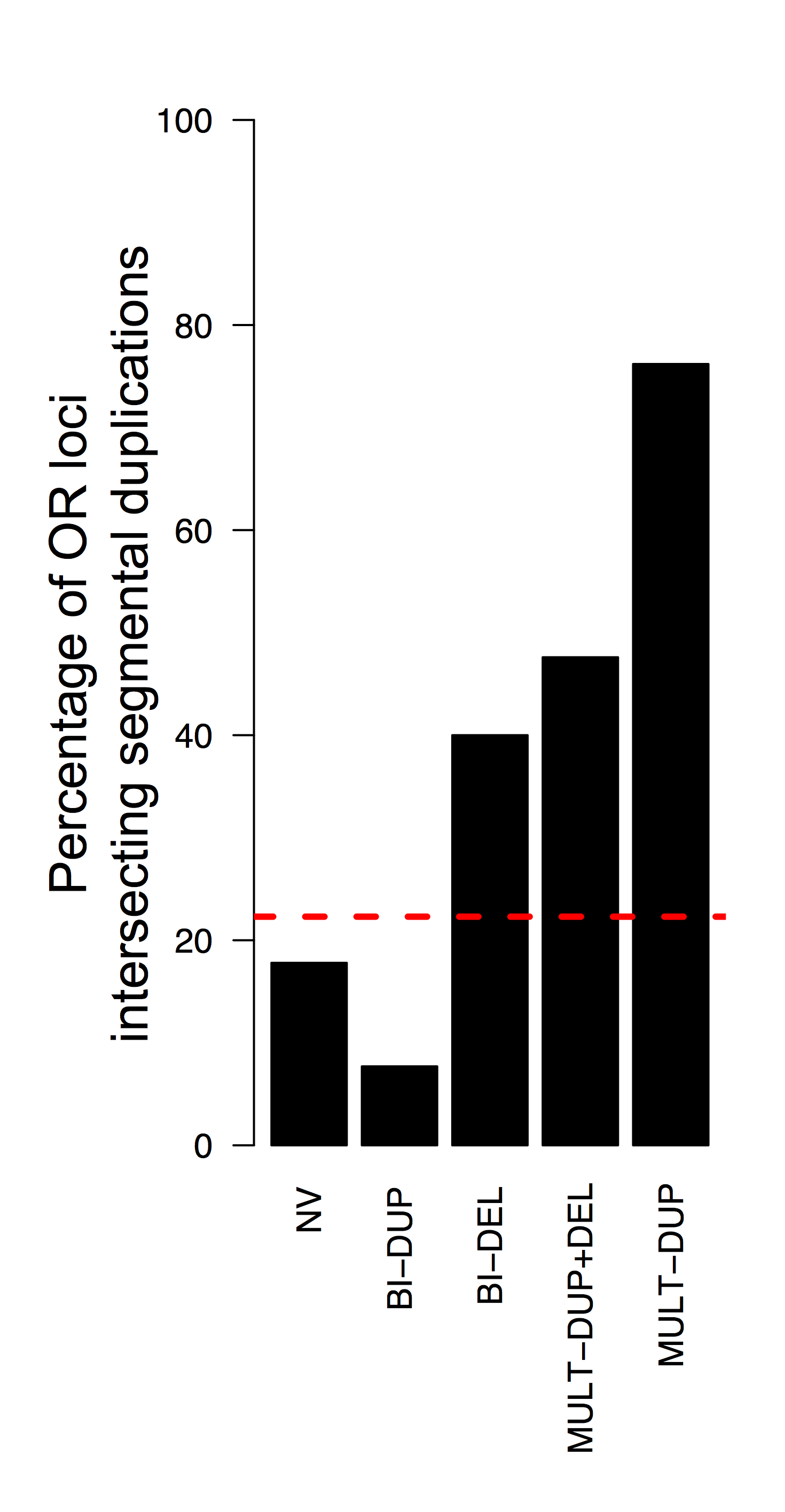

Supplement: Figure S12 — Enrichment of segmental duplications in bi-allelic deletion and multi-allelic OR loci. Fraction of OR loci intersecting annotated SDs (i.e., assessed by a > = 51% overlap of the OR locus with SDs, based on the UCSC genome browser track ‘Segmental Dups’. OR loci were divided into five different classes: NV, non-variable (n = 501); DUP, bi-allelic duplications (n = 130); DEL, bi-allelic deletions (n = 135); DELDUP, multi-allelic OR loci displaying both deletion and duplications (n = 21); MULTDUP, multi-allelic loci that presumably underlie multiple duplications (i.e., loci with at least one copy-number genotype of ‘5’ or higher; n = 21). Red line indicate the average fraction of analyzed autosomal OR loci intersecting with SDs (i.e., 22%). (0.20 MB TIF) [file pcbi.1000988.s013.tif]

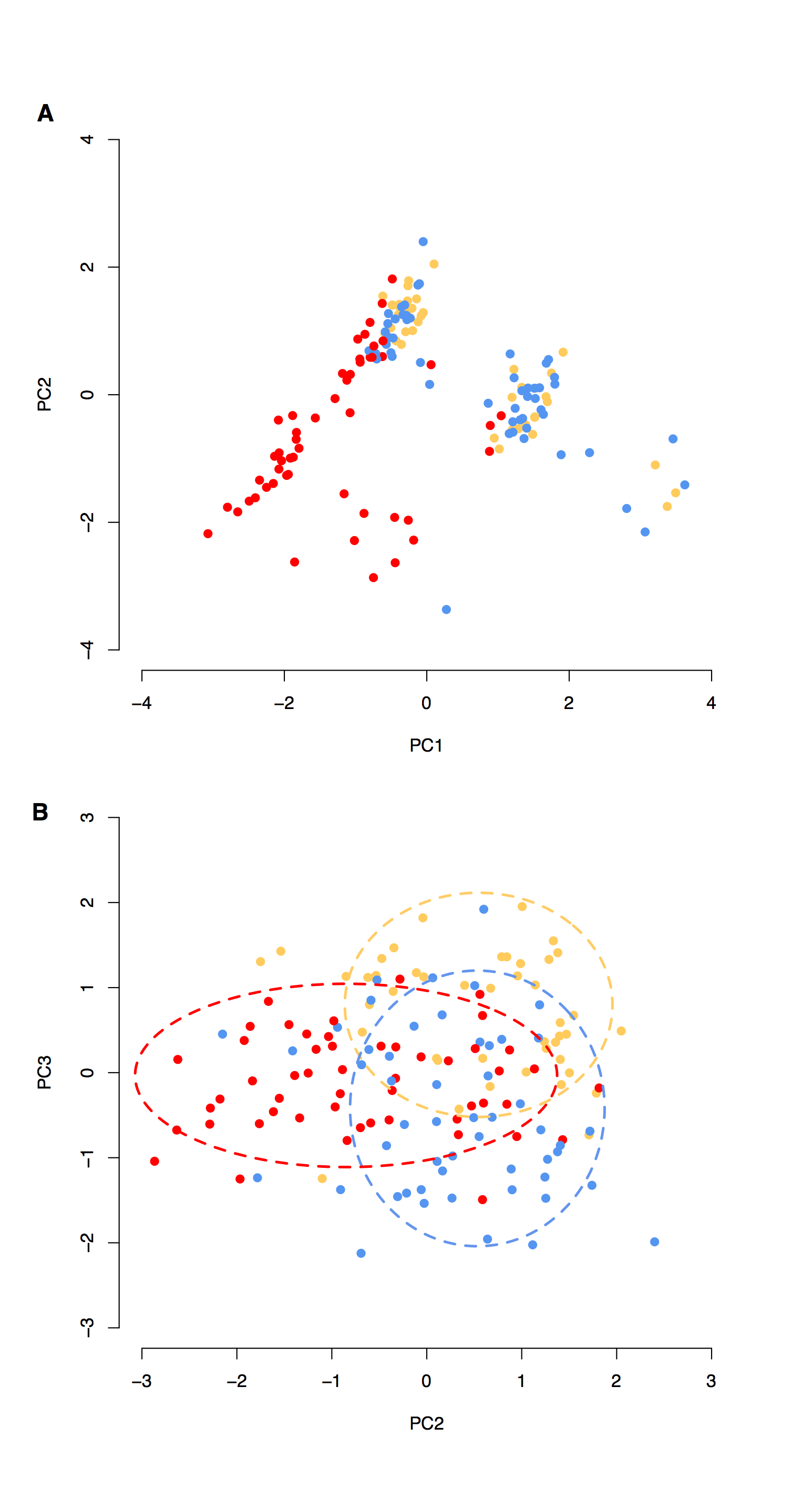

Supplement: Figure S13 — Principal component analysis (PCA) on 265 bi-allelic OR loci and 150 individuals. Population-specific copy-number variation of OR loci displayed by principle component analysis (PCA). PCA analysis was carried out on 265 bi-allelic OR loci in 150 individuals explaining 39% of the total variance (PC1 = 18.9%; PC2 = 12.6%; PC3 = 7.5%). Individuals are colored by ethnic group (YRI, red; CEU, orange; CHB+JPT, blue). A. The first component separates the African (YRI) population from the non-African populations (CEU, CHB, JPT). The three large clusters (PC1: ∼0, ∼1, ∼3) correspond to the copy-number genotypes ‘2’, ‘1’, and ‘0’ (from left to right) of a large, common deletion on chromosome 11 (encompassing OR4C11, OR4P4, OR4S2, OR4V1P, and OR4P1P). B. The second and third principal components facilitate a limited separation between the three populations (highlighted by ellipses), which may be explained by population-specific allele frequencies of a small number of CNVs. (0.24 MB TIF) [file pcbi.1000988.s014.tif]

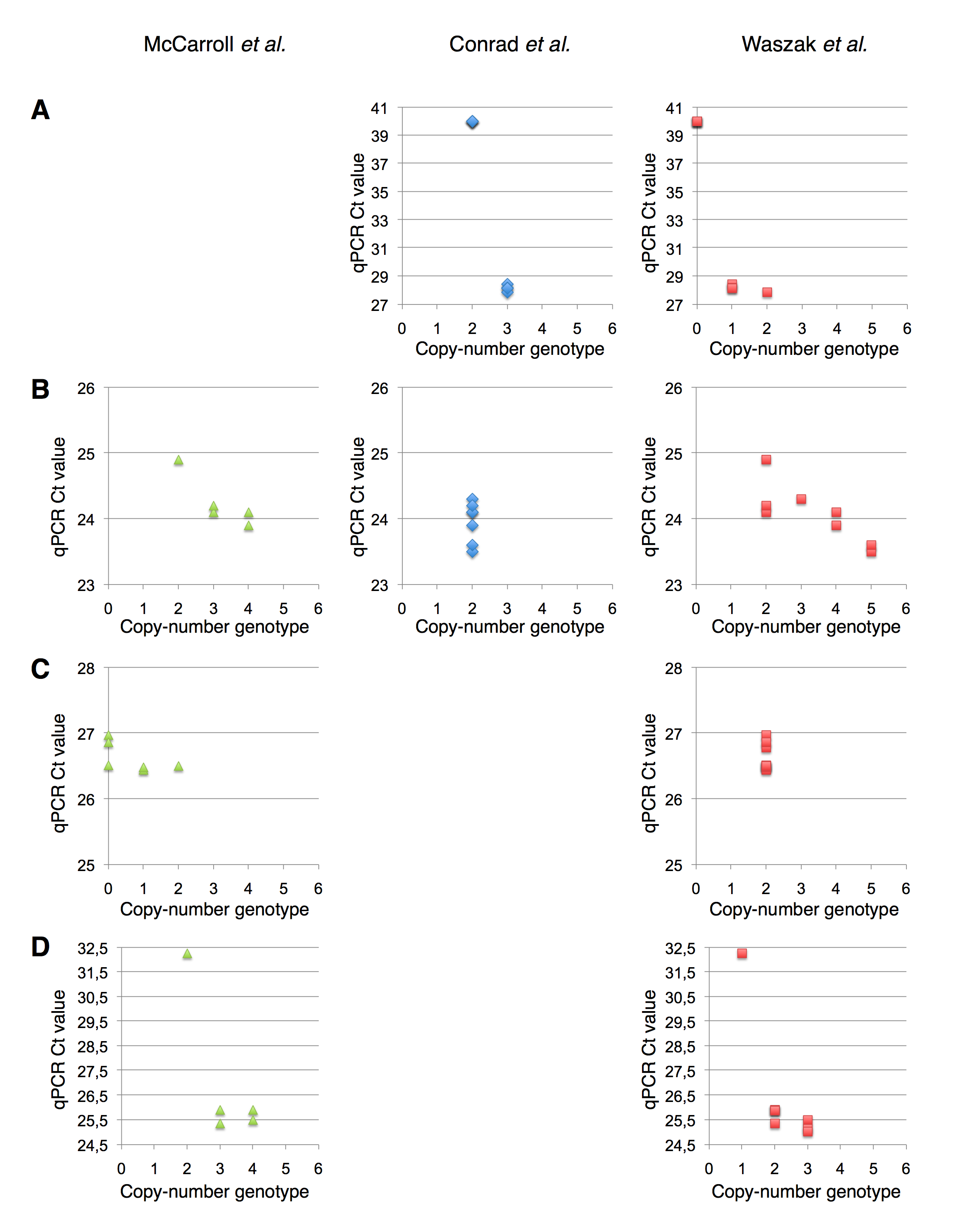

Supplement: Figure S14 — qPCR experiments in OR loci with discordant copy-number genotypes. Four OR loci displaying the least concordance between CopySeq and microarray-based copy-number genotypes (x-axis) were tested by qPCR (y-axis) (Table S14). Each qPCR experiment used triplicates and was repeated at least twice when two primer pairs were used for each locus and three times for loci analysed with one specific primer pair (Detailed Ct, avg delta-Ct and stdev delta-Ct values for all experiments are summarized in Table S14). One representative experiment is shown where experimental qPCR values are compared to copy-number genotype predictions from CopySeq (red), Conrad et al. (blue) or McCarroll et al. (green). The following eight samples were assessed to enable evaluating discrepant calls: NA11881, NA11920, NA11994, NA11995, NA12003, NA12045, NA12155, and NA12716. A. OR4A45P: chr11:48,557,464–48,560,479. Missing data points: CopySeq, none; Conrad et al., none. The absence of a signal (Ct of 40) in qPCR results confirms the copy-number genotype of 0 inferred by CopySeq rather than the copy-number genotype of ‘2’ inferred by Conrad et al. Also, both primer pairs for this locus show the same results and confirm the conclusion made. B. OR11K1P: chr15:19,818,218–19,821,246. Missing data points: CopySeq, none; Conrad et al., NA12003; McCarroll et al., NA11920, NA12045, NA12716. The relative difference of up to 1.55 delta-Ct between DNAs (stdev of delta-Ct between DNAs over all three experiments≤0.09 Ct) and the clustering into several subgroups suggests a multi-allelic CNV, as predicted by CopySeq and McCarroll et al., rather than the absence of a CNV, as predicted by Conrad et al. C. OR5H5P: chr3:99,398,645–99,401,669. Missing data points: CopySeq, none; McCarroll et al., NA11920, NA12045. The almost identical Ct values (maximum difference of 0.23 delta-Ct) in the normal Ct range between all DNAs suggests absence of a CNV, as called by CopySeq, rather than the existence of a homozygous or heter [file pcbi.1000988.s015.tif]

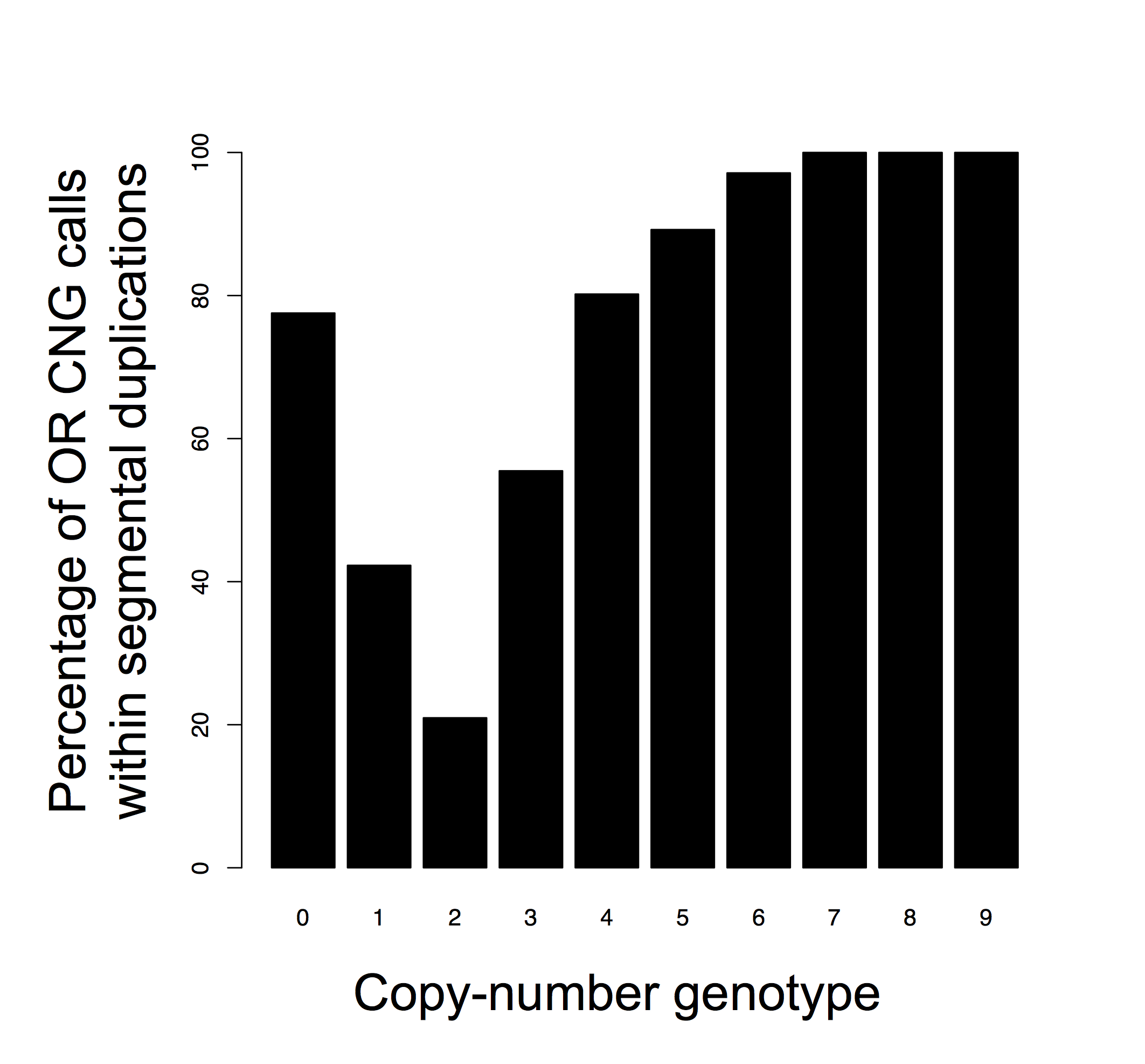

Supplement: Figure S15 — Enrichment of segmental duplications in high copy-number genotype calls. Fraction of 118,650 (791 autosomal loci times 150 samples) OR copy-number genotype calls intersecting annotated SDs (i.e., assessed by requiring a > = 51% overlap of the OR locus with SDs, based on the UCSC genome browser track ‘Segmental Dups’) (0.24 MB TIF) [file pcbi.1000988.s016.tif]

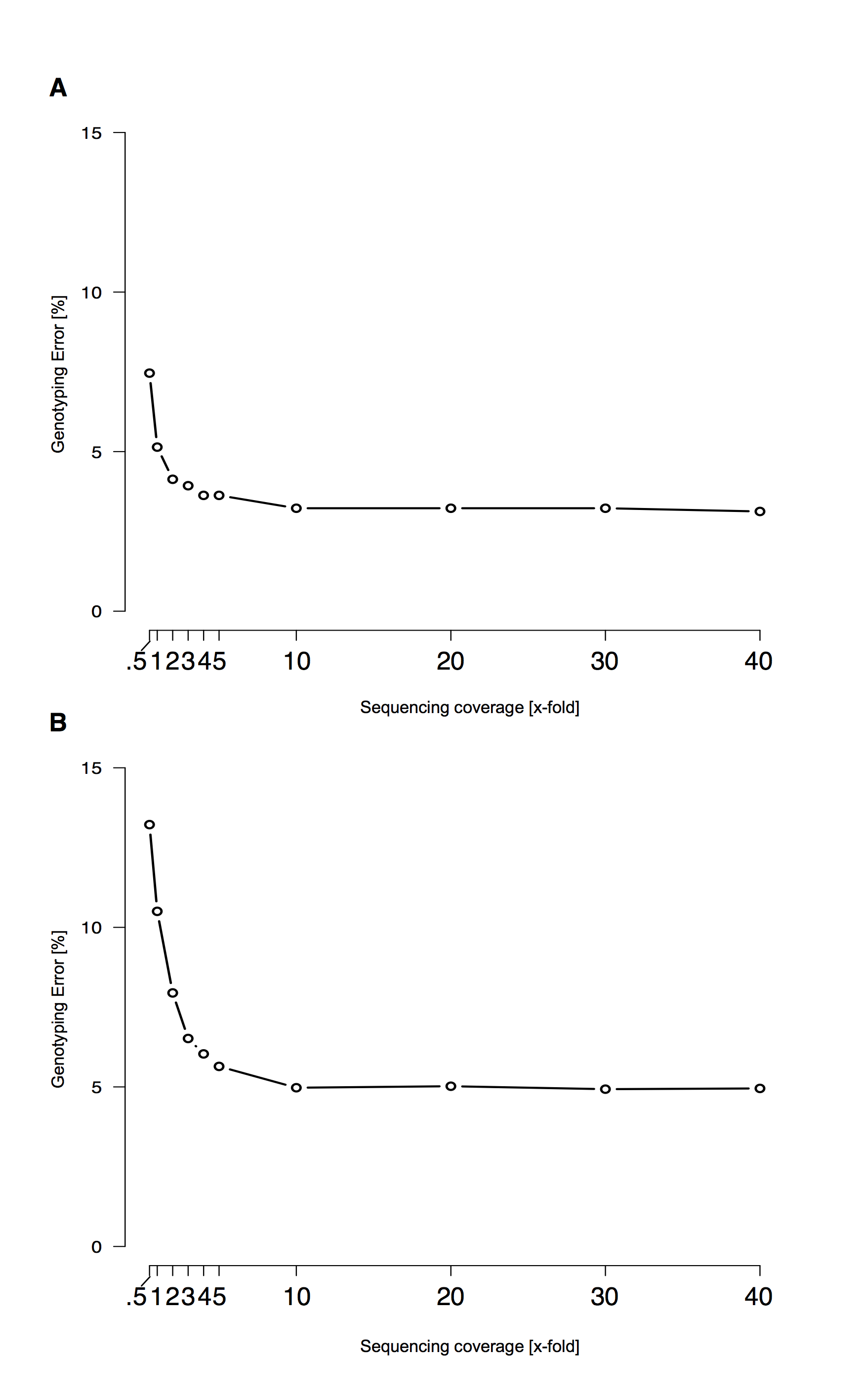

Supplement: Figure S16 — Effect of sequencing coverage on genotyping error rate. Ten different coverages were generated by down-sampling DNA reads from the published NA18507 genome [7]. The following sequencing coverages were used: 0.5×, 1×, 2×, 3×, 4×, 5×, 10×, 20×, 30×, and 40×. CopySeq was applied on these read sets using default parameters (with requested LOD score >0). We compared CopySeq calls to two genome-wide genotype sets for NA18507, i.e., the sets published by McCarroll et al. [2] (A) and Conrad et al. [1] (B). CNV loci intersecting SDs were excluded to circumvent differences in the interpretation of locus-specific copy numbers between platforms in these regions. Genotyping error rates were calculated as #false genotypes/(#true genotypes+#false genotypes). (0.18 MB TIF) [file pcbi.1000988.s017.tif]

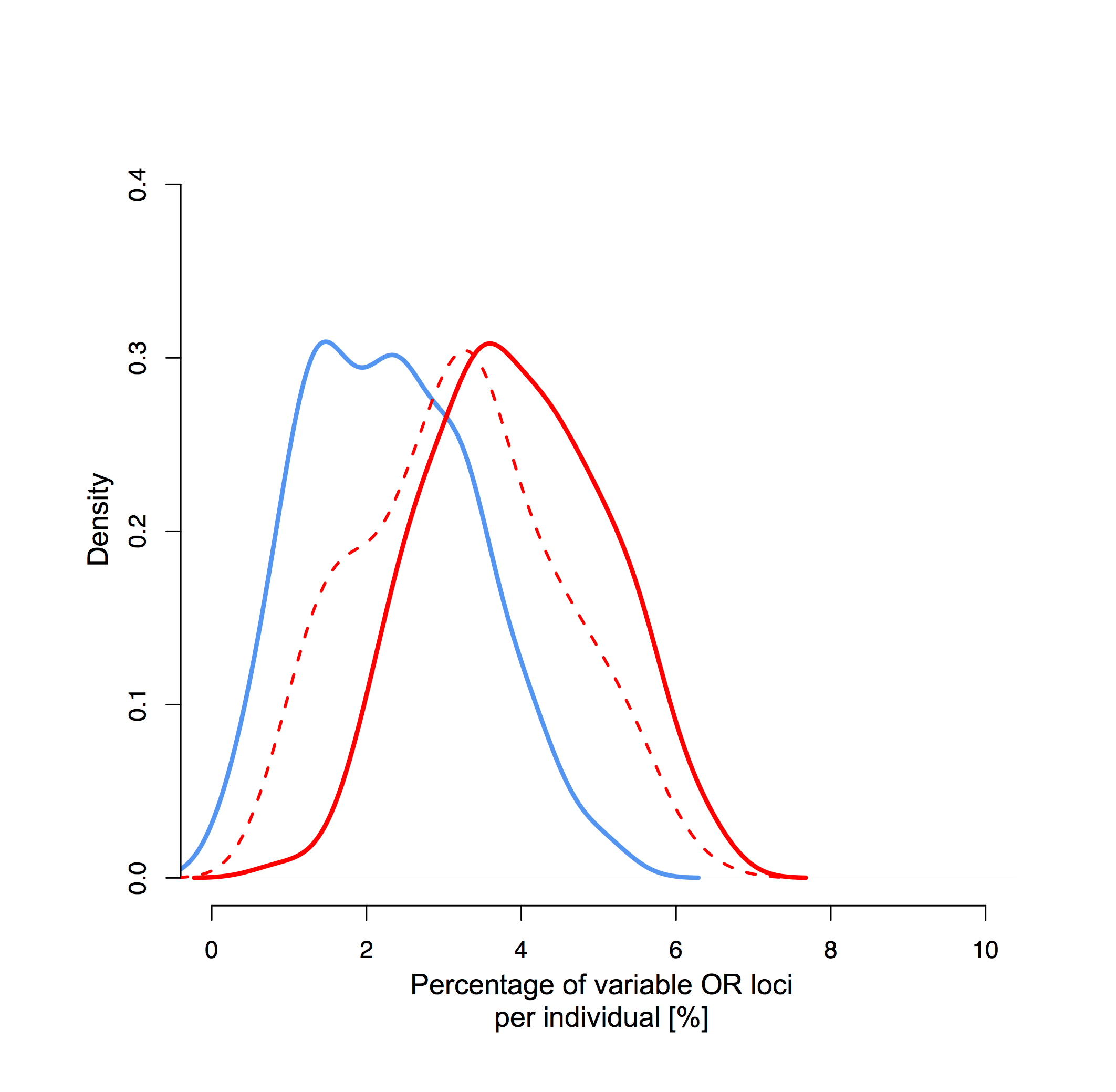

Supplement: Figure S17 — Distribution of variable OR loci within 150 individuals. The figure shows the percentage distribution of variable OR loci per individual relative to the reference genome. Blue solid line: OR genes; red solid line: OR pseudogenes; red dotted line: OR pseudogenes, excluding the CNV-enriched OR7E subfamily. (0.22 MB TIF) [file pcbi.1000988.s018.tif]

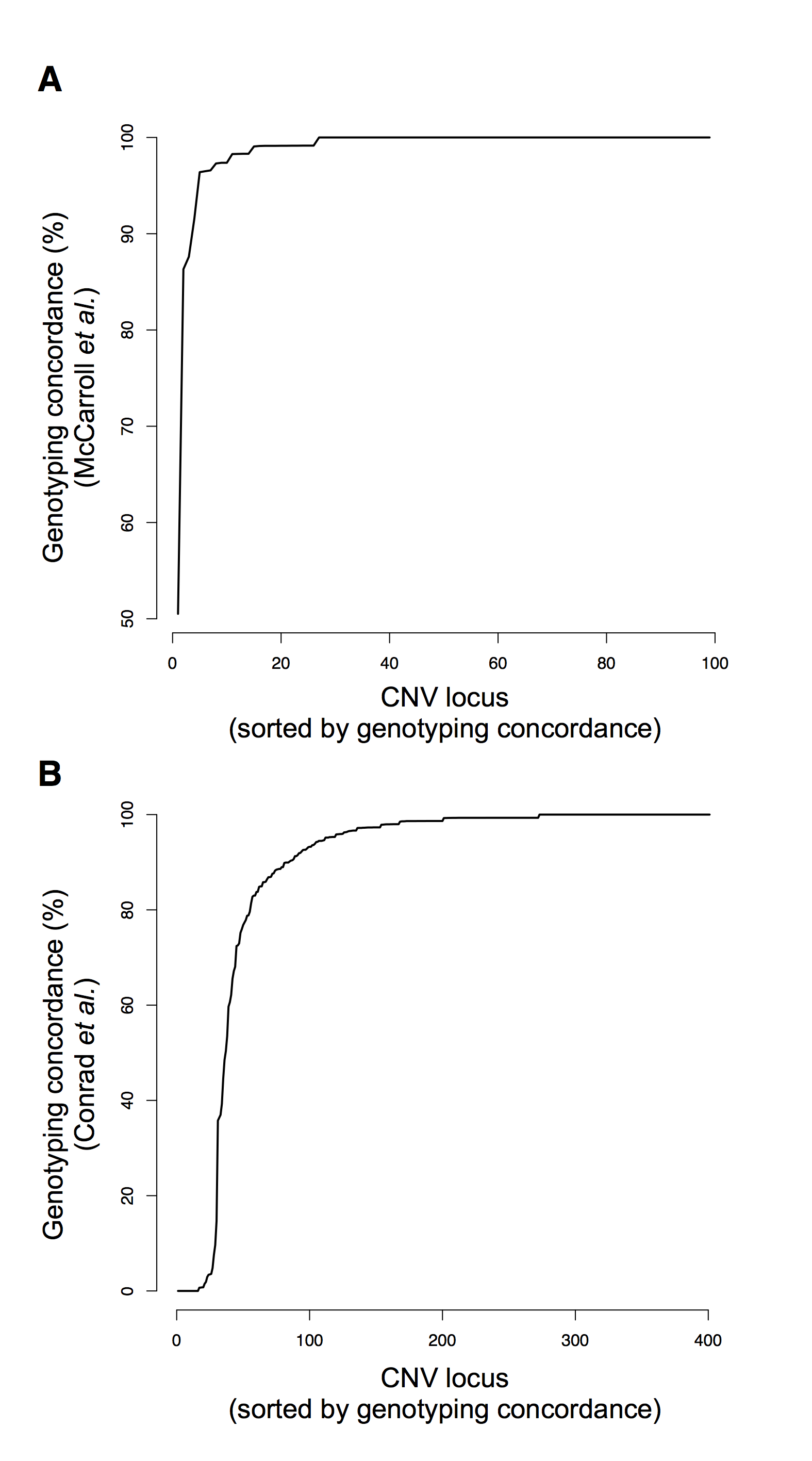

Supplement: Figure S18 — Locus-specific genotyping concordance for 500 benchmark CNVs on chromosome 1. The plots show the locus-specific genotyping concordance for 99 CNV loci on chromosome 1 taken from McCarroll et al. (A) and 401 CNV loci on chromosome 1 taken from Conrad et al. (B). Values are sorted by increasing concordance. While the vast majority of loci showed high concordance, a small subset of loci displayed low concordance (in the Conrad et al. comparison the latter were slightly enriched for small or SD-enriched CNV loci), implying genotyping errors by CopySeq or the respective microarray-based platforms. (0.25 MB TIF) [file pcbi.1000988.s019.tif]

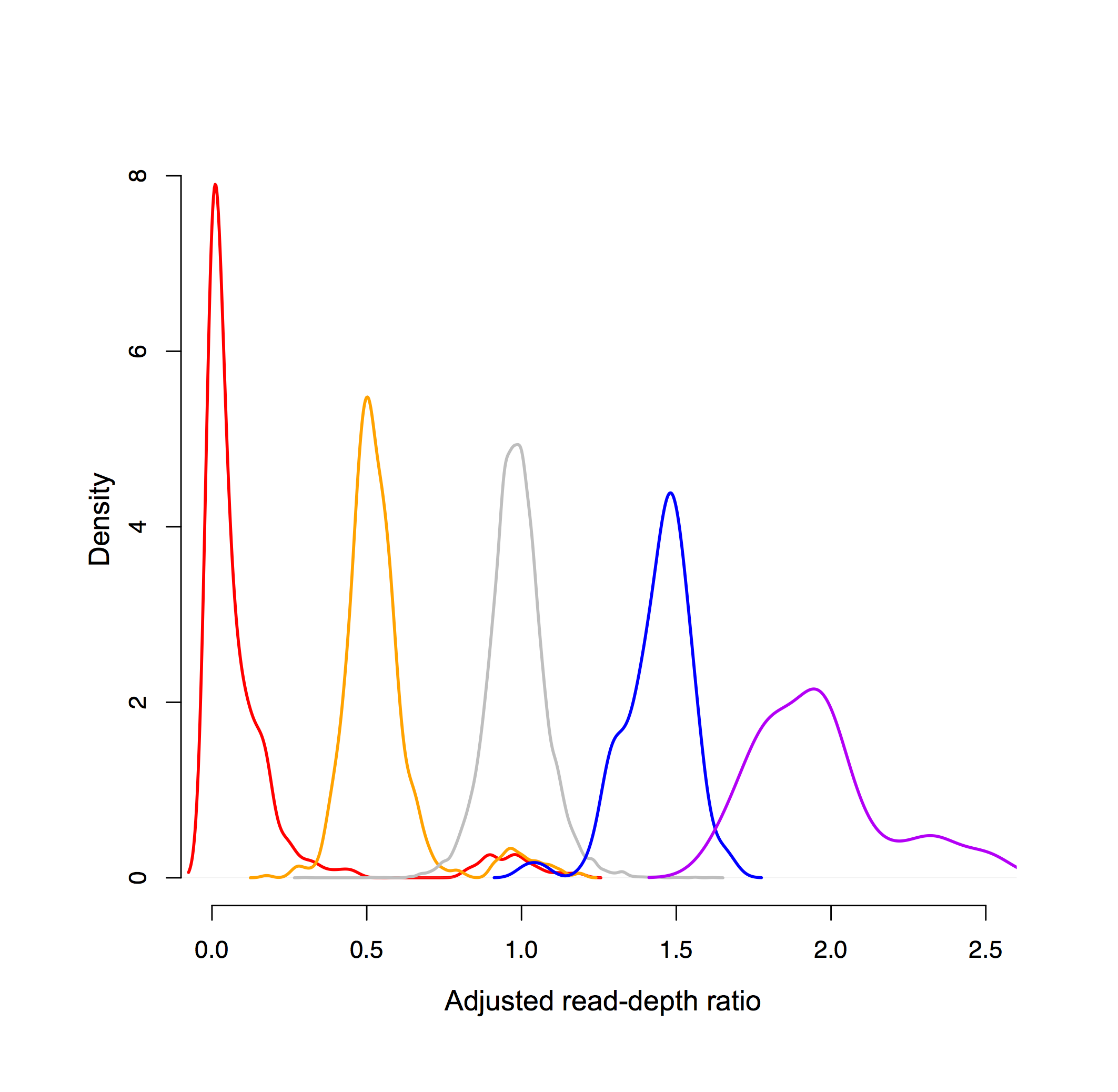

Supplement: Figure S19 — Approximately linear dependency between locus copy-number and read-depth ratio. This figure shows the distribution of adjusted read-depth ratios for 99 CNV loci on chromosome 1 among 118 individuals for which we had prior information in terms of copy-number genotype assignments [2], i.e., individual densities were generated by using the previously published genotype information and grouping our read-depth data according to the genotype assignments in [2]. Copy-number densities are indicated by colors (left to right): ‘0’, red; ‘1’, orange; ‘2’, grey; ‘3’, blue; ‘4’, purple. Thus, for example, all loci classified in [2] having CNG = ‘0’ appear in the density plot in the red density class. The number of data points per density class is: ‘0’ = 322, ‘1’ = 888, ‘2’ = 10,184, ‘3’ = 100, ‘4’ = 21. The plot shows that the expected read-depth ratio mean of a copy-number genotype class scales approximately linearly with copy-number. The mean read-depth ratio values for the copy-number genotype classes (i.e., ‘0’ = 0.11, ‘1’ = 0.54, ‘2’ = 0.99, ‘3’ = 1.44, ‘4’ = 1.96) show good correspondence with the theoretical expected values (‘0’ = 0, ‘1’ = 0.5, ‘2’ = 1, ‘3’ = 1.5, and ‘4’ = 2). Of note, this analysis is obviously heavily depending on the genotype assignments made in [2]. (0.25 MB TIF) [file pcbi.1000988.s020.tif]
