# Supplementary material for: Systematic Inference of Copy-Number Genotypes from Personal Genome Sequencing Data Reveals Extensive Olfactory Receptor Gene Content Diversity
Source: PLoS Comput Biol. 2010 Nov 11;6(11):e1000988. doi: 10.1371/journal.pcbi.1000988 (PMC2978733; doi:10.1371/journal.pcbi.1000988)
Supplement: Table S2 — Genotype concordance with SNP array-based calls on the chromosome 1 test set. (0.03 MB DOC) [file pcbi.1000988.s022.doc]

**Table S2. Genotype concordance with SNP array-based calls on the chromosome 1 test set.**

| **Mode** | **# concordant genotypes** | **# discordant genotypes** | **Genotyping concordance** |
| --- | --- | --- | --- |
| CNG | 11,369 | 146 | 98.7 |
| CNG – PEM | 681 | 3 | 99.6 |
| CNG – BJA | 1,018 | 3 | 99.7 |

Displayed are values for the genotyping concordance, *i.e.,* the number of copy-number genotypes concordant with a previously published microarray-based study [2] divided by total number of copy-number genotypes. Genotype concordance was determined running CopySeq with different modes: CNG, genotype concordance was calculated applying the copy-number genotyping functionality of CopySeq; CNG – PEM, CopySeq was applied with PEM-based boundary redefinition; CNG – BJA, CopySeq was applied with BJA-based boundary redefinition.
