# Supplementary material for: Systematic Inference of Copy-Number Genotypes from Personal Genome Sequencing Data Reveals Extensive Olfactory Receptor Gene Content Diversity
Source: PLoS Comput Biol. 2010 Nov 11;6(11):e1000988. doi: 10.1371/journal.pcbi.1000988 (PMC2978733; doi:10.1371/journal.pcbi.1000988)
Supplement: Table S6 — Outcomes copy-number genotyping on chromosome 1 benchmark set. (0.04 MB DOC) [file pcbi.1000988.s026.doc]

Table S6. Outcomes copy-number genotyping on chromosome 1 benchmark set.

| Copy-number genotype | TP | FP | TN | FN | Sensitivity (%) | Specificity (%) | PPV  (%) |
| --- | --- | --- | --- | --- | --- | --- | --- |
| 0 | 283 | 0 | 11,193 | 39 | 87.9 | 100 | 100 |
| 1 | 820 | 30 | 10,597 | 68 | 92.3 | 99.7 | 96.5 |
| 2 | 10,154 | 91 | 1,240 | 30 | 99.7 | 93.2 | 99.1 |
| 3 | 96 | 22 | 11,393 | 4 | 96.0 | 99.8 | 81.4 |
| 4 | 16 | 0 | 11,494 | 5 | 76.2 | 100 | 100 |
| 5 | 0 | 3 | 11,512 | 0 | N/A | 100 | 0.0 |
| The table presents CopySeq results, summarizing all copy-number genotypes on the chromosome 1 benchmark set. TP, true positive; FP, false positive; TN, true negative; FN, false negative. Sensitivity = TP / (TP + FN); Specificity = TN / (FP + TN); positive predictive value (PPV) = TP / (TP + FP). Decisions as to whether genotypes represented ‘true’ or ‘false’ positives were made based on array-based calls from McCarroll and co-workers [2], which were here conservatively interpreted as a gold standard. TP, ‘true positive’, or CNV was identified by CopySeq and copy-number genotypes were concordant with McCarroll *et al.;* FP, ‘false positive’, or CNV was identified by CopySeq, but copy-number genotypes were discordant with McCarroll *et al.*; TN, ‘true negative’, or both CopySeq and McCarroll *et al.* inferred no CNV (i.e., both concordantly identified a homozygous reference allele with copy-number assignment = ‘2’); FN, ‘false negative’, or McCarroll *et al.* identified a CNV, but CopySeq identified a homozygous reference allele. | | | | | | | |
