# Supplementary material for: Systematic Inference of Copy-Number Genotypes from Personal Genome Sequencing Data Reveals Extensive Olfactory Receptor Gene Content Diversity
Source: PLoS Comput Biol. 2010 Nov 11;6(11):e1000988. doi: 10.1371/journal.pcbi.1000988 (PMC2978733; doi:10.1371/journal.pcbi.1000988)
Supplement: Table S7 — High-confidence copy-number genotyping on benchmark set. (0.04 MB DOC) [file pcbi.1000988.s027.doc]

| **Table S7. High-confidence copy-number genotyping on benchmark set.** | | | | | | | |
| --- | --- | --- | --- | --- | --- | --- | --- |
| Copy-number genotype | TP | FP | TN | FN | Sensitivity (%) | Specificity (%) | PPV  (%) |
| 0 | 277 | 0 | 11,160 | 35 | 88.8 | 100 | 100 |
| 1 | 816 | 20 | 10,568 | 68 | 92.3 | 99.8 | 97.6 |
| 2 | 10,140 | 91 | 1,223 | 18 | 99.8 | 93.1 | 99.1 |
| 3 | 95 | 15 | 11,358 | 4 | 96.0 | 99.9 | 86.4 |
| 4 | 15 | 0 | 11,453 | 4 | 78.9 | 100 | 100 |
| 5 | 0 | 3 | 11,469 | 0 | N/A | 100 | 0.0 |
| The table presents CopySeq results for copy-number genotypes made at high confidence (i.e., with LOD-score ≥ 2.0) on the chromosome 1 benchmark set. TP, FP, TN, and FN are defined as in Table S6. | | | | | | | |
