# Supplementary material for: Systematic Inference of Copy-Number Genotypes from Personal Genome Sequencing Data Reveals Extensive Olfactory Receptor Gene Content Diversity
Source: PLoS Comput Biol. 2010 Nov 11;6(11):e1000988. doi: 10.1371/journal.pcbi.1000988 (PMC2978733; doi:10.1371/journal.pcbi.1000988)
Supplement: Table S8 — Outcomes CNV identification with CopySeq on chromosome 1 benchmark set. (0.04 MB DOC) [file pcbi.1000988.s028.doc]

Table S8. Outcomes CNV identification with CopySeq on chromosome 1 benchmark set.

| Type of CNV | TP | FP | TN | FN | Sensitivity (%) | Specificity (%) | PPV  (%) |
| --- | --- | --- | --- | --- | --- | --- | --- |
| Deletion | 1,109 | 4 | 10,272 | 87 | 92.7 | 100 | 99.6 |
| Duplication | 114 | 14 | 11,340 | 4 | 96.6 | 99.9 | 89.1 |
| All | 1,223 | 18 | 10,140 | 91 | 93.1 | 99.8 | 98.5 |

The table presents CopySeq results for copy-number genotypes on the chromosome 1 benchmark set (LOD-score ≥ 2.0). Deletions are CNVs for which CopySeq inferred copy-numbers of 0 or 1. Duplications are CNVs for which CopySeq inferred copy-numbers of ≥3. TP, FP, TN, and FN are defined as in Table S6.
