# Supplementary material for: Systematic Inference of Copy-Number Genotypes from Personal Genome Sequencing Data Reveals Extensive Olfactory Receptor Gene Content Diversity
Source: PLoS Comput Biol. 2010 Nov 11;6(11):e1000988. doi: 10.1371/journal.pcbi.1000988 (PMC2978733; doi:10.1371/journal.pcbi.1000988)
Supplement: Table S9 — Outcomes copy-number genotyping integrated with paired-end mapping on chromosome 1 benchmark set. (0.04 MB DOC) [file pcbi.1000988.s029.doc]

Table S9. Outcomes copy-number genotyping integrated with paired-end mapping on chromosome 1 benchmark set

| Copy-number genotype | TP | FP | TN | FN | Sensitivity  (%) | Specificity (%) | PPV  (%) |
| --- | --- | --- | --- | --- | --- | --- | --- |
| 0 | 99 | 0 | 585 | 0 | 100 | 100 | 100 |
| 1 | 115 | 0 | 569 | 0 | 100 | 100 | 100 |
| 2 | 464 | 2 | 218 | 0 | 100 | 99.1 | 99.6 |
| 3 | 3 | 1 | 678 | 2 | 60.0 | 99.9 | 75.0 |
| 4 | 0 | 0 | 773 | 1 | 0.0 | 100 | N/A |

The table presents CopySeq results for copy-number genotypes on the chromosome 1 benchmark set (LOD-score ≥ 2.0). TP, FP, TN, and FN are defined as in Table S6.
