# Supplementary material for: Systematic Inference of Copy-Number Genotypes from Personal Genome Sequencing Data Reveals Extensive Olfactory Receptor Gene Content Diversity
Source: PLoS Comput Biol. 2010 Nov 11;6(11):e1000988. doi: 10.1371/journal.pcbi.1000988 (PMC2978733; doi:10.1371/journal.pcbi.1000988)
Supplement: Table S10 — Outcomes copy-number genotyping integrated with breakpoint junction library analysis on chromosome 1 benchmark set. (0.04 MB DOC) [file pcbi.1000988.s030.doc]

Table S10. Outcomes copy-number genotyping integrated with breakpoint junction library analysis on chromosome 1 benchmark set

| Copy-number genotype | TP | FP | TN | FN | Sensitivity  (%) | Specificity (%) | PPV  (%) |
| --- | --- | --- | --- | --- | --- | --- | --- |
| 0 | 115 | 0 | 906 | 0 | 100 | 100 | 100 |
| 1 | 236 | 0 | 784 | 1 | 99.6 | 100 | 100 |
| 2 | 667 | 1 | 351 | 2 | 99.7 | 99.7 | 99.9 |
| 3 | 0 | 2 | 1,019 | 0 | N/A | 99.8 | 0.0 |

The table presents CopySeq results for copy-number genotypes on the chromosome 1 benchmark set (LOD-score ≥ 2.0). TP, FP, TN, and FN are defined as in Table S6.
