# Supplementary material for: Systematic Inference of Copy-Number Genotypes from Personal Genome Sequencing Data Reveals Extensive Olfactory Receptor Gene Content Diversity
Source: PLoS Comput Biol. 2010 Nov 11;6(11):e1000988. doi: 10.1371/journal.pcbi.1000988 (PMC2978733; doi:10.1371/journal.pcbi.1000988)
Supplement: Table S11 — Summary of detected CNVs affecting OR loci. (0.03 MB DOC) [file pcbi.1000988.s031.doc]

Table S11. Summary detected CNVs affecting OR loci.

|  | **Intact genes** | **Pseudogenes**  **(w/o 7E subfamily)** | **Pseudogenes**  **(only 7E subfamily)** | **Total** |
| --- | --- | --- | --- | --- |
| All OR loci | 387 | 379 | 85 | 851 |
| All mappable loci | 365 | 362 | 81 | 808 |
| All variable loci | 130 | 137 | 40 | 307 |
| Bi-allelic loci (duplications) | 67 | 59 | 4 | 130 |
| Bi-allelic loci (deletions) | 44 | 60 | 31 | 135 |
| Multi-allelic loci | 19 | 18 | 5 | 42 |
