# Supplementary material for: Systematic Inference of Copy-Number Genotypes from Personal Genome Sequencing Data Reveals Extensive Olfactory Receptor Gene Content Diversity
Source: PLoS Comput Biol. 2010 Nov 11;6(11):e1000988. doi: 10.1371/journal.pcbi.1000988 (PMC2978733; doi:10.1371/journal.pcbi.1000988)
Supplement: Table S15 — Copy-number genotyping concordance between CopySeq and custom Agilent CGH arrays (Conrad et al.) (0.04 MB DOC) [file pcbi.1000988.s035.doc]

Table S15. Copy-number genotyping concordance between CopySeq and custom Agilent CGH arrays

| **CNV size cutoff [bp]** | **Total number of loci** | **Copy-number genotyping concordance [%]** |
| --- | --- | --- |
| All (no size cutoff) | 401 | 89.1 |
| ≥1,000 | 345 | 88.8 |
| ≥2,000 | 260 | 86.8 |
| ≥3,000 | 207 | 85.6 |
| ≥4,000 | 171 | 83.5 |
| ≥5,000 | 147 | 81.4 |
| ≥6,000 | 116 | 79.1 |
| ≥7,000 | 106 | 77.3 |
| ≥8,000 | 96 | 75.0 |
| ≥9,000 | 80 | 70.5 |
| ≥10,000 | 76 | 70.0 |
| ≥20,000 | 56 | 59.3 |
| ≥40,000 | 44 | 54.2 |
| ≥100,000 | 22 | 46.7 |
