# Supplementary material for: Systematic Inference of Copy-Number Genotypes from Personal Genome Sequencing Data Reveals Extensive Olfactory Receptor Gene Content Diversity
Source: PLoS Comput Biol. 2010 Nov 11;6(11):e1000988. doi: 10.1371/journal.pcbi.1000988 (PMC2978733; doi:10.1371/journal.pcbi.1000988)
Supplement: Table S16 — Copy-number genotyping concordance between CopySeq and custom Agilent CGH arrays (Conrad et al.) in regions that do not intersect with SDs. (0.04 MB DOC) [file pcbi.1000988.s036.doc]

**Table S16. Copy-number genotyping concordance between CopySeq and custom Agilent CGH arrays (Conrad *et al.*)** in regions that do not intersect with SDs

| **CNV size cutoff**  **[bp]** | **Total number of loci  (regions display no SDs)** | **Copy-number genotyping concordance [%]** |
| --- | --- | --- |
| All (no size cutoff) | 339 | 94.8 |
| ≥1,000 | 283 | 95.6 |
| ≥2,000 | 199 | 95.9 |
| ≥3,000 | 149 | 96.7 |
| ≥4,000 | 114 | 97.0 |
| ≥5,000 | 91 | 97.3 |
| ≥6,000 | 63 | 97.7 |
| ≥7,000 | 55 | 97.6 |
| ≥8,000 | 48 | 97.3 |
| ≥9,000 | 35 | 96.9 |
| ≥10,000 | 32 | 96.6 |
| ≥20,000 | 16 | 93.7 |
| ≥40,000 | 7 | 99.8 |
| ≥100,000 | 2 | 100 |
