# Supplementary material for: Systematic Inference of Copy-Number Genotypes from Personal Genome Sequencing Data Reveals Extensive Olfactory Receptor Gene Content Diversity
Source: PLoS Comput Biol. 2010 Nov 11;6(11):e1000988. doi: 10.1371/journal.pcbi.1000988 (PMC2978733; doi:10.1371/journal.pcbi.1000988)
Supplement: Table S17 — Copy-number genotyping concordance between CopySeq and two array platforms in large CNVs intersecting with SDs. (0.04 MB DOC) [file pcbi.1000988.s037.doc]

**Table S17. Copy-number genotyping concordance between CopySeq and two array platforms in large CNVs intersecting with SDs
The CNV loci were filtered in the following way: CNVs >10kb ascertained with Agilent CGH arrays that displayed less than 10% concordance between CopySeq and array-CGH. We next kept all regions (i.e., four in total) for which there was exactly one CNV measured on Affymetrix arrays that either was contained in the CNV measured by array-CGH or showed a reciprocal overlap of 50%. Of note, we found that all four regions intersected with SDs. CopySeq copy-number genotypes are based on Agilent array genomic locus coordinates.**

|  |  |  | **Genotyping concordance** | | |
| --- | --- | --- | --- | --- | --- |
| **Case** | **Genomic coordinates (Agilent)** | **Genomic coordinates (Affymetrix)** | **Agilent- Affymetrix [%]** | **Agilent - CopySeq [%]** | **Affymetrix- CopySeq [%]** |
| I | 1:1,557,744-1,673,566 | 1:1,617,778-1,662,463 | 0.9 | 2.3 | 96.4 |
| II | 1:25,457,812-25,537,782 | 1:25,465,715-25,534,592 | 0 | 0 | 98.3 |
| III | 1:110,016,535-110,046,454 | 1:110,025,907-110,044,476 | 0 | 0 | 54.8 |
| IV | 1:147,300,034-147,726,239 | 1:147,303,148-147,526,040 | 0 | 0 | 99 |
