# Supplementary material for: Systematic Inference of Copy-Number Genotypes from Personal Genome Sequencing Data Reveals Extensive Olfactory Receptor Gene Content Diversity
Source: PLoS Comput Biol. 2010 Nov 11;6(11):e1000988. doi: 10.1371/journal.pcbi.1000988 (PMC2978733; doi:10.1371/journal.pcbi.1000988)
Supplement: Table S18 — Comparison of CopySeq copy-number genotyping with the read-counting approach by Alkan and coworkers. (0.05 MB DOC) [file pcbi.1000988.s038.doc]

**Table S18**. **Comparison of CopySeq copy-number genotyping with the read-counting approach by Alkan and coworkers.** Concordance in copy-number assignments* and number of loci assessed^#^ with Affymetrix arrays were previously published, and obtained from Table S8 in [6]. ^#^Note that Alkan *et al.* excluded few additional loci (besides excluding such overlapping SDs) on the basis of short-read mapping artifacts (see Supplementary Note IIId in [6]). We did not have access to this “additional-exclude-list”, but anticipate that the additional loci excluded in Alkan *et al.* are likely to be enriched in low-concordance events. Thus, we expect that the copy-number genotyping concordance indicated for CopySeq may be similar, or slightly higher, than indicated in the table, if we were to consider the “additional exclude list”. In any case, the “additional-exclude-list” was too small to change the trend we observed, i.e., copy-number genotypes generated with CopySeq displayed a higher concordance with Affymetrix arrays than Alkan *et al.* based copy-number assignments. Note further that discordance with the Affymetrix array-based copy-number assignments implies that either the array or the NGS-data based approaches inferred the locus copy-number incorrectly. In this regard, Alkan *et al.* identified two loci on chromosomes 7 and 19, respectively, in which read-counting suggested a likely locus-copy of “2”, while the Affymetrix arrays called these loci as “0” and “1”, respectively. Alkan *et al.* showed with additional data that these two loci were likely misclassified by the arrays [6]. CopySeq agreed with the Alkan *et al.* calls in both cases (*i.e.,* Copy-Seq inferred copy-number genotypes of “2” at both loci). The table below has not been corrected for these presumable Affymetrix array-based copy-number mis-assignments. We further excluded in our concordance estimations loci that were not genotyped in McCarroll *et al.,* i.e., locus copy-number genotypes reported as “NA” in [2]. ‘N/A’, not reported in [6].

| **Locus size cutoff**  **(kb)** | **Number of loci genotyped in McCarroll *et al*. (excluding SD loci)** | **Number of CopySeq calls concordant with McCarroll *et al.*** | **CopySeq  concordance in copy-number assignments  (%)** | **Alkan *et al.* concordance in copy-number assignments (%)** **[number of loci assessed in [6]]** |
| --- | --- | --- | --- | --- |
| No size cutoff | 992 | 961 | 96.9 | N/A |
| ≥1 | 981 | 953 | 97.2 | 80.2* [955^#^] |
| ≥5 | 555 | 542 | 97.7 | 82.0* [538^#^] |
| ≥10 | 290 | 282 | 97.2 | 88.7* [282^#^] |
| ≥20 | 129 | 127 | 98.4 | 93.8* [128^#^] |
| ≥40 | 58 | 57 | 98.3 | 96.5* [57^#^] |
| ≥100 | 11 | 11 | 100 | 100* [11^#^] |
