# Supplementary material for: Systematic Inference of Copy-Number Genotypes from Personal Genome Sequencing Data Reveals Extensive Olfactory Receptor Gene Content Diversity
Source: PLoS Comput Biol. 2010 Nov 11;6(11):e1000988. doi: 10.1371/journal.pcbi.1000988 (PMC2978733; doi:10.1371/journal.pcbi.1000988)
Supplement: Table S19 — qPCR validation of copy-number genotypes in 18 CNV loci on chromosome 1. (0.16 MB DOC) [file pcbi.1000988.s039.doc]

**Table S19. qPCR validation of copy-number genotypes in 18 CNV loci on chromosome 1.** qPCR did not measure absolute copy numbers, but rather relative copy-number differences between individuals, in loci marked by ‘^#^’. In the other loci, qPCR results were used to infer integer (absolute) copy number genotypes. CN, estimated copy-number. The validations were carried out in a parent offspring trio, and are listed for each CNV region in the following order: daughter (NA12878 or ‘12878’), father (NA12891), and mother (NA12892).

| **Primer Id** | **CNV Id** | **Genomic Coordinates (hg18)** | **CNV Size (bp)** | **Norm Ct** | **Norm ΔCt to 12878** | **qPCR CN** | **CopySeq CN** |
| --- | --- | --- | --- | --- | --- | --- | --- |
| q11 | CNVR479.2^#^ | 1:204239359-204366782 | 127,424 | 22.59 | 0 | 2^#^ | 2 |
| q11 | CNVR479.2^#^ | 1:204239359-204366782 | 127,424 | 22.53 | -0.056 | 2^#^ | 2 |
| q11 | CNVR479.2^#^ | 1:204239359-204366782 | 127,424 | 22.45 | -0.137 | 2^#^ | 2 |
| q5 | CNVR217.1 | 1:72538870-72584557 | 45,688 | 33.92 | 0 | 0 | 0 |
| q5 | CNVR217.1 | 1:72538870-72584557 | 45,688 | 33.77 | -0.143 | 0 | 0 |
| q5 | CNVR217.1 | 1:72538870-72584557 | 45,688 | 34.2 | 0.277 | 0 | 0 |
| q23 | 109 | 1:150822330-150853218 | 30,889 | 23.55 | 0 | 1 | 1 |
| q23 | 109 | 1:150822330-150853218 | 30,889 | 39.41 | 15.86 | 0 | 0 |
| q23 | 109 | 1:150822330-150853218 | 30,889 | 22.35 | -1.198 | 2 | 2 |
| q7 | CNVR299.4^#^ | 1:110016535-110046454 | 29,920 | 22.04 | 0 | 1^#^ | 1 |
| q7 | CNVR299.4^#^ | 1:110016535-110046454 | 29,920 | 21.94 | -0.098 | 1^#^ | 1 |
| q7 | CNVR299.4^#^ | 1:110016535-110046454 | 29,920 | 21.9 | -0.137 | 1^#^ | 1 |
| q20 | 88 | 1:110025907-110044476 | 18,570 | 24.65 | 0 | 1 | 1 |
| q20 | 88 | 1:110025907-110044476 | 18,570 | 37.53 | 12.879 | 0 | 0 |
| q20 | 88 | 1:110025907-110044476 | 18,570 | 24.79 | 0.14 | 1 | 1 |
| q9 | CNVR360.1 | 1:151026713-151037738 | 11,026 | 35.97 | 0 | 0 | 0 |
| q9 | CNVR360.1 | 1:151026713-151037738 | 11,026 | 23.38 | -12.593 | 1 | 1 |
| q9 | CNVR360.1 | 1:151026713-151037738 | 11,026 | 23.53 | -12.44 | 1 | 1 |
| q4 | CNVR138.1 | 1:34874894-34884557 | 9,664 | 23.49 | 0 | 1 | 1 |
| q4 | CNVR138.1 | 1:34874894-34884557 | 9,664 | 22.51 | -0.985 | 2 | 2 |
| q4 | CNVR138.1 | 1:34874894-34884557 | 9,664 | 23.59 | 0.099 | 1 | 1 |
| q24 | 122 | 1:167500598-167508390 | 7,793 | 22.63 | 0 | 2 | 2 |
| q24 | 122 | 1:167500598-167508390 | 7,793 | 22.51 | -0.126 | 2 | 2 |
| q24 | 122 | 1:167500598-167508390 | 7,793 | 23.65 | 1.019 | 1 | 1 |
| q6 | CNVR293.1 | 1:108534878-108538928 | 4,051 | 23.38 | 0 | 1 | 1 |
| q6 | CNVR293.1 | 1:108534878-108538928 | 4,051 | 22.29 | -1.095 | 2 | 2 |
| q6 | CNVR293.1 | 1:108534878-108538928 | 4,051 | 40 | 16.889 | 0 | 0 |
| q28 | 168 | 1:227883479-227886819 | 3,341 | 37.33 | 0 | 0 | 0 |
| q28 | 168 | 1:227883479-227886819 | 3,341 | 23.02 | -14.308 | 1 | 1 |
| q28 | 168 | 1:227883479-227886819 | 3,341 | 23.08 | -14.244 | 1 | 1 |
| q3 | CNVR90.1 | 1:16024861-16028089 | 3,229 | 23.34 | 0 | 1 | 1 |
| q3 | CNVR90.1 | 1:16024861-16028089 | 3,229 | 37.82 | 14.482 | 0 | 0 |
| q3 | CNVR90.1 | 1:16024861-16028089 | 3,229 | 22.15 | -1.193 | 2 | 2 |
| q26 | 158 | 1:208148236-208150607 | 2,372 | 22.17 | 0 | 2 | 2 |
| q26 | 158 | 1:208148236-208150607 | 2,372 | 22.04 | -0.126 | 2 | 2 |
| q26 | 158 | 1:208148236-208150607 | 2,372 | 23.02 | 0.851 | 1 | 1 |
| q1 | CNVR51.1 | 1:7491802-7494108 | 2,307 | 23.76 | 0 | 1 | 2 |
| q1 | CNVR51.1 | 1:7491802-7494108 | 2,307 | 23.64 | -0.118 | 1 | 2 |
| q1 | CNVR51.1 | 1:7491802-7494108 | 2,307 | 22.81 | -0.957 | 2 | 2 |
| q14 | CNVR539.1 | 1:230525246-230526864 | 1,619 | 22.77 | 0 | 2 | 2 |
| q14 | CNVR539.1 | 1:230525246-230526864 | 1,619 | 23.73 | 0.954 | 1 | 2 |
| q14 | CNVR539.1 | 1:230525246-230526864 | 1,619 | 22.7 | -0.073 | 2 | 2 |
| q16 | CNVR561.1 | 1:238459885-238461481 | 1,597 | 22.32 | 0 | 2 | 2 |
| q16 | CNVR561.1 | 1:238459885-238461481 | 1,597 | 22.43 | 0.106 | 2 | 2 |
| q16 | CNVR561.1 | 1:238459885-238461481 | 1,597 | 23.33 | 1.011 | 1 | 1 |
| q10 | CNVR450.1 | 1:192617034-192618267 | 1,234 | 22.62 | 0 | 2 | 2 |
| q10 | CNVR450.1 | 1:192617034-192618267 | 1,234 | 23.64 | 1.023 | 1 | 1 |
| q10 | CNVR450.1 | 1:192617034-192618267 | 1,234 | 22.6 | -0.025 | 2 | 2 |
| q2 | CNVR65.1 | 1:10405137-10406094 | 958 | 24.54 | 0 | 1 | 2 |
| q2 | CNVR65.1 | 1:10405137-10406094 | 958 | 23.42 | -1.127 | 2 | 2 |
| q2 | CNVR65.1 | 1:10405137-10406094 | 958 | 24.59 | 0.047 | 1 | 2 |
| q12 | CNVR483.1 | 1:205359125-205359831 | 707 | 37.15 | 0 | 0 | 0 |
| q12 | CNVR483.1 | 1:205359125-205359831 | 707 | 36.75 | -0.391 | 0 | 0 |
| q12 | CNVR483.1 | 1:205359125-205359831 | 707 | 35.03 | -2.115 | 0 | 0 |
