# Supplementary material for: Systematic Inference of Copy-Number Genotypes from Personal Genome Sequencing Data Reveals Extensive Olfactory Receptor Gene Content Diversity
Source: PLoS Comput Biol. 2010 Nov 11;6(11):e1000988. doi: 10.1371/journal.pcbi.1000988 (PMC2978733; doi:10.1371/journal.pcbi.1000988)
Supplement: Table S20 — qPCR primer sequences. (0.06 MB DOC) [file pcbi.1000988.s040.doc]

Table S20. qPCR primer sequences

| **Primer Id** | **Primer Sequence** |
| --- | --- |
| q1F | GGATAATGcaggatagcacca |
| q1R | agctactagggtgtccctcca |
| q2F | ACCTGTTACAACTCCCCCACT |
| q2R | TCAGGATTCTACCGAATCAGAA |
| q3F | CTGCGCATGCTACAATACAAG |
| q3R | CGTGACCACCACCCTTCAT |
| q4F | CTTCTACACCAAGCCCTACCC |
| q4R | atcgatggttgctgttagtgg |
| q5F | gcttgggaattagcaccctac |
| q5R | gcacttgtattggcagtggtt |
| q6F | GCCAACATCTTGAGAGCAATC |
| q6R | GTTGACCAGCGTTACCGTTTA |
| q7F | gatccccaaagcgcttactac |
| q7R | AATGATCCGTGtaccttgctg |
| q9F | GTGTGAACCCAGAAAAGTCCA |
| q9R | GGTCCTTGATGTGTCAGCACT |
| q10F | tcagctgcttggagctctact |
| q10R | TGAATTTGTTGTGCGGTGTAA |
| q11F | tgacgcttctcagctcttctc |
| q11R | TGGGGTCAACAGTAGTTCAGG |
| q12F | ctctgccctgacctttctagc |
| q12R | atggcccataaaagacggtag |
| q14F | taaactgtccctgcaaaatgc |
| q14R | ATTCAGCAGCGAAtctaacca |
| q16F | TAATGCCAGCCTTCTTCTCAA |
| q16R | AATGTGGAACGCAAAGAGATG |
| q20F | TGCATTCGTTCATGTGACAGT |
| q20R | tcccataagcaagagcagaga |
| q23F | TCCACAGCCTCCTAGAGAACA |
| q23R | GGGTCCCCTGCTAGATACTTG |
| q24F | atgaacgctgtcgtctcaatc |
| q24R | aataaagggcaagcacaacct |
| q26F | gtgggaagatagggcagagtc |
| q26R | agctggacatgctaggattga |
| q28F | gtgtatgtttgacgcccttgt |
| q28R | cgtaccggcaaaaagacagta |
| 11J2P_F | CTGTCTTGTGCTCCAATACCTG |
| 11J2P_R | TTATGCCTTCCTAAGCCTGAAG |
| 13C2_F | GGCTGAACAGGTAGAGGAAGC |
| 13C2_R | ATCATGCTTGTGGCCACAAC |
| 4H12P_F | CGACGCAAAGCTCTCTCTAC |
| 4H12P_R | GAAAGTAGTGGATGGACGGAG |
| 4H6P_F | tTGATAGgCTTTActaTGCTTAccAc |
| 4H6P_R | GGAGAAATAAGACAAAGAAcATCaATt |
| 4Q3_F | GCATTCCATTGCCTTGTTAAG |
| 4Q3_R | CGTCATCAAAGTGGTTTTTACC |
