# Supplementary material for: Systematic Inference of Copy-Number Genotypes from Personal Genome Sequencing Data Reveals Extensive Olfactory Receptor Gene Content Diversity
Source: PLoS Comput Biol. 2010 Nov 11;6(11):e1000988. doi: 10.1371/journal.pcbi.1000988 (PMC2978733; doi:10.1371/journal.pcbi.1000988)
Supplement: Table S21 — Concordance of CopySeq and fluorescent in situ hybridization (FISH) results. (0.05 MB DOC) [file pcbi.1000988.s041.doc]

**Table S21. Concordance of CopySeq and fluorescent *in situ* hybridization (FISH) results.** CopySeq was applied on the published NA18507 genome [7] to infer locus copy-numbers in CNV regions previously validated by with FISH in [6]. We assessed all loci ascertained by FISH in [6], where the FISH probe intersected with no SD, or a single SD (see Supplementary Notes). The clone IDs are from [6]. Probe coordinates were converted to hg18 with liftOver. The position of the paralogous SD within the ~40kb FISH probes was retrieved from the UCSC browser’s SD track. CopySeq was carried out for the region encompassing the FISH probe. In cases where an SD intersected with the FISH probe, we also genotyped the corresponding paralogous (SD) locus with CopySeq, since FISH probes are known to hybridize to paralogous loci in the genome such as SDs [15]. As evident from the table below, CopySeq was consistent with the FISH hybridization in 80% (4/5) cases (*i.e.*, the FISH results reflected the sum of locus-specific copy-counts measured by CopySeq; see below). **%**Measurement is locus-specific, *i.e.*, copy-number genotypes reflect the copy-number of each specific paralog. **^#^**Measured copy-number is thought to reflect the sum across paralogous loci, in cases were SD is present (if no SD is present, the measurement reflects the locus-specific copy-number). *SD intersects with FISH probe (in all cases indicated with a ‘*’ >80% of the FISH probe was spanned by a single, large SD).

| **Clone ID** | **Position (hg18) (clone)** | **Position (hg18) (SD-paralog)** | **FISH^#^**  **CN (clone)** | **CopySeq%**  **CN**  **(locus)** | **CopySeq%**  **CN**  **(paralog)** | **CopySeq**  **CN**  **(locus + paralog)** |
| --- | --- | --- | --- | --- | --- | --- |
| WIBR2-2553B23 G248P83461A12* | chr8:7,697,239-7,734,464 | chr8:7,300,000-7,340,000 | 3 | 1 | 1 | 2 |
| WIBR2-2992K17 G248P89716F9 | chr15:20,384,910-20,425,229 | - | 1 | 1 | - | 1 |
| WIBR2-3400B16 G248P802583A8* | chr16:68,708,494-68,749,105 | chr16:72,923,500-72,963,500 | 5 | 3 | 2 | 5 |
| WIBR2-1797D06 G248P85943B3* | chr17:41,870,497-41,906,290 | chr17:42,020,000-42,060,000 | 2 | 1 | 1 | 2 |
| WIBR2-1854B21 G248P85429A11* | chr17:42,055,754-42,093,334 | chr17:41,800,000-41,840,000 | 2 | 1 | 1 | 2 |
